# Rapid Diversification of *FoxP2* in Teleosts through Gene Duplication in the Teleost-Specific Whole Genome Duplication Event

**DOI:** 10.1371/journal.pone.0083858

**Published:** 2013-12-09

**Authors:** Xiaowei Song, Yajun Wang, Yezhong Tang

**Affiliations:** 1 Department of Herpetology, Chengdu Institute of Biology, Chinese Academy of Sciences, Chengdu, Sichuan, China; 2 College of Life Science, Sichuan University, Chengdu, Sichuan, China; 3 University of Chinese Academy of Sciences, Beijing, China; INRA, France

## Abstract

As one of the most conserved genes in vertebrates, *FoxP2* is widely involved in a number of important physiological and developmental processes. We systematically studied the evolutionary history and functional adaptations of *FoxP2* in teleosts. The duplicated *FoxP2* genes (*FoxP2a* and *FoxP2b*), which were identified in teleosts using synteny and paralogon analysis on genome databases of eight organisms, were probably generated in the teleost-specific whole genome duplication event. A credible classification with *FoxP2*, *FoxP2a* and *FoxP2b* in phylogenetic reconstructions confirmed the teleost-specific *FoxP2* duplication. The unavailability of *FoxP2b* in *Danio rerio* suggests that the gene was deleted through nonfunctionalization of the redundant copy after the Otocephala-Euteleostei split. Heterogeneity in evolutionary rates among clusters consisting of *FoxP2* in Sarcopterygii (Cluster 1), *FoxP2a* in Teleostei (Cluster 2) and *FoxP2b* in Teleostei (Cluster 3), particularly between Clusters 2 and 3, reveals asymmetric functional divergence after the gene duplication. Hierarchical cluster analyses of hydrophobicity profiles demonstrated significant structural divergence among the three clusters with verification of subsequent stepwise discriminant analysis, in which FoxP2 of *Leucoraja erinacea* and *Lepisosteus oculatus* were classified into Cluster 1, whereas FoxP2b of *Salmo salar* was grouped into Cluster 2 rather than Cluster 3. The simulated thermodynamic stability variations of the forkhead box domain (monomer and homodimer) showed remarkable divergence in FoxP2, FoxP2a and FoxP2b clusters. Relaxed purifying selection and positive Darwinian selection probably were complementary driving forces for the accelerated evolution of *FoxP2* in ray-finned fishes, especially for the adaptive evolution of *FoxP2a* and *FoxP2b* in teleosts subsequent to the teleost-specific gene duplication.

## Introduction

**Figure 1:**
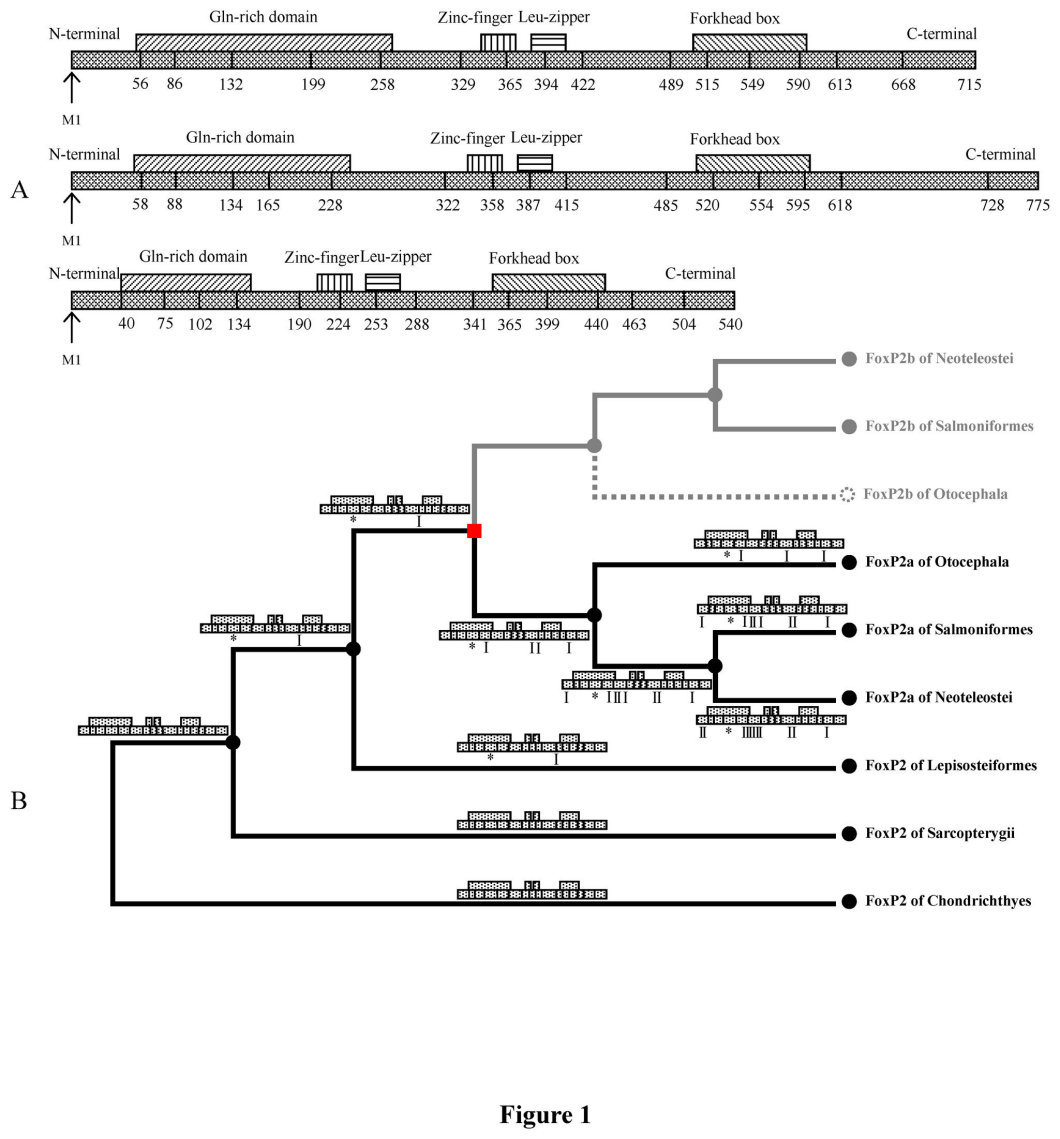
Schematic drawings and predicted evolutionary changes of FoxP2, FoxP2a and FoxP2b. (A) FOXP2 in *Homo sapiens* (top), FoxP2a in *Oryzias latipes* (middle) and FoxP2b in *Oryzias latipes* (bottom). M1 – Methionine coded by initiator; Numbers – Amino acid sites linking exons. (B) Branches of FoxP2b are shown in grey. Red square node indicates teleost-specific FoxP2 duplication. Symbols of ‘*’ and ‘I’ under schematic drawings represent losses of poly-Glutamine and inserts, respectively. The square dotted branch indicates lineage-specific deletion of FoxP2b in Otocephala. Despite a comparatively short length, exon-compositions of FoxP2b in euteleosts are still obscure.

*FoxP2* is a key transcription factor gene in the *FoxP* subfamily [1]. The gene possesses multiple functionally important domains, including the zinc-finger, leucine-zipper and forkhead box (Figure 1A). Genetic studies show that when *FOXP2* is rendered dysfunctional in humans, seen in cases of pathogenic translocation, missense mutation (R553H) and nonsense mutation (R328X), severe disorders of speech and language can result [2,3]. The gene is also likely involved in vocal control in zebra finches, mice and bats [4-7]. In addition, it has been found that *Foxp2* plays a nonessential role in the production of innate emotional vocalizations in mouse pups [8]. Based on these findings, it is reasonable to infer that the role of *FoxP2* in vocal communication is a derived function as opposed to an ancestral one. Aside from its role in vocal communication, *FoxP2* also plays a pleiotropic role in cell differentiation, signal transduction, organogenesis (e.g. lungs), and neural circuit plasticity in the central nervous system (CNS) [3-6,9-16]. 

Nevertheless, basic functions of the gene remain unclear due to limited studies. Functional studies of *FoxP2* have been mainly performed on mammals and birds but have seldom been conducted on other vertebrates (i.e. reptiles, amphibians and fishes) [11,17-21]. The conservative expression patterns of *FoxP2* seen during embryonic development in homologous brain regions of mammals, birds, frogs, and fishes [17] imply that *FoxP2* is associated with similar functions in the CNS of vertebrates. In mice, *Foxp2* is essential for lung development, especially for postnatal lung alveolarization [12,22]. *FoxP2* of medaka shows weak transcriptional activity, *in vitro*, for regulation of the lung-specific *CC10* gene of mice[19].

Despite the fact that *FoxP2* is one of the most conserved genes in vertebrates [23-25], structures and functions of the gene in teleosts probably show substantial divergence [18,19,21]. It is still an open question about how and why such substantial divergence occurred. Either positive selection or relaxation of purifying selection may have forced the divergence of the gene. In addition, gene duplication often results in diversifying natural selection pressures on the duplicated genes, further forcing their fast evolution [26]. Since the fish-specific whole genome duplication, which is also known as the 3^rd^ round of whole genome duplication (3R-WGD), in the common ancestor of teleosts seems to be correlated with species diversification of teleosts [27], we hypothesize that *FoxP2* possibly has been duplicated in teleosts. Therefore, the logical existence of teleost-specific *FoxP2* paralogs should be investigated further. In addition, the teleost swim bladder has been considered to be a homologous organ to the lung on the basis of the consistency of both developmental blastema and molecular pathways, despite the fact that its main function is buoyancy regulation rather than respiration [28,29]. Thus, the divergence of *FoxP2* in teleosts may coincide with the adaptation of the swim bladder.

The present study aims to shed light on the underlying mechanisms from which the remarkable divergence of *FoxP2* in ray-finned fishes, particularly teleosts, resulted. We systematically investigated the evolutionary history and functional adaptations of *FoxP2* through a series of analyses on phylogenetic relationships, natural selection pressures, and divergence of structures and functions. The study concerning rapid evolution of *FoxP2* in ray-finned fishes will prompt further explorations of *FoxP2*’s functional adaptations.

## Materials and Methods

### Molecular biological methods for data collection

The experimental animals included *Colisa lalia*, *Pachytriton labiatus*, *Rana daunchina*, *Phrynocephalus vlangalii* and *Gekko gecko* (Information S1). All procedures used were approved by the Animal Care Committee of Chengdu Institute of Biology, Chinese Academy of Sciences. Animals were first anesthetized with pelltobarbitalum natricum before sacrifice. Total RNA was extracted from the brains of the animals by using the TRIzol reagent (Invitrogen). The cDNA templates were synthesized by RevertAid First Strand cDNA Synthesis Kit (Fermentas). The primers for the subsequent polymerase chain reaction (PCR) were designed by utilizing Primer Premier 5 and Oligo 6 softwares in conjunction with Primer 3 plugged into Biology Workbench of San Diego Supercomputer Center (http://workbench.sdsc.edu) and synthesized by Sangon Biotech (Shanghai) Co., Ltd (Information S1). PCRs were performed using EasyTaq DNA polymerase or TransTaq HiFi DNA polymerase (Beijing TransGen Biotech Co., Ltd.). PCR products were purified by Axyprep DNA Gel Extraction Kit (AxyGEN) and cloned into the pTA2 vector (Toyobo). Gene sequencing was carried out by Invitrogen Company (Shanghai). 

### *In silico* methods for data collection

We downloaded 18 coding sequences or fragments from GenBank or Ensembl directly (Information S1). We obtained 19 additional coding sequences or fragments through screening genomes and assessed the results using the Basic Local Alignment Search Tool (BLAST) in the National Center for Biotechnology Information (NCBI) [30] (Information S1). The data bases for screening *FoxP2* included the NCBI, the USCS Genome Bioinformatics website (http://genome.ucsc.edu/) [31] and the Elephant Shark Genome Project (http://esharkgenome.imcb.a-star.edu.sg/) [32,33] (Information S1). Since amino acid sequences of *FoxP2* in vertebrates are highly conserved, we used protein queries (*Danio rerio* [DQ061052.1], *Xenopus laevis* [BC170268.1], *Taeniopygia guttata* [AY395709.1] and *Mus musculus* [NM_053242.4]) to predict *FoxP2* coding sequences through genome screenings. According to the results of phylogenetic reconstruction as well as synteny and paralogon analysis, the formerly named *FoxP2* of teleosts was identified to be *FoxP2a*, as one of two paralogs (i.e. *FoxP2a* and *FoxP2b*). The nomenclature for these genes was therefore corrected in the present study to avoid confusion. Due to the drastic divergence and unclear characteristics of *FoxP2b*, we predicted *FoxP2b* coding sequences by a two step approach: first, searching contig locations with queries of FoxP2b in *Gadus morhua* (GW852741.1) and *Oryzias latipes* (XM_004069881.1); second, screening out the coding sequences with GENSCAN (http://genes.mit.edu/GENSCAN.html) [34].

### Synteny and paralogon analysis

**Figure 2:**
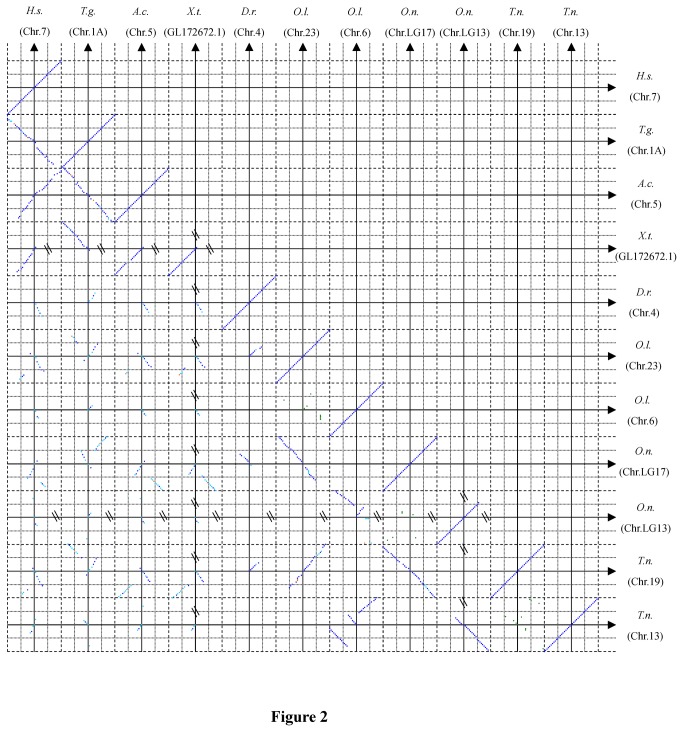
Pairwise comparisons of 40 (20 upstream and 20 downstream) protein-coding genes flanking FoxP2, *FoxP2a* or *FoxP2b* among eight vertebrates. The solid lines represent chromosomes (or scaffold) of species, i.e. Chr. 7 of *Homo sapiens* (H.s.), Chr. 1A of *Taeniopygia guttata* (T.g.), Chr. 5 of *Anolis carolinensis* (A.c.), GL172672.1 of *Xenopus tropicalis* (X.t.), Chr. 4 of *Danio rerio* (D.r.), Chr. 23 and 6 of *Oryzias latipes* (O.l.), Chr. LG17 and LG13 of *Oreochromis niloticus* (O.n.) and Chr. 19 and 13 of *Tetraodon nigroviridis* (T.n.). The arrow head of each solid line points toward the forward direction of the corresponding chromosome. Crossing points of the solid lines indicate gene loci for FoxP2, *FoxP2a* or *FoxP2b*. Short dashed lines and dotted lines show the borders of pairwise comparisons and the measuring scale of gene loci, respectively. The points with blue, sky blue, orange and green indicate that the corresponding gene pairs are one-to-one orthologous, one-to-many or many-to-many orthologous, possibly orthologous and intraspecific paralogous, respectively.

To investigate whether *FoxP2* paralogs exclusively exist in teleosts, we conducted synteny and paralogon analysis to determine homologous (orthologous or paralogous) relationships of sequences involved [35,36]. The synteny and paralogon analysis of *FoxP2* loci was performed on genome databases of eight representative species in Ensembl: *Homo sapiens* (GRCh37), *Taeniopygia guttata* (taeGut3.2.4), *Anolis carolinensis* (AnoCar2.0), *Xenopus tropicalis* (JGI_4.2), *Danio rerio* (Zv9), *Oryzias latipes* (MEDAKA1), *Oreochromis niloticus* (Orenil1.0) and *Tetraodon nigroviridis* (TETRAODON8). Twenty upstream and 20 downstream protein-coding genes flanking *FoxP2*, *FoxP2a* and *FoxP2b* were selected for the synteny and paralogon analysis. The homologous relationships among sequences were determined using the prediction method in Ensembl (http://www.ensembl.org) [37]. Results of pairwise region comparisons were integrated into one figure (Figure 2 and Information S2).

### Phylogenetic reconstructions

**Figure 3:**
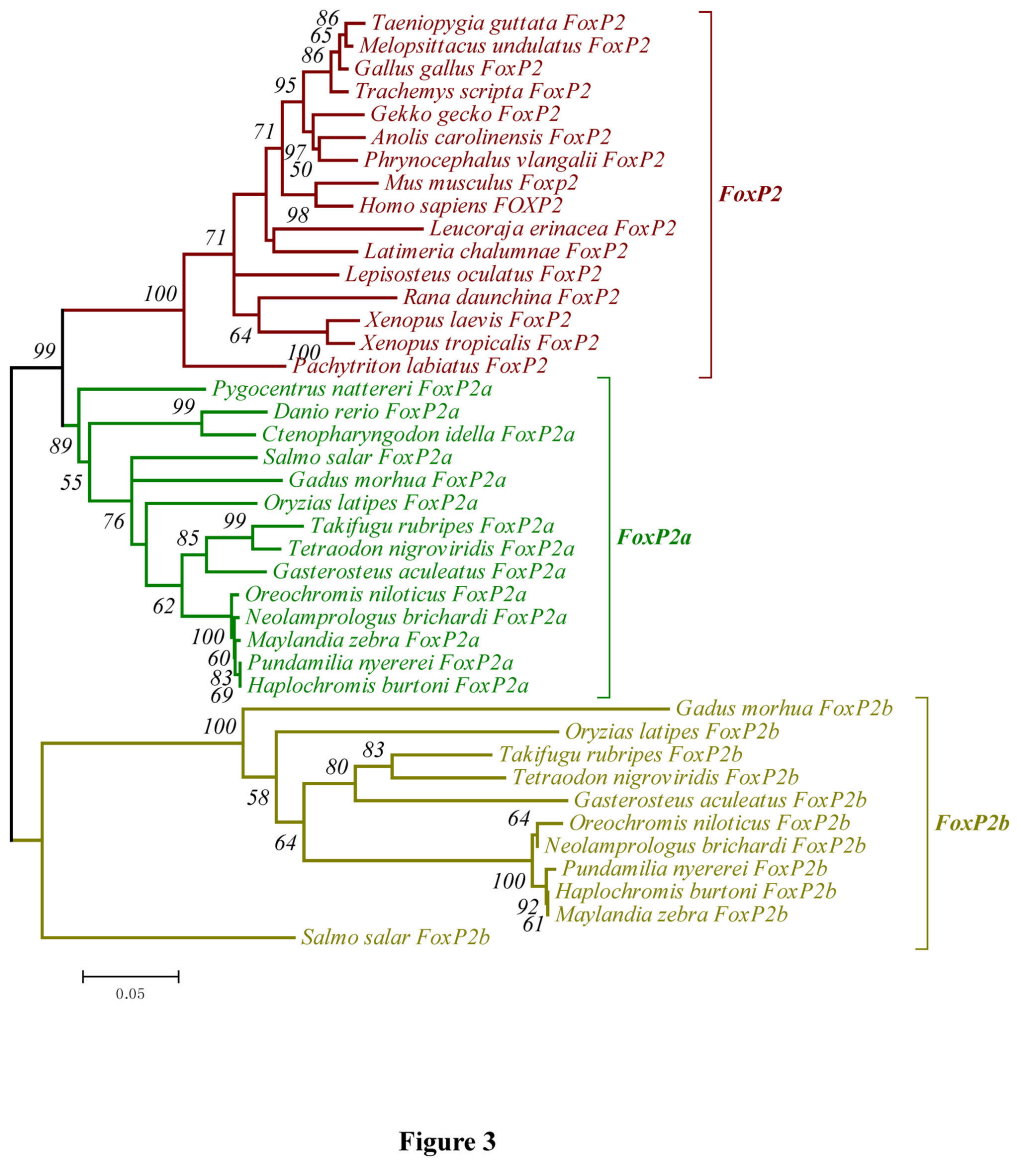
NJ tree based on nucleotide sequences in Data Set 1. Bootstrap values lower than 50 on branches are not shown.

**Figure 4:**
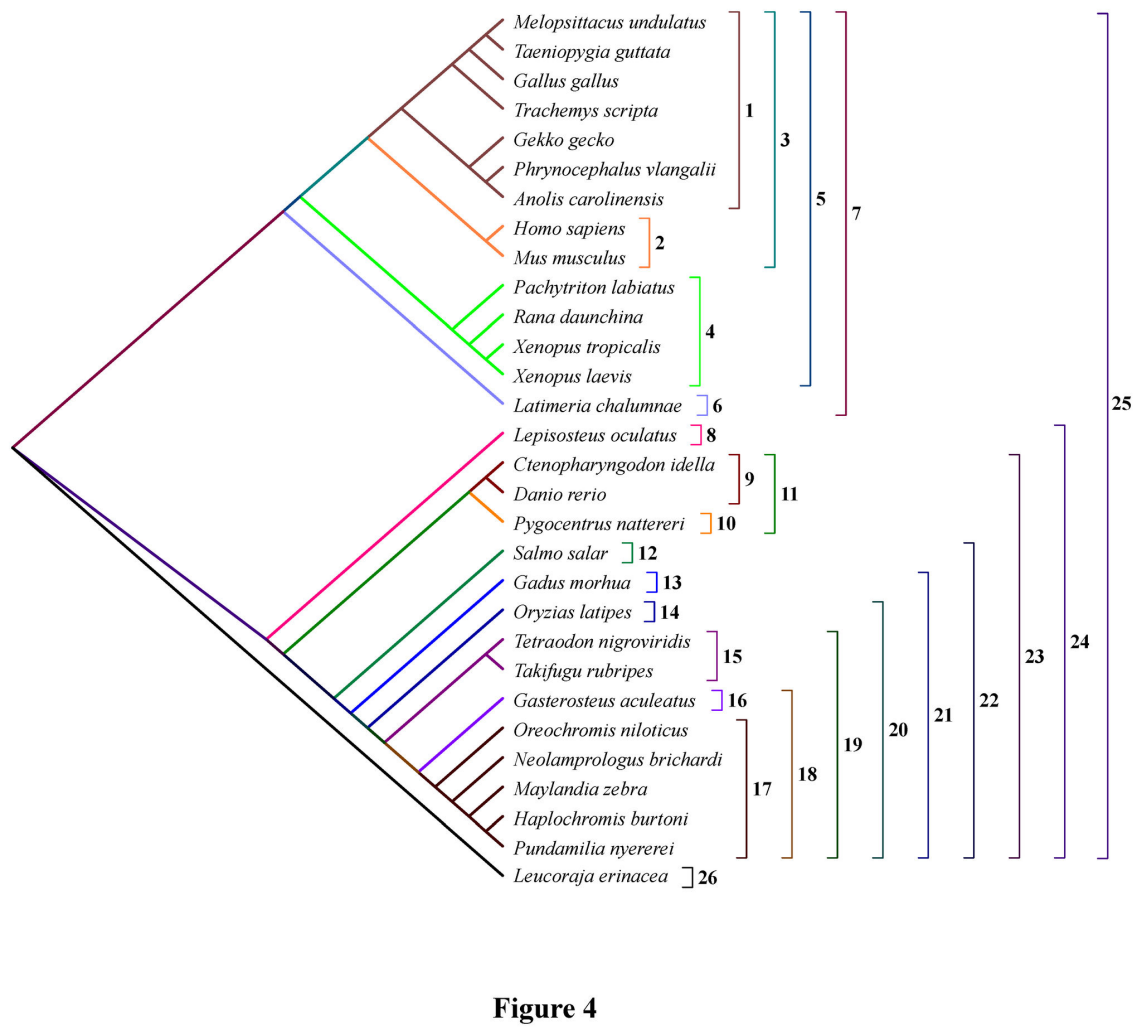
Phylogenetic topology tree of 30 species. 1, Sauropsida; 2, Mammalia; 3, Amniota; 4, Amphibia; 5, Tetrapoda; 6, Coelacanthiformes; 7, Sarcopterygii; 8, Lepisosteiformes; 9, Cypriniformes; 10, Characiformes; 11, Otocephala; 12, Salmoniformes; 13, Gadiformes; 14, Beloniformes; 15, Tetraodontiformes; 16, Gasterosteiformes; 17, Perciformes; 18, unnamed; 19, Percomorpha; 20, Acanthopterygii; 21, Neoteleostei; 22, Euteleostei; 23, Teleostei; 24, Actinopterygii; 25, Osteichthyes; 26, Chondrichthyes.

In order to facilitate comparison and discussion, all site and exon numbers of FoxP2, FoxP2a and FoxP2b were made to correspond to the numbers of the counterparts in human FOXP2 (Figure 1A). Multiple sequence alignments were executed with clustalW in the BioEdit software (version 7.0.5.3) [38,39] (Information S3). Since exon composition and the functions of *FoxP2b* were unknown, we only phylogenetically analyzed the conserved forkhead box domain, i.e. exons 12, 13, 14, 15 and partial exon11, in Data set 1 (consisting of 41 *FoxP2*, *FoxP2a* and *FoxP2b* fragments) (Figure 3 and Information S4). Phylogenetic trees generated with a single intraspecific paralogous gene were able to avoid polytomy. In view of the occurrence of excessive divergence in teleost *FoxP2b*, we reconstructed gene trees using the *FoxP2* and *FoxP2a* coding sequences (i.e. Data set 2) to infer the species tree (Figure 4 and Information S4). Since exons 6, 16 and partial exon8 were unavailable in some species, we deleted these exons and the low complexity poly-glutamine (poly-Q) region (i.e. exon5) in Data set 2. We assessed substitution saturation of these data sets before the phylogenetic analyses using DAMBE software (version 5.2.57) [40]. Maximum Likelihood (ML) and Neighbor-Joining (NJ) trees were constructed based on nucleotide or amino acid sequences using MEGA software (version 5.10) [41,42]. We used the general time reversible (GTR) model with gamma distributed plus invariant sites (G+I) and the maximum composite likelihood (MCL) method [43] for ML and NJ trees based on nucleotide sequences, respectively. The models/methods for both the ML and NJ trees based on amino acid sequences were the Jones-Taylor-Thornton (JTT) model with G [44]. The bootstrap method (1000 replications) was used in phylogenetic reconstructions for the ML and NJ trees [45]. Bayesian phylogenetic analysis was performed with MrBayes software (version 3.1.2) [46]. We partitioned nucleotide coding sequences into three datasets (first, second and third codon position sites) which had some unlinking parameters (statefreq, revmat, shape and pinvar) across the partitions. The analyses by MrBayes were based on GTR+G+I with generations (10,000,000), sample frequency (1000) and temperature (0.05). All other options were left at default settings.

### Analysis of functional divergence

The coefficient of functional divergence (θ_λ_) is an efficient parameter for testing type I functional divergence after gene duplication [47]. Type I functional divergence refers to different functional constraints in paralogous gene clusters after gene duplication. The method used here is based on the principle that the coefficient of evolutionary rate correlation (*r*_λ_) reflects functional divergence in the gene clusters. Specifically, the coefficient of functional divergence is calculated with the formula, θ_λ_ = 1- *r*_λ_, using DIVERGE software (version 1.04) [47]. A statistical significance level of θ_λ_ > 0 is computed using a likelihood ratio test (LRT) through comparing whether δ = -2 * ln(LR) is greater than the value of the χ^2^_[1]_ distribution at a significance level of 0.05. LR represents the ratio of the maximum likelihood that θ_λ_ > 0 to that of θ_λ_ = 0. In addition, the critical amino acids that are responsible for the type I functional divergence can be identified using a reasonable cutoff value (> 1) for the posterior probability ratio based on the hidden Markov model. 

**Figure 5:**
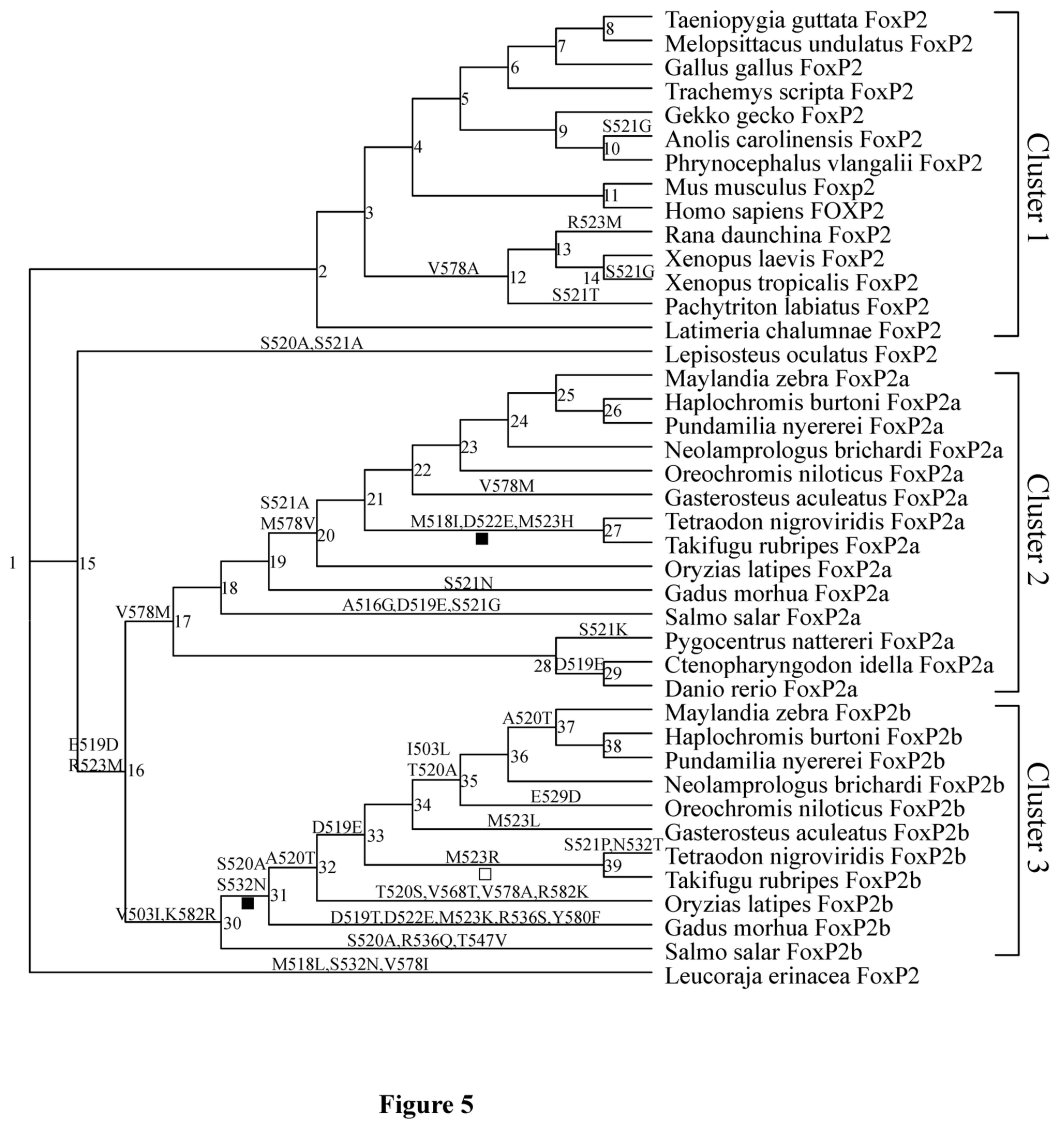
The phylogenetic topology tree for functional divergence and natural selection pressures tests in Data Set 1. Numbers adjacent to nodes represent ancestral FoxP2, FoxP2a and FoxP2b. Forkhead domain evolutionary changes between neighboring nodes are listed above the branches. The filled square under a branch indicates that the gene has undergone positive selection in the evolutionary status; the blank square represents that the gene has experienced accelerated evolution.

**Figure 6:**
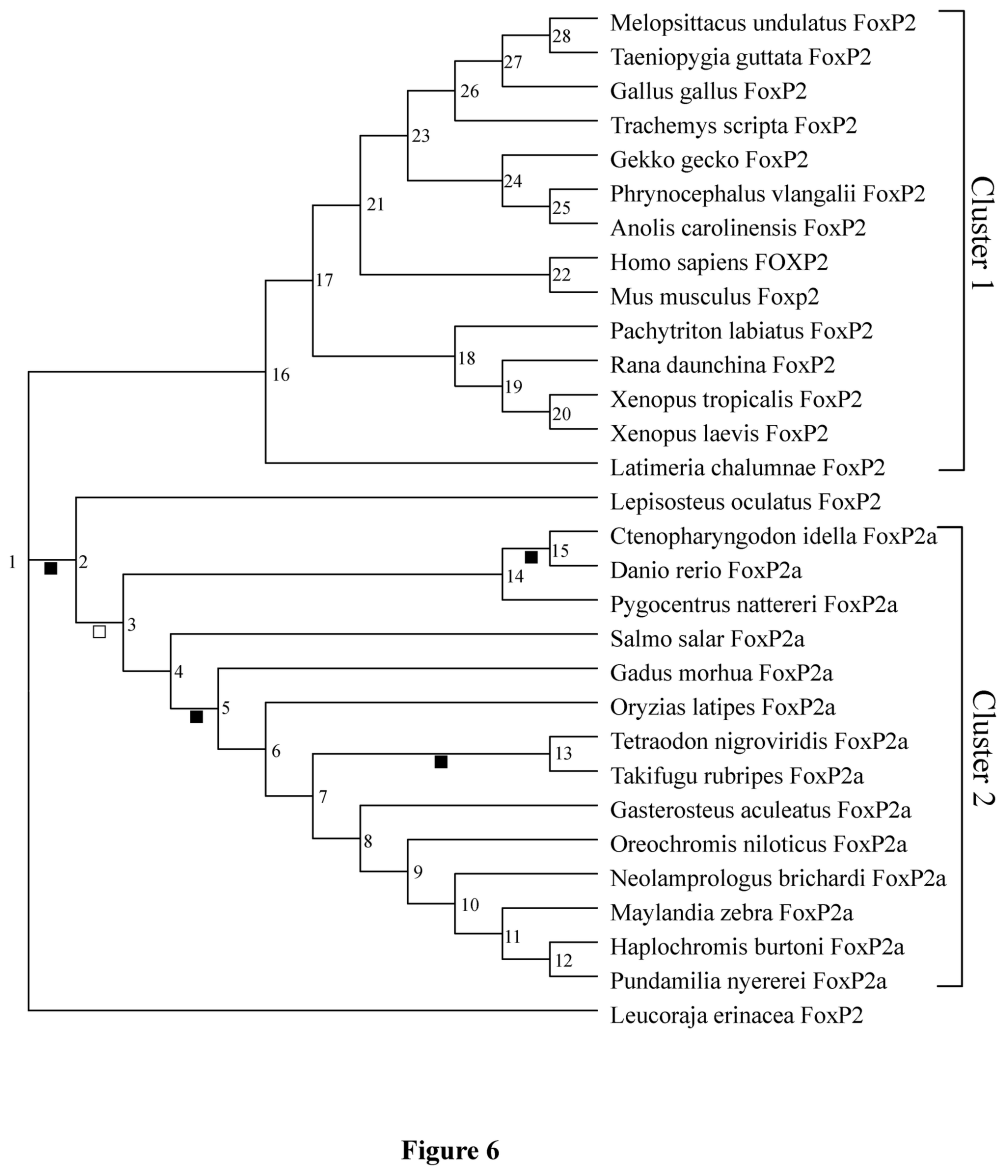
The phylogenetic topology tree for functional divergence and natural selection pressures tests in Data Set 2. Numbers adjacent to nodes represent ancestral FoxP2 and FoxP2a. The filled square under a branch indicates that the gene has undergone positive selection in the evolutionary status; the blank square indicates that the gene has experienced accelerated evolution.

We tested the functional divergence in Data set 1 among different gene clusters, i.e. Cluster 1 (*FoxP2* of Sarcopterygii), Cluster 2 (*FoxP2a* of Teleostei) and Cluster 3 (*FoxP2b* of Teleostei) based on the teleost lineage specific *FoxP2* duplication in the ancestral 3R-WGD event (Figure 5 and Information S5). A stringent cutoff value (> 2.33) for the posterior probability ratio was used, i.e. a posterior probability of a site-specific rate difference (> 0.7). Since the teleost *FoxP2a* exons surrounding functionally important domains (zinc-finger, leucine-zipper and forkhead box) show greater divergence than these functional domains, we also tested the functional divergence between Cluster 1 and Cluster 2 in Data set 2 to determine if the two clusters exhibit homogeneous evolutionary rates in an extensive region (Figure 6 and Information S5). 

### Analysis of structural divergence

To investigate the functional adaptations of a protein, the evolutionary changes of its molecular structures must first be studied. High correlation between sequence similarity and structure similarity [48] indicates that structural traits (e.g. charge, polarity and hydropathy) of amino acids in a protein determine its higher level structures. Thus, the hydrophobicity values of amino acids calculated with the Kyte-Doolittle method were utilized to evaluate the structure of FoxP2, FoxP2a and FoxP2b [49]. The amino acid sequences in Data sets 1 and 2 were transformed into hydrophobicity profiles composed of normalized hydrophobicity values from 0 to 1. In addition, we defined ‘gap’ sites in sequence alignments as a normalized hydrophobicity value of 0.5. For the sake of capturing general differences between protein structures, we executed hierarchical cluster analyses based on four kinds of pairwise distances (i.e. Cityblock, Correlation, Cosine and Euclidean) using a Matlab program (MathWorks) (Information S6). The average-linkage cluster method was chosen for the cluster analyses. The cophenetic correlation coefficients between the cophenetic distances obtained from a cluster tree and the original distances used to construct the tree can be used to assess how faithfully the tree represents the original distances between sequences. In addition, we used the inconsistency coefficient as a measure of dissimilarity between downward links (branches) or clusters connected by a link. Although an inconsistency coefficient threshold could be arbitrarily selected to classify the sequences, we set a flexible cutoff value (0.8) greater than the baseline value (0.707) to avoid an exhaustive classification. The depth denoting the number of levels of the cluster tree for calculating the inconsistency coefficient was set to 2. 

**Table 1:** Estimates of the coefficient of functional divergence (θ_λ_) among clusters.

	Clusters 1 and 2	Clusters 1 and 3	Clusters 2 and 3	Clusters 1 and 2 (’)
θ_λ_	0.267	0.578	0.706	0.402
α	0.071	0.401	0.321	0.220
SE of θ_λ_	0.196	0.322	0.174	0.106
δ	1.845	3.215	16.395*	14.261*

The rightmost column with an apostrophe lists results from Data set 2; the other three columns list results from Data set 1. The starred δ value indicates the column is statistically significant. α – Gamma shape parameter of rate variation among sites; SE – Standard error; δ – LRT value of θ_λ_; Cluster 1 – *FoxP2* of Sarcopterygii; Cluster 2 – *FoxP2a* of Teleostei; Cluster 3 – *FoxP2b* of Teleostei.

Furthermore, we performed a stepwise discriminant analysis using SPSS software (version 20) to evaluate the consistency of the classification in the four cluster trees constructed with each data set. The variable selection method used in the discriminant analysis was Mahalanobis distance. The prior probability for groups was computed according to size. We kept the F values for entry or removal of variables and the covariance matrix for classification as defaults. Coefficients for both the canonical discriminant function and Fisher’s linear discriminant function were computed. Finally we evaluated the classification results by using the percentage of original grouped cases and cross-validated grouped cases correctly classified. In Data set 1, we focused on the rationalization of groupings for FoxP2 of *Leucoraja erinacea*, *Lepisosteus oculatus* and *Rana daunchina*, as well as FoxP2b of *Salmo salar* and *Gadus morhua*. The previous three clusters (i.e. Cluster 1, Cluster 2 and Cluster 3) were used to discriminate these sequences. With Data set 2, we investigated whether FoxP2 of *Leucoraja erinacea* and *Lepisosteus oculatus* were grouped together with Cluster 1 or with Cluster 2. In order to study whether the classification was a remaining trace after a long-term evolutionary history, we also used the three clusters to discriminate hydrophobicity profiles of ancestral sequences from Data sets 1 and 2, which were inferred with a Dayhoff substitution rate matrix using the codeml program of PAML software [50].

### Evaluation of thermodynamic stability

The three-dimensional (3D) structure of the forkhead domain of human FOXP2 (PDB: 2A07) provides a model to investigate changes in the stability of the local 3D structure in different evolutionary scenarios. We applied FoldX (version 3.0 b5.1), a fast and effective computer algorithm for estimating the effect of mutations on stability [51], in order to predict the differences in total Gibbs free energy (DTE) from wild-type (i.e. human FOXP2) to mutants (i.e. extant sequences and predicted ancestral ones as described above). Amino acid changes in the forkhead domain between two neighboring nodes are shown in Figure 5. We repaired the model file to optimize the structure prior to these energy calculations. In addition to using chains A, B and J as a model for predicting the DTE of monomers, we extracted chains A, B, F and G to use as a model for evaluating the DTE of homodimers. For our calculations, we ignored water bridges and retained binding metals in crystals. We executed 20 energy prediction runs for each mutant to get an average difference in total Gibbs free energy (ADTE). If the ADTE of a mutant was greater than 0, the mutant was destabilizing; alternatively, if the ADTE was found to be less than 0, the mutant was stabilizing. According to the ADTE value of each mutant calculated with the same wild model, we determined the stability changes of monomers or homodimers in each evolutionary scenario using ADTE variations. Since the error margin in FoldX was approximately 0.5 kcal/mol [52], ADTE changes within this range were deemed insignificant.

### Test of natural selection pressures

The analyses of functional and structural divergence suggest that *FoxP2a* and *FoxP2b* in teleosts experienced dramatic evolutionary changes after gene duplication. Therefore, we tested natural selection pressures with Data set 1 based on the corresponding phylogenetic topology tree (Figure 5) using the codeml program implemented in PAML software (version 4.6) [50]. In view of the occurrence of heterogeneous evolutionary rates between conserved functional domains (e.g. the forkhead box) and the exons flanking those functional domains, we also tested natural selection pressures with Data set 2 to investigate whether the regions without Data set 1 underwent divergent natural selection pressures (Figure 6). Codon substitution models including site models, branch specific models and branch-site model A were utilized. The LRT was used to determine the fitness of the models to the data sets analyzed [50]. For the LRT, 2∆*l* (twice the log likelihood difference) between a model and its nested model was compared to the value of the χ^2^ distribution with degrees of freedom equal to the numerical difference between the free parameters of the two models. The nested model was rejected at a significance level of P < 0.05.

First, three pairs of site models were tested on the data sets and compared based on the LRT (Information S7). Comparison of Model 3 (discrete) to Model 0 (basic or one ratio) was used to test whether ω varied among sites. Comparisons of Model 2 (positive selection) to Model 1 (nearly neutral), and Model 8 (beta and ω > 1) to Model 7 (beta) were conducted in order to detect positively selected sites with the Bayes empirical Bayes (BEB) method [53]. The test of variable ω among different branches was performed by comparing the free ratios model (independent ω ratio for each branch) to Model 0. 

We used branch specific models to detect whether Cluster 1, Cluster 2 and Cluster 3 experienced different natural selection pressures (Information S7). Due to a significantly variable ω ratio among sites and branches, the branch-site model A, which allowed ω to vary among sites and across branches, was used to detect positive selection affecting a few sites along specific foreground lineages (Figure 5, Figure 6 and Information S7). Test 1 and Test 2 were performed for these branch-site models. The null models in Test 1 and Test 2 are Model 1 and branch-site model A with ω2 = 1 fixed, respectively [53]. Although Test 1 is unable to provide direct evidence of the existence of positive Darwinian selection, it can detect accelerated evolution attributed to potential positive selection or relaxed purifying selection. In contrast, Test 2 is a powerful test of positive natural selection. Positively selected sites inferred with the BEB procedure were replaced by the corresponding sites of human FOXP2. If *p* (one tail probability for χ^2^ distribution) value was not the statistically significant (P < 0.05), we neglected Test 2 and positively selected sites.

## Results

### Phylogenetic relationships between FoxP2, *FoxP2a* and *FoxP2b*

Homologous relationships between each pair of genes were sketched out in Figure 2. The 20 upstream and 20 downstream protein-coding genes flanking *FoxP2* shared a highly conserved synteny in four tetrapod species: *Homo sapiens*, *Taeniopygia guttata*, *Anolis carolinensis* and *Xenopus tropicalis* (Figure 2 and Information S2). In comparison, many co-localized genes around the *FoxP2a* and *FoxP2b* gene loci in *Danio rerio*, *Oryzias latipes*, *Oreochromis niloticus* and *Tetraodon nigroviridis* were orthologs of the one-to-two type. The 40 genes surrounding *FoxP2a* in *Oryzias latipes*, *Oreochromis niloticus* and *Tetraodon nigroviridis* resembled each other in synteny. Similarly, the co-localized genes flanking *FoxP2b* in *Oryzias latipes*, *Oreochromis niloticus* and *Tetraodon nigroviridis* shared a conserved synteny. In addition, *FoxP2a* in *Danio rerio* possessed a chromosomal location similar to that of *FoxP2a* in *Oryzias latipes*, *Oreochromis niloticus* and *Tetraodon nigroviridis*. However, a candidate ortholog of *FoxP2b* in these three teleosts could not be screened out in the genome of *Danio rerio*. In addition, *FoxP2a* and *FoxP2b* in *Oryzias latipes*, *Oreochromis niloticus* and *Tetraodon nigroviridis* resided in paralogous chromosome segments, i.e. paralogons (Figure 2). The linked paralogous gene pairs shared between *FoxP2a* and *FoxP2b* in *Oryzias latipes*, *Oreochromis niloticus* and *Tetraodon nigroviridis* were eight, six and nine, respectively. 

Multiple evolutionary changes (inversion, deletion and translocation) could be pinpointed in the chromosomal segments in which *FoxP2* was located during the long-term differentiation of these vertebrates. For instance, inversion of the chromosomal segment of *Taeniopygia guttata* occurred after its separation with the other three tetrapods. In comparison with chromosomal segments containing *FoxP2a* in *Danio rerio*, *Oryzias latipes* and *Tetraodon nigroviridis*, an inversion occurred in the corresponding location of *Oreochromis niloticus*. A microinversion of chromosomal segments containing *FoxP2a* occurred in *Oryzias latipes*, *Oreochromis niloticus* and *Tetraodon nigroviridis* relative to their tetrapod counterparts. The microinversion was also seen in chromosomal segments containing *FoxP2b* of *Oryzias latipes*, *Oreochromis niloticus* and *Tetraodon nigroviridis*.

Multiple sequence alignments showed that *FoxP2a* and particularly *FoxP2b* were both greatly divergent from their *FoxP2* orthologs. *FoxP2a* in teleosts possessed a 16-exon composition resembling that in human *FOXP2* (Figure 1A). Exon compositions of *FoxP2b* and *FoxP2a* in *Oryzias latipes* were similar to each other (Figure 1A). Specifically, *FoxP2a* of teleosts contained variable amino acid or fragment inserts (3–11), yet lacked a poly-Q repeat between site 154 and site 192 (Figure 1B and Information S3). All of the inserts were located in regions (i.e. exons 2, 6, 7, 11, 12 and 16) flanking three functional domains (zinc-finger, leucine-zipper and forkhead box). In contrast, these corresponding regions were likely truncated in *FoxP2b* of *Oryzias latipes* (Figure 1A).

All phylogenetic trees constructed with nucleotide or amino acid sequences in Data set 1 strongly supported a classification of *FoxP2*, *FoxP2a* and *FoxP2b* (Figure 3 and Information S4). *FoxP2a* of *Danio rerio*, *Ctenopharyngodon idella* and *Pygocentrus nattereri* were identified with a high degree of credibility. The gene trees reconstructed with the *FoxP2* and *FoxP2a* coding nucleotide sequences in Data set 2 (Information S4) were able to credibly reflect the phylogenetic relationships of the organisms involved [54] (Figure 4). The topological gene trees applied to subsequent tests in Data sets 1 and 2 (Figures 5 and 6) were revised ones conforming to the predicted species tree (Figure 4). 

### Functional divergence among FoxP2, FoxP2a and FoxP2b

The coefficient of functional divergence (θ_λ_) between Cluster 2 and Cluster 3 in Data set 1 was significantly greater than 0 (Table 1 and Figure 5). In spite of the statistical insignificance of θ_λ_ between Clusters 1 and 2 (or 3), the θ_λ_ between Clusters 1 and 3 was greater than that between Clusters 1 and 2. Moreover, a significant functional divergence between Clusters 1 and 2 was detected in Data set 2 (Table 1 and Figure 6), indicating that sites in regions flanking the functional domain (i.e. the forkhead box) have evolved at heterogeneous rates after the split of Cluster1 and Cluster 2 (Information S5). 

We plotted the posterior probability profiles of site-specific rate differences among clusters in Data set 1 and Data set 2 (Information S5). In Data set 1, the baseline posterior probability (i.e. posterior probability of the majority of sites) between Cluster 2 and Cluster 3 was the largest (0.683); the second largest was between Cluster 1 and Cluster 3 (0.565); the smallest was between Cluster 1 and Cluster 2 (0.260). Furthermore, there were 53, 2 and 0 amino acid sites with posterior probabilities above the cutoff value (0.70) when comparing Clusters 2 and 3, 1 and 3, and 1 and 2, respectively. The baseline posterior probability (0.384) when comparing Cluster 1 and Cluster 2 in Data set 2 was lower than 0.5, indicating no significant changes in the evolutionary rates of these sites occurred after the divergence of these two clusters. Ten amino acid sites with posterior probabilities greater than 0.7 were all located in exons (i.e. 3, 7, 11 and 17) surrounding functional domains, i.e. the zinc-finger, leucine-zipper and forkhead box.

### Structural divergence among FoxP2, FoxP2a and FoxP2b

Analyses on both data sets showed that hierarchical dendrograms, based on four kinds of pairwise distances (i.e. City-block, Correlation, Cosine and Euclidean), yielded highly consistent inconsistency coefficients and clusters (Information S6). All cophenetic correlation coefficients in these dendrograms were greater than 0.95, indicating that the trees faithfully represented original pairwise distances among sequences. 

According to the cutoff value (0.8) for inconsistency coefficients, the three clusters (Cluster 1, Cluster 2 and Cluster 3) were consistently separated from one another in the four hierarchical dendrograms from Data set 1 (Information S6), although the detailed classification was somewhat different. Specifically, FoxP2b of *Salmo salar* and *Gadus morhua* were singly classified in these dendrograms. FoxP2 of *Leucoraja erinacea* and *Lepisosteus oculatus* were grouped into Cluster 1, however FoxP2 of *Rana daunchina* was misclassified into Cluster 2 in the trees based on Correlation, Cosine and Euclidean pairwise distances. In addition, some links connecting dissimilar members (i.e. with inconsistency coefficients > 0.8) existed in these three clusters, indicating possible structural heterogeneity in the clusters. Notably, all dendrograms revealed that FoxP2a of Acanthopterygii were dissimilar to FoxP2a of *Salmo salar*, *Gadus morhua* and Otocephala. FoxP2a of *Takifugu rubripes* and *Tetraodon nigroviridis* showed significant dissimilarity from FoxP2a of other seven acanthopterygian fishes. Amphibian FoxP2 were possibly divergent from other tetrapod FoxP2. Subsequent stepwise discriminant analysis, which correctly classified high percentages of the original grouped cases (100%) and cross-validated grouped cases (97.2%), strongly demonstrated that FoxP2 of *Leucoraja erinacea*, *Lepisosteus oculatus* and *Rana daunchina* belonged to Cluster 1. Additionally, FoxP2b of *Salmo salar* and *Gadus morhua* were classified into Cluster 2 and Cluster 3, respectively. Moreover, FoxP2 in the ancestor of Osteichthyes and the ancestor of Actinopterygii, as well as all the ancestral FoxP2 in Sarcopterygii, were grouped into Cluster 1. FoxP2 in the ancestor of Teleostei, FoxP2b in the ancestor of Euteleostei and all the ancestral FoxP2a in Teleostei were classified as members of Cluster 2. All ancestral FoxP2b of Neoteleostei were classified into Cluster 3.

Similarly, analysis of structural divergence on Data set 2 produced consistent results (Information S6), classifying two clusters (Cluster 1 and Cluster 2). FoxP2 of *Leucoraja erinacea* and *Lepisosteus oculatus* were grouped into Cluster 1. The existence of a substantial inconsistency coefficient (> 0.8) in Cluster 2 indicated significant divergence between FoxP2a of Acanthopterygii and that of *Salmo salar*, *Gadus morhua* and Otocephala. FoxP2 of *Leucoraja erinacea* and *Lepisosteus oculatus* were confirmed to be members of Cluster 1 in subsequent stepwise discriminant analysis. In addition, FoxP2 in the ancestor of Osteichthyes and the ancestor of Actinopterygii were grouped together with all ancestral FoxP2 in Sarcopterygii into Cluster 1. All ancestral FoxP2a in Teleostei were classified into Cluster 2. Furthermore, 100% of the original grouped cases and cross-validated grouped cases were correctly classified.

### Variations of thermodynamic stability in FoxP2, FoxP2a and FoxP2b

**Figure 7:**
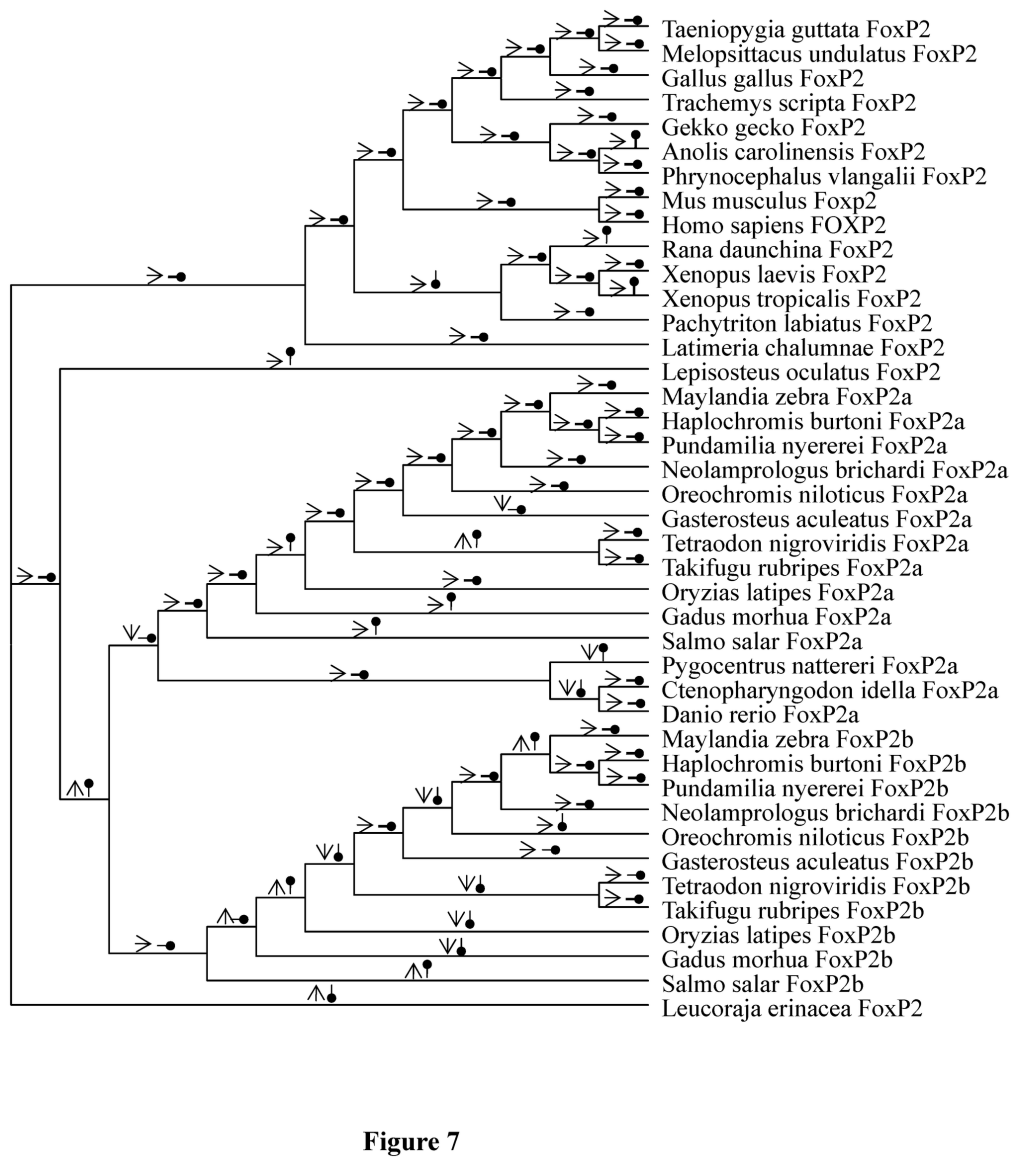
The thermodynamic stability changes of monomers (arrows with open arrowheads) and homodimers (arrows with circled arrowheads) based on average differences in total Gibbs free energy (ADTE). Rightward pointing arrowheads indicate insignificant thermodynamic stability changes. Upward and downward pointing arrowheads represent significant destabilizing and stabilizing thermodynamic stability changes, respectively.

The simulated ADTE variations of monomers and homodimers in different evolutionary scenarios showed that such ADTE variations were not always in unison (Figure 7). In 53 of 78 pieces of evolutionary trajectories, both monomers and homodimers underwent insignificant ADTE changes or maintained stable ADTE values. Different ADTE variations between monomers and homodimers occurred in more than half of the rest (i.e. 14/25). Moreover, dramatic divergence of ADTE fluctuations in the three clusters (i.e. Cluster 1, Cluster 2 and Cluster 3) indicated that the thermodynamic stability of the forkhead domain experienced divergent variations in these clusters. In Cluster 1, the numbers of significant ADTE changes in monomers and homodimers were 0 and 4, respectively. Likewise in Cluster 2, the numbers were 5 and 6; in Cluster 3, the numbers were 9 and 9. 

### Variation of natural selection pressures on *FoxP2*, *FoxP2a* and *FoxP2b*

The comparison of Model 3 to Model 0 in both data sets revealed variable natural selection pressures across sites (Information S7). Notwithstanding the fact that no positively selected sites were detected in comparison of Model 2 with Model 1, 521S was found using a more robust comparison between Model 8 and Model 7. In addition, comparison of the free ratios model to Model 0 revealed significant heterogeneity of the ω ratio in different branches.

Subsequent analysis of branch specific models in Data set 1 demonstrated that Cluster 3 experienced divergent natural selection pressures against both Cluster 1 and Cluster 2 (Information S7). Specifically, ω ratios of Cluster 1 and Cluster 3 in the independent two-ratio model were 0.022 and 0.139, respectively. Similarly, the ω ratio of Cluster 3 (0.139) was greater than that of Cluster 2 (0.030) in their two-ratio model. The insignificantly better fit of the independent two-ratio model (ω ratios: 0.022 in Cluster 1, 0.029 in Cluster 2) than the interdependent one-ratio model (ω ratio: 0.026 in Cluster 1 and Cluster 2) showed no differences of selection pressures in Cluster 1 and Cluster 2. However, a significant difference in selection pressures between Cluster 1 and Cluster 2 was demonstrated in Data set 2 (Information S7). The ω ratio of Cluster 1 (0.046) was slightly smaller than that of Cluster 2 (0.063) in the independent two-ratio model. 

Three foreground lineages (i.e. *FoxP2a* in ancestor of Tetraodontiformes, *FoxP2b* in ancestor Neoteleostei and *FoxP2b* in the ancestor of Tetraodontiformes) were found to have experienced accelerated evolution using Test 1 for branch-site models A in Data set 1 (Figure 5 and Information S7). Moreover, *FoxP2a* in the ancestor of Tetraodontiformes and *FoxP2b* in the ancestor of Neoteleostei probably underwent positive Darwinian selection. Most of the positively selected sites were situated surrounding the forkhead domain, specifically in exons 11, 12 and 15.

Likewise, five ancestral branches in Data set 2 (i.e. *FoxP2* in the ancestor of Actinopterygii, *FoxP2a* in the ancestor of Teleostei, *FoxP2a* in the ancestor of Cypriniformes, *FoxP2a* in the ancestor of Neoteleostei and *FoxP2a* in the ancestor of Tetraodontiformes) likely experienced accelerated evolution (Figure 6 and Information S7). Positive natural selection was detected in four ancestral branches, i.e. *FoxP2* in the ancestor of Actinopterygii, *FoxP2a* in the ancestor of Cypriniformes, *FoxP2a* in the ancestor of Neoteleostei and *FoxP2a* in the ancestor of Tetraodontiformes. The positively selected sites were mostly located in exons 7, 11, 15 and 17, flanking important functional domains (i.e. the zinc-finger, leucine-zipper and forkhead box). The test of selection pressures on 27 selected sequences in Data set 2, containing exons 6 and 16, produced similar results (not shown). These two exons with teleost-specific inserts were also demonstrated targets of positive natural selection and relaxed purifying selection.

## Discussion

### Duplication of FoxP2 in the 3R-WGD event

In this report, we identify the teleost-specific *FoxP2* duplication using synteny and paralogon analyses of related chromosomal segments. The subsequent phylogenetic reconstructions of *FoxP2*, *FoxP2a* and *FoxP2b* definitively confirm their homologous relationships. The orthologous one-to-two type relationships between *FoxP2* in tetrapods and *FoxP2a* and *FoxP2b* in teleosts are now established. The comparatively variable linearity of genes flanking *FoxP2a* and *FoxP2b* in teleosts probably results from multiple evolutionary changes (inversion, deletion and translocation) in these chromosomal segments after the tetrapod-teleost split. Although *FoxP2a* and *FoxP2b* show remarkable divergence in teleosts, the phylogenetic trees reconstructed with four member genes (i.e. *FoxP1*, *FoxP2*, *FoxP3* and *FoxP4*) of the *FoxP* subfamily clearly demonstrate that *FoxP2a* and *FoxP2b* in teleosts are members of *FoxP2* lineage (not shown). In comparison with *FoxP2*, greater divergence (e.g. exon-composition) of *FoxP2b* than *FoxP2a* suggests that *FoxP2b* evolves faster than *FoxP2a* after the gene duplication. Although dimerization of FoxP1, FoxP2 and FoxP4 has been demonstrated, which is important for their DNA binding and transcriptional activity [55], it is not clear whether FoxP2a and FoxP2b interact with one another in teleosts, leading to peculiar characteristics of exons flanking important functional domains in FoxP2a and FoxP2b.

The absence of *FoxP2b* in *Danio rerio* probably results from pseudogenization [26] of the redundant copy after the split of Otocephala and Euteleostei (Figure 1B). In addition to *FoxP2a* of *Danio rerio*, *FoxP2a* of two other species in Otocephala (*Ctenopharyngodon idella* and *Pygocentrus nattereri*) are phylogenetically determined. However, deletion of *FoxP2b* in *Ctenopharyngodon idella* and *Pygocentrus nattereri* is uncertain because their genomes are not available. The retention of *FoxP2b* in Euteleostei implies that the gene may conserve some functions of the parental *FoxP2* through subfunctionalization or may gain novel functions through neofunctionalization. The linked paralogon shared by *FoxP2a* and *FoxP2b* in teleosts indicates that these two duplicated genes were generated in a large-scale genome duplication event, i.e. 3R-WGD event [27,36] (Figure 2B). In addition, it should be noted that extra duplication of *FoxP2* may occur in polyploid vertebrates, such as *Xenopus laevis* and *Salmo salar* [56]. 

### Rapid evolution of *FoxP2a* and *FoxP2b* after the gene duplication

The significant functional divergence between FoxP2a and FoxP2b in Data set 1 implies that FoxP2a and FoxP2b probably execute divergent functions in teleosts. The θ_λ_ between Cluster 1 and Cluster 3 is greater than that between Cluster 1 and Cluster 2, suggesting that FoxP2b has evolved at a faster rate than FoxP2a after the gene duplication event. In addition, significant functional divergence of Cluster 1 and Cluster 2 in Data set 2 shows that the regions surrounding the functional domain (i.e. the forkhead box) rather than the functional domain itself in FoxP2a and FoxP2 are targets of divergent evolutionary rates. Therefore, the functions of *FoxP2a* may be more conservative than those of *FoxP2b* in teleosts.

Hierarchical cluster analysis and stepwise discriminant analysis of hydrophobicity profiles in Data sets 1 and 2 suggest that the structures of FoxP2, FoxP2a and FoxP2b are remarkably divergent from one another. In the stepwise discriminant analysis, FoxP2b of *Salmo salar* groups together with FoxP2a, as well as FoxP2 in the ancestor of Teleostei and FoxP2b in the ancestor of Euteleostei together with all ancestral FoxP2a in Teleostei, implying that structural divergence between FoxP2a and FoxP2b was probably very weak at the early stage of post-duplication. Perhaps the weak structural divergence of FoxP2a and FoxP2b generated functional redundancy of FoxP2b in Otocephala, further leading to the lineage-specific loss of FoxP2b. The significant structural divergence between FoxP2a and FoxP2b seems to coincide with the split of Neoteleostei from Euteleostei, implying more divergent functions between the genes of Neoteleostei than Salmoniformes. Additionally, some teleost lineages show significant FoxP2a structural divergence, likely indicating divergent functional adaptations in the lineages.

The significantly divergent variations of thermodynamic stability among FoxP2a, FoxP2b and FoxP2 clusters imply divergent functional adaptations of the forkhead domains in FoxP2a, FoxP2b and FoxP2. Intriguingly, the significant destabilizing effects of mutations (E519D and R523M) on both monomers and homodimers suggest that functional divergence of FoxP2 in teleosts possibly began in the pre-duplication phase. It has been demonstrated that the two mutations, as well as 521S, are responsible for the weak repressive activity of the medaka FoxP2a on the lung-specific gene of *CC10* [19]. In addition, predicted mutations in FoxP2b of the ancestor of Neoteleostei (S520A and S532N) and those (M518I, D522E and M523H) in FoxP2a of the ancestor of Tetraodontiformes may have led to significant destabilizing effects. Although the correlation between stability variation and molecular divergence is not clear, the simulated fluctuation of thermodynamic stability in each evolutionary scenario theoretically reveals a history of buffering and compensatory trade-offs in protein stability [57]. The theoretical history, therefore, suggest the nature of evolutionary changes in the forkhead box which have stability effects thereby aiding our investigation of the functional divergence of related mutations.

Our tests of natural selection pressures based on branch specific models strongly suggest the *FoxP2b* lineage has undergone a generally relaxed purifying selection pressure, which is significantly greater than that of the *FoxP2a* and *FoxP2* lineages. The regions flanking the functional domain of the forkhead box, rather than the forkhead domain per se, in the *FoxP2a* lineage has experienced a milder purifying selection pressure than those in the *FoxP2* lineage. Thus the asymmetrical natural selection pressures acting on the *FoxP2a*, *FoxP2b* and *FoxP2* clusters, particularly between *FoxP2a* and *FoxP2b*, likely led to asymmetrical functional divergence among these clusters. Furthermore, natural selection pressures on branches and sites in each cluster show significant heterogeneity, especially positive natural selection acting on several ancestral branches. Notably, positive selection pressure mainly acts on regions flanking functional domains. Therefore, positive selection pressure possibly was the driving force in forming the teleost-specific inserts in FoxP2a and the truncated regions in FoxP2b. It is also notable that *FoxP2* in the ancestor of Actinopterygii probably underwent positive selection, suggesting that positive selection played a role in variation during the pre-duplication phase [58]. The potential pseudogenization and subsequent loss of *FoxP2b* in Otocephala possibly resulted from excessively relaxed purifying selection on the redundant copy in the fixation phase [26,58]. Positive Darwinian selection acts on *FoxP2a* and *FoxP2b* after Neoteleostei branched off from Euteleostei, implying adaptive evolution of these two genes coincided with diversification of Neoteleostei. 

### Insights into the functional adaptations of *FoxP2a* and *FoxP2b*

Studies of FoxP2 in both animal models and humans have shown that the pleiotropic gene is widely involved in a series of important physiological activities and developmental processes [1,4,17]. In addition to the neural control of vocalization, it has been demonstrated that Foxp2 is a crucial regulator in *Mus musculus* for lung and esophageal development, especially for postnatal lung alveolarization [12,22,59]. These findings indicate that *FoxP2* is critically involved in the development of foregut derived pulmonary organ systems through regulating lung-specific genes, e.g. *CC10* and surfactant protein C (SPC).

The teleost swim bladder has been considered homologous to the lung on the basis of consistency of developmental blastema and molecular pathways [28,29]. It has been suggested that the surfactant systems essential for air filled organs are also homologous in gas bladders and lungs [60]. However, the functions of the swim bladder and lung are markedly different from each other. The swim bladder has evolved to be an organ mainly controlling buoyancy regulation not oxygen respiration [29]. Studies of *FoxP2a* in teleosts have provided only limited information concerning its functions for organ development [18,19,21]. Fishes share a conservative expression pattern of *FoxP2a* in development of CNS with other vertebrates. No *FoxP2a* expression has been detected in the swim bladder of adult *Danio rerio* [29], whereas the area from which the swim bladder is derived shows high *FoxP2a* expression in early (7 days post-fertilization) embryonic development in *Maylandia zebra* [20]. Expression of *FoxP2a* decreases to a light but visible level in the swim bladder of late larval (20 days post-fertilization) embryos of *Mchenga conophorus* [20] and possibly disappears before adulthood, as is the case in *Danio rerio* [29]. 

Given the essential functions of *FoxP2* in foregut derived air-breathing organ, rapid evolution of *FoxP2* (*FoxP2a* and *FoxP2b*) in teleosts is possibly correlated with adaptive evolution of the swim bladder [19]. Notably *Lepisosteus oculatus* retains a swim bladder to breathe air and possesses *FoxP2* resembling that of tetrapods. The three species of Otocephala (*Danio rerio*, *Ctenopharyngodon idella* and *Pygocentrus nattereri*) and one species of Protacanthopterygii (*Salmo salar*) are physostomous fishes with a pneumatic duct connecting the swim bladder to the gastrointestine. It has been suggested that the Otocephala-specific loss of *FoxP2b* possibly results from excessively relaxed purifying selection on the functionally redundant gene. In spite of retention of *FoxP2b* in *Salmo salar*, the divergence of *FoxP2a* and *FoxP2b* is noticeably weak. Positive natural selection probably acts on *FoxP2a* and *FoxP2b* to favor the divergence of the genes in Neoteleostei, which are physoclistous fishes (i.e. swim bladders of these fishes have lost a pneumatic duct). Taken together, the present study provides a useful introduction to studying the functional involvement of *FoxP2a* and *FoxP2b* in teleosts.

## Supporting Information

Information S1 **Species coverage, PCR primers and predicted FoxP2 sequences.** (PDF)

Information S2 **Protein-coding genes around *FoxP2*, *FoxP2a* or *FoxP2b* in eight species.** Twenty upstream (with negative order number) and 20 downstream (with positive order number) protein-coding genes.
(PDF)

Information S3 **Amino acid or fragment inserts in FoxP2 and FoxP2a of ray-finned fishes.** (PDF)

information S4 **Phylogenetic reconstructions based on Data sets 1 and 2.** (PDF)

Information S5 **Posterior probability profiles of site-specific rate difference among clusters in Data sets 1 and 2.** (PDF)

Information S6 **Hierarchical dendrograms of genes in Data sets 1 and 2.** (PDF)

Information S7 **Test of natural selection pressures on Data sets 1 and 2.** (PDF)
