# Supplementary material for: Rapid Diversification of FoxP2 in Teleosts through Gene Duplication in the Teleost-Specific Whole Genome Duplication Event
Source: PLoS One. 2013 Dec 9;8(12):e83858. doi: 10.1371/journal.pone.0083858 (PMC3857310; doi:10.1371/journal.pone.0083858)
Supplement: Information S1 — Species coverage, PCR primers and predicted FoxP2 sequences. (PDF) [file pone.0083858.s001.pdf]

S1.1 Species coverage.

English name	Latin name	Accession numbers of *FoxP2* sequences in GenBank or Ensembl
elephant shark	*Callorhynchus milii*	See S1.4.1
little skate	*Leucoraja erinacea*	See S1.4.2
spotted gar	*Lepisosteus oculatus*	BK008612
red-bellied piranha	*Pygocentrus nattereri*	AB302880.1
zebrafish	*Danio rerio*	DQ061052.1
grass carp	*Ctenopharyngodon idella*	FJ415224.1
Atlantic salmon	*Salmo salar*	See S1.4.3
rainbow trout	*Oncorhynchus mykiss*	CA387093.1
Atlantic cod	*Gadus morhua*	See S1.4.4, GW852741.1
medaka	*Oryzias latipes*	EU143691.1, XM_004069881.1
three-spined stickleback	*Gasterosteus aculeatus*	See S1.4.5
fugu	*Takifugu rubripes*	BK008614, ENSTRUT00000041785 (Ensembl)
green spotted puffer	*Tetraodon nigroviridis*	BK008615, ENSTNIT00000011486 (Ensembl)
Nile tilapia	*Oreochromis niloticus*	BK008617, ENSONIG00000008874 (Ensembl)
dwarf gourami	*Colisa lalia*	JX543516
cichlid fish	*Neolamprologus brichardi*	BK008618, See S1.4.6
cichlid fish	*Pundamilia nyererei*	BK008619, See S1.4.7
African cichlid fish	*Haplochromis burtoni*	BK008620, See S1.4.8
cichlid fish	*Maylandia zebra*	BK008621, See S1.4.9
west Indian Ocean coelacanth	*Latimeria chalumnae*	BK008622
spotless stout newt	*Pachytriton labiatus*	JX543512
African clawed frog	*Xenopus laevis*	BC170268.1
western clawed frog	*Xenopus tropicalis*	BC135996.1
Emei music frog	*Rana daunchina*	JX543513
red-eared slider turtle	*Trachemys scripta*	EU076411.1
green anole	*Anolis carolinensis*	BK008623
Qinghai toad-headed lizard	*Phrynocephalus vlangalii*	JX543514
tokay gecko	*Gekko gecko*	JX543515
zebra finch	*Taeniopygia guttata*	AY395709.1
budgerigar	*Melopsittacus undulatus*	AY466101.1
chicken	*Gallus gallus*	XM_001232372.1
mouse	*Mus musculus*	NM_053242.4
human	*Homo sapiens*	NM_014491.3

S1.2 PCR primers for *FoxP2*.

Experimental animals	PCR primers (Forward / Reverse)
dwarf gourami (*Colisa lalia*)	1. AATGGAATGAGCACCCTAA / GCGTTGCGTCTGAAGT<br>2. TGCCCCTGTCGTCAGGTGGT / GGTGCCTCCTAGTCACTCCAGG<br>3. ACGCAGATGTCAGACCGCCA / GTGCCTCCTAGTCACTCCAGG
spotless stout newt (*Pachytriton labiatus*)	1. TGCAGCAACTTGGAAGAATG / TTGTTACTGGGTCCCAAAGG<br>2. TCAAGTAGTGACACGAGCAG / GGTGGTGGAGGTAGTAGAAG<br>3. ATGACTCCCCAGGTGATC / AGGCTTAGGTTGTGACGC
Emei music frog (*Rana daunchina*)	1. TGCAGGAATCTGCAACAGAG / GGACTGGAAGAGCAGATTGG<br>2. TTATGGAAAGAAGTTACTGG / GAGGTTTTGGAGATGGC<br>3. CAGGCAATGATGACCCAC / GGTCAGTGCTATGTCCCTTC<br>4. GACACCGGCACTGAAGTAAGCACA / GGTAGTAGAAGAGGAATTGTTAGT
Qinghai toad-headed lizard (*Phrynocephalus vlangalii*)	1. CAGGCAACGATGACCCA / CTACTCGAACGAAACACTTGTG<br>2. TCTTCTACTACCTCCTCTACC / TTTGTGACCTTCGCTTC<br>3. TGAGCACTCTAAGCAGCCAA / AGCCCTCCATGTTTAATGCC<br>4. CGAACGTCTTCAAGCAATGA / TGCATTCTTCCAAGTTGCTG<br>5. ATGATGCAGGAATCTGCG / GCTGTAACATAACCGCTTG<br>6. ACACGGACATTTGCCTACT / AATGAGTTCCTTTAGGGTTG<br>7. CAGCGTCAGGGACTCATTTC / TCATGGGATGAGCTGTCTCG
tokay gecko (*Gekko gecko*)	1. ATGGCATTAAACATGGAGGG / ACTGCCTGTCACTCGATTCC<br>2. TGAGCACTCTAAGCAGCCAA / AGCCCTCCATGTTTAATGCC<br>3. TCTTCTACTACCTCCTCTACC / TTTGTGACCTTCGCTTC<br>4. CAGCGTCAGGGACTCATTTC / TCATGGGATGAGCTGTCTCG<br>5. ACACGGACATTTGCCTACT / AATGAGTTCCTTTAGGGTTG

S1.3 Predicted *FoxP2* sequences.

Animals	Genome data	Gene	Location
elephant shark (*Callorhynchus milii*)	Eshark 1.4X assembly in Elephant Shark Genome Project	***FoxP2***	AAVX01053315.1, AAVX01097261.1, AAVX01074296.1, AAVX01074296.1, AAVX01146517.1, AAVX01074297.1, AAVX01334789.1, AAVX01024995.1, AAVX01328199.1, AAVX01223556.1, AAVX01223556.1, AAVX01274185.1
little skate (*Leucoraja erinacea*)	GCA_000238235.1 in National Center for Biotechnology Information	***FoxP2***	CONTIG_71454, CONTIG_44624, CONTIG_1027758, CONTIG_54013, CONTIG_1618495, CONTIG_150870, CONTIG_54919, CONTIG_2669517, CONTIG_35267, CONTIG_39535, CONTIG_1027869, CONTIG_42166
spotted gar (*Lepisosteus oculatus*)	GCA_000242695.1 in National Center for Biotechnology Information	***FoxP2***	contig021086
Atlantic salmon (*Salmo salar*)	GCA_000233375.1 in National Center for Biotechnology Information	***FoxP2a***	Contig_100926, Contig_092217, Contig_139950, Contig_142218, Contig_202713, Contig_090982, Contig_047897, Contig_078738,
		FoxP2b	Contig_003053
rainbow trout (*Oncorhynchus mykiss*)	National Center for Biotechnology Information	***FoxP2***	CA387093.1
Atlantic cod (*Gadus morhua*)	GCA_000231765.1 National Center for Biotechnology Information	***FoxP2a***	contig434189, contig498925, contig105365, contig105364, contig105362, contig105361, contig176495, contig180772, contig180770, contig180769, contig176044, contig180768, contig180767, contig180765
three-spined stickleback (*Gasterosteus aculeatus*)	Broad Institute v1.0 in USCS Genome Bioinformatics website	***FoxP2a***	chrIV:19043821-19098325
		FoxP2b	chrXIX:4482904-4496355
fugu (*Takifugu rubripes*)	v5.0 (Oct. 2011) in USCS Genome Bioinformatics website	***FoxP2a***	chr18:6697675-6741202
green spotted puffer (*Tetraodon nigroviridis*)	V8 assembly (March 2007) in USCS Genome Bioinformatics website	***FoxP2a***	chr19:4790161-4832993
Nile tilapia (*Oreochromis niloticus*)	GCA_000188235.2 in National Center for Biotechnology Information	***FoxP2a***	contig024819
cichlid fish (*Neolamprologus brichardi*)	GCA_000239395.1 in National Center for Biotechnology Information	***FoxP2a***	contig002864, contig002865, contig002866, contig002867
		FoxP2b	contig007451

cichlid fish (*Pundamilia nyererei*)	GCA_000239375.1 in National Center for Biotechnology Information	***FoxP2a***	contig007021
		FoxP2b	contig011593
African cichlid fish (*Haplochromis burtoni*)	GCA_000239415.1 in National Center for Biotechnology Information	***FoxP2a***	contig002935, contig002934
		FoxP2b	contig015614
cichlid fish (*Maylandia zebra*)	GCA_000238955.2 in National Center for Biotechnology Information	***FoxP2a***	contig030874, contig030875
		FoxP2b	contig036976
west Indian Ocean coelacanth (*Latimeria chalumnae*)	GCA_000225785.1 in National Center for Biotechnology Information	***FoxP2***	contig125653, contig125652, contig164425, contig125647, contig125662
green anole (*Anolis carolinensis*)	Broad version AnoCar2.0 in USCS Genome Bioinformatics website	***FoxP2***	chr5:59299609-59499579

S1.4 Sequences unsubmitted to public databases.

S1.4.1 Elephant shark (*Callorhynchus milii*)

Partial *FoxP2* CDS (The partial exon2, partial exon11, exon12, exon13 and exon17 could not be predicted through genome screenings.):

ATAAGCAACAGTTCAATGAATCAAAATGGAATGAGCACTCTAAGCAGCCAATTAGATGCTGGCAGCAGAGATGGAA
GATCAAGTGGTGACACCAGTGGTGAAGTAAGCACAGTAGAACTGCTGCATCTGCAACAACAGCAGGCACTACAAG
CAGCAAGACAGCTTCTTCTTCAGCAGCAAACCAGTGGTCTGAAATCTCCAAAGGGAAACGACAAACAGCGAACAC
TGCAGGTGCCTGTGTCAGTGGCCATGATGACTCCCCAGGTGATCACCCCTCAGCAGATGCAGCAGATCCTCCAGCA
GCAAGTGTTGTCCCCACAGCAGCTTCAGGCTCTCCTTCAGCAGCAGCAAGCAGTCATGTTACAGCAGCAACAGCTT
CAGGAATTCTATAAAAAACAGCAGGAACAGTTACATCTTCAGCTTCTGCAACAGCAGCAACAACAACATCAACAGC
AGCAGCAGCAACAACAACAACAACAGCAGCAGCAACAACAACAACAACAGCAACAACAGCAGCAGCAACAGCA
GCAACACACAGGCAAACAATCAAAAGAGCAGCAGTTGGCAGCCCAACAGATTGTCTTCCAACAGCAGCTTCTTCA
AATGCAACAAATCCAGCAGCAGCAGCATTTGATGAACTTACAACGTCAGGGACTCATCACTATCCAGCCAGGACAG
CCGTCCCTTTCCGTCCAGTCCCTGCCACAAGGTTTAAGCCCCGCAGAGATTCAGCAGCTCTGGAAAGAAGTGACCG
GAGTGCACAATGTGGAAGACAGCGTCCTGAAGCATGGCGGGCTGGACCTCTCTACCAATAATACTACTTCTACTACC
TCCACTACCACTTCCAAAGCATCGCCTCCGATATCTCATCATTCCTTAGTGAACGGACAGGCTTCAATCCTCACAACA
AGAAGAGATAGCTCATCACATGAGGAGACTGGGGCCACTCATCCTCTGTACGGCCACGGCGTGTGCAAATGGCCTG
GATGTGAAAGTATTTGTGAAGATTTTGGACAGTTTTTAAAACACCTGAGTAATGAACACGCTCTGGATGATCGCAGC
ACAGCTCAGTGCCGGGTACAGATGCAAGTGGTTCAACAGCTGGAGATTCAGCTTTCTAAAGAACGTGAGCGTCTTC
AGGCAATGATGACCCACCTTCACATGAGACCCTCGGATCCCAAGCCATGTCCGAAACCTGTGGGACCAATACGAAG
AAGACACAGTGATAAGTACAACATTCCCATTTCATCAAATGCAGTACGCCATAATCTTAGTCTGCACAAATGTTTTGT
TCGAGTAGAAAATGTTAAAGGGGCAGTGTGGACAGTGGACGAAATAGAATACCAAAAGCGAAGGTCACAAAAGAT
AACAGGAAGCCCGACGTTAGTCAAAAACATACCTACTAGCCTGGGGTATGGAGCAGCTCTCAATGCAAGCTTGCAG
GCTGCACTTGCGGAGAGCAGTTTACCTTTGCTTAGTAACCCAAGCCTGATAAACACCACCTCCAGTGGCTTACTGCA
GGCAACCCATGAAGACCTCAACGGTTCATTGGATCACCTCGACAGCAATGGGAATACCAGCCCAGGATGTTCTCCG
CAGCAACAT

S1.4.2 Little skate (*Leucoraja erinacea*)

Partial *FoxP2* CDS (The exon5 could not be predicted through genome screenings.):

ATGATGCAAGAATCCACGACAGAGACAATAAGCAACAGTTCAATGAATCAAAATGGAATGAGCACTCTAAGCAGCC
AATTAGATGCTGGCAGCAGAGATGGAAGATCAAGTGGTGACACTAGTACGGAAGTAAGCACAGTCGAACTGCTGCA
TCTGCAACAACAGCAGGCACTACAAGCAGCAAGACAGCTTCTTCTTCAGCAGCAAAGCAGTGGTCTGAAATCTCC
AAAGGGAAACGACAAGCAGCGAGCATTGCAGGTGCCTGTCTCGGTGGCCATGATGACTCCCCAGGTGATCACTCCT
CAGCAGATGCAGCAGATCCTTCAGCAGCAAGTGCTGTCCCCTCAGCAGCTCCAGGCTCTCCTCCAACAGCAGCAAG
CAGTAATGTTACAGCAGCAGCAGTTGGCAGCCCAGCAGATAGTCTTCCAACAGCAGCTACTCCAAATGCAACAAAT
CCAGCAGCAACAGCATTTGATGAACCTACAACGTCAGGGACTCATTACCATCCAGCCTGGACAGCCGACCCTTTCA
GTCCAATCTCTGCCACAAGGCTTAAGCCCTGCGGAGATTCAGCAGCTCTGGAAAGAAGTGACCGGGGTGCACAACA
TGGAAGACGGCATTTTGAAGCATGGCGGGCTGGATCTCTCTGCCAATAATTTGTCCTCTACTACCTCCACTACCACTT
CCAAAGCATCGCCACCAATAACTCACCATTCATTAATGAATGGACAGACTTCAGTCCTCACCACAAGAAGAGACAG
CTCTTCACATGAGGAAACTGGGGCCAGTCATCCTCTCTATGGCCATGGTGTGTGTAAATGGCCTGGATGTGAAAGCA
TTTGCGAAGATTTCGGACAGTTTTTGAAGCACCTTAATAACGAGCATGCATTAGATGACAGGAGTACTGCACAGTGT
CGGGTCCAAATGCAAGTTGTGCAGCAGTTGGAAATCCAGCTTTCTAAAGAACGTGAACGTCTTCAGGCAATGATGA
CCCACCTCCATATGAGACCTTCTGATCCTAAGCCATGTCCGAAACCTCTGAATCTGGTGTCGAGTGTCACAATGTCTA

AGAACATTTCAGAGACATCCCCACAGAGTTTACCTCAGACTCCCACCACACCAACAGCCCCAGTCACCCCTGTCAC
GCAAGGACCCTCCGTAATCACTCCAGCCAACGTACACAATGTGGGACCAATTCGAAGAAGACATGGTGATAAGTAC
AACATTCCCATTTCATCAGAGATTGCCCCAAACTATGAATTTTATAAGAATGCAGATGTCAGACCTCCATTTACTTATG
CAACTCTTATAAGGCAGGCTATCCTTGAATCATCTGACAGGCAGTTAACACTTAATGAAATCTACAATTGGTTTACAC
GAACATTTGCCTATTTCAGGCGTAACGCAGCAACTTGGAAGAATGCTGTACGCCATAACCTTAGTCTGCACAAGTGT
TTTGTTCGAGTAGAAAATGTTAAAGGGGCAGTGTGGACAGTGGACGAAATAGAATACCAAAAGAGAAGGTCACAA
AAGATAACAGGAAGCCCAACATTAGTCAAAAATATACCGACGAGTCTTGGGTATGGAGCAGCACTCAATGCCAGCT
TGCAGGCTGCACTTGCAGAGAGCAGTTTACCTTTACTTAGTAACCCAAACCTGATAAACACCACCTCCAGTGGCTTA
CTGCTGGCAGCCCATGAAGACCTCAATGGTTCGCTGGATCACCTCGACACCAATGGGAATATTAGCAGAGGATGTTC
TCCACAGCAACATATTCAACCCATTCATGTCAAGGAAGAACCAATGAATGCAGAAGAAGATGAGGGCCCAATGTCC
TTGGTAACGACAGCCGATCACAGTCCAGAGATCGAGGACAGAGATATTGAAGAGGAACCTTCATCTGAAGATCTAG
AATAA

S1.4.3 Atlantic salmon (*Salmo salar*)

Partial *FoxP2a* CDS (The partial exon8 and whole exon16 could not be predicted through genome screenings.):

ATGATGCAGGAGTCGGCCACAGAGACAATAAGCAACAGTTCAATGAGTCAAAATGGAATGAGCAGCCTGAGCAGC
CTATGCAGCCAATTAGATGCTGGCAGTAGAGATGGAAGATCAAGCGGTGACACGAGCAGCGAAGTAAGCACAGTCG
AGCTGCTGCATCTCCAACAACAGCAGGCCCTACAAGCAGCGAGACAATTACTCTTACAACAGCCAGGCAGTGGCCT
GAAATCTCCAAAGAACAACGACAAACAGCGTCCACTGCAGGTGCCGGTGTCGGTGGCCATGATGAGTCCCCAGGT
GATCACCCCCCAGCAGATGCAGCAGATCCTCCAGCAGCAGGTCCTATCCCCTCAGCAGCTCCAGGCCCTGCTCCAA
CAGCAGCAGGCTGTCATGCTGCAGCAGCAACACCTGCAGGAGTTTTATAAGAAACAGCAGGAGCAGCTGCACCTG
CAGCTTTTACAACAGCAGCACCCTGGAAAGCAAGCAAAAGAGCAACAGCAGCAGCAGCAGCATCAGCAGCAGCT
CGCCGCCCAGCAGCTCGTCTTCCAGCAGCAACTCCTCCAGATGCAACAGCTCCAGCAGCAGCAGCACCTGCTCAAC
ATGCAGCGCCAGGGCCTGCTCTCCCTGCCCCCGGCCCCAGGACAGGCAGCCCTCCCCGGGCAGACCATGCCACCAG
CTGGACTGAGCCCAGCAGAGCTCCAACAGTTGTGGAAGGACGTGACCGGCGGCGGCCACAACATGGAGGACAAC
GGCATCAAACACAGCGTCGTCGGCGGCGGCCTGGACCTGACCACTACCAACTCTTCCTCGACTACCTCCTCTACCCC
TTCCAAAGCATCACCTCCCATCTCTCACCACTCCATCGCCAACGGCCAGTCTCCCGCGCTCAATTCCAGAAGAGAGA
GAGAGCGAGAGAGGGAACGGGAAAGGGAGAGGTGCAAGTGGCCCGGCTGCGAGAGCGTCTGTGAGGACTTCGGA
CAGTTTTTGAAGCACCTTAACAATGAGCATGCTCTAGACGACAGGAGCACGGCCCAGTGTCGAGTCCAGATGCAGG
TGGTACAGCAGCTAGAGATACAGCTTTCTAAAGAACGTGAGCATCTTCAGGCGATGATGGCCCACTTGCACATGCGG
CCCTCGGAGCCCAAGCCATCTCCAAAACCTCTGAACTTGGTATCGAGCGTCACCATGTCGAAGAACCTGCCGTCGG
TGTCCCCTCCGAACGTACCTCAGACCCCCACCACGCCCAGCGCCCCCGTGACGCCCATGTCCCAGGTGCCCCAGGT
GCCCTCCCTGCTCAGCGCCGCCAACGTCCCAAGCATGGGCGCCATGCGCAGACGTCACTCCGACAAGTACTCCATG
CAACTGTCTTCAGGTGGTGACACAGTATTTATTTTTCCAGAGATCGCCCCAAACTATGAGTTTTATAAGAATGCCGAT
GTCAGACCGCCATTTACTTATGCAACCCTCATAAGGCAGGGTATCATGGAGTCGGGCGACATGCAGTTAACGCTTAAT
GAAATCTACAGCTGGTTCACGCGCACATTCGCTTACTTCAGACGCAACGCAGCTACTTGGAAGAACGCCGTTCGCC
ACAACCTCAGTCTGCACAAGTGCTTTGTGCGTGTGGAGAACGTGAAGGGGGCCGTGTGGACGGTGGATGAGATGG
AGTACCAGAAACGCAGGTCTCAGAAGATAACGGGAAGCCCGACGCTTGTGAAGAACCTTCCCTCCAGCCTGGGCTA
TGGAGCTGCTTTAAACGCCAGCTTACAGATCCACGTCAAGGAGGAGCCGTTGAACATGGACGACGATGACTGTCCG
ATGTCGCTGGTGACGACAGCCAACCACAGCCCAGAGCTAGACGACGACCGGGAGCTGGAGGAGGGCAACCTATCA
GATGACCTGGAGTGA

***FoxP2b* CDS** (It was predicted with GENSCAN method.):

ATGAGATGGAGTCTACAACCCACACAAGTGGCTCAGGTAGTGCAGCTCATCCAGGATGGCACATCAATGCGAGCTG
TGGCAAGAAGGTTTGCTGTGTCTGTCAGCGTAGTGTCCAGAGCATGGAGGCGCTACCAGGAGACAGGCCAGTACAT

CAAGAGACGTGGAGGAGGCCGTAGGAGGGCAACAACCCAGCAGCAGGACCGCTACCTCCGCCTTCACACTAAATG
TCATTTGAGTTCTGGGTCCAGTGTCACCATGTCCAAGAGCCTGCCACCACTGTCCCCTCAAGATGACCCTCAGATGT
CCCCAACGGCCCCTGTCACTTCCCCATCCTCACCCCGGGGGCCTGCAGGACTCTGCCCCACAAGTGTGGGGGCAAT
GCGAAGACACCACTCAGACAAGCACACCTCTCTTTGCTCTCTGTCTTCAGAATTTAGCCCAAATCATGAGATGTACA
GAAATGCAGATATCAGGCCACCTTTTACCTATGCTACTCTGATAAGACAGGCTATAATGGATGCATCAGACATGCAGT
TAACACTTAATGAGATATACAGCTGGTTTACACAGACCTTTGCCTACTTCAGACGCAATGCTGCAGTGTGGAAGAAT
GCAGTCCGCCACAACCTGAGCCTGCACAAGTGTTTTGTGCGTGTGGAAAATGTGAAGGGGGCCGTGTGGACAGTG
GACGAGGTGGAGTACCAGAGAAGGAGGTCTCAGAAGATCACAAGAAGCCCAACGCTAGGGAAGAACCTTCTCTCC
AGCCTTGCTTATTGGACTGCTCTTAATACCAGTTTACAAGCTCCACTGACTGAGAATGCTCTGACTGTATTAAAGAAC
AGGACCCCTGCAGACCTGATAGGCAGCAGTTATTTGGGTCTGGTACACCGGACCAAGTTACTAAAACATCCGGACC
TCCAGGACACAAATGGACAACGCAGCCCACACTCCACTTCACTGGCCCAGCTGTGTACTCACATAAAACATCTCCA
ATCCAAAGTTAAATCTAGAATTGGCTTCATATTTCGCAACAAAGCATCCTTCACTCATGCTGCCAAACATACCCTCAT
AAAACTGACCATCTTACCGATCCTCGACTTTGGCGATGTCATTTACAAAATAGCCTCCAACACCCTACTCAACAAATT
GGATGCAGTCTATCACAGTGCCATCCGTTTTGTCACCAAAGCACCATATACTACCCACCACTGCGACCTGTATGCTCT
CGTTGGCTGGCCCTCGCTTCATACTCGTCGCCAAACCCACTGGCTCCATGTCATCTACAAGACCCTGCTAGGGATGG
AGCCTATGACATTCACAGTAGGAAATATCACTCCAAGCCATCTTCGAAAGCTGACAGCCCTGCACCCACATCAAACC
ACACCCCCAAGGCATCTCTTGTTTGCCTTCAATCATGGCCGCTAG

S1.4.4 Atlantic cod (*Gadus morhua*)

Partial *FoxP2a* CDS (The exon6 could not be predicted through genome screenings.):

ATGATGCAGGAGCCGGCCACAGAGACAATAAGCAACAGTTCAATGAGTCAAAATGGAGTGAGCAGCCTGAGCGGC
AGCCAATTAGAAGCGGGCTGCCGGGATGGGAGATCAAGTGGTGGTGACACGGGCGGCAGTGAAGTAAGCACAGTC
GAGCTGCTGCATCTGCAACAACAGCAGGCTCTACAGGCAGCGAGGCAATTACTACTACAGCAGCCAGGCAGCGGCC
TGAAGTCTCCAAAGAATCACGACAAGCATCGTCCGCCACAGGTGCCAGTGTCGGTGGCCATGATGAGTCCCCAGGT
GATCACTCCCCAGCAGATGCAGCAGATCCTGCAGCAGCAGGTGCTCTCCCCCCAGCAGCTGCAGGCGCTGCTCCAG
CAGCAGCAGGCAGTCATGTTGCAGCAGCAACATCTGCAGGAGTTCTACAAGAAACAACAAGAGCAACTTCACCTG
CAGCTTCTCCAACAGCAGCACCCTGGCAAGCAGGTCAAAGAGGCCGGTCTGAGCCCCGCTGAGCTCCAGCAGCTT
TGGAAGGACGTGACCGGGGGTGGCGGCCACGGCATGGACGACAGCGGCATGAAACATGGCGGCAACGGCGGCGG
CGTGATGAATAGCGGCGCCGGAGGCCTGGACCTCAGCACCAACCACAACAACAACAGCAACAACAACTCCTCCTT
GACTACCTCCTCGTGCAACCCTTCCAAAGCATCCCCACCCCTCTCCCACCACCACGCCATCGCCAACGGACAGTCG
CCCGCCCTCAACCACAGAAGAGAACGAGAGCGGGAACGGGAACGGGAACGGGAGCGCGAGCGCGAGAGCTCGTT
ACATGAAGAGACGTTAGGATCCCACTCCCTGTACGGCCACGGCGTCTGCAAGTGGCCCGGGTGCGAGAGCATCTGC
GAGGACTTCGGACAGTTCGTCAAGCACCTGAACAACGAGCACGCCCTTGATGACCGGAGCACAGCTCAGTGTCGG
GTCCAGATGCAGGTCGTACAGCAGCTGGAAATACAGCTTTCTAAAGAACGGGAGCGTCTGCAGGCGATGATGGCCC
ACTTGCACATGAGGCCTACGGAGCCCAAGTCATCTCCCAAACCACTCAACCTGGTGTCCAGCGTCACCATGTCCAA
GAACCTCCCGTCGACGTCCCCCCCTACCCTACCTCAGGCCGCCACCACGCCCACGGCGCCCGCCCCCCCCATGCCC
CCCGTCCCCCAGGTGCCCTCCATGCTGGGGGGGGCCAACGTGCCCAGCATGGGCGCCATGCGCCGTCGCCACGCCG
ACAAGTACTCCATGTCGCTGTCGTCAGGTGGTGACACAGTATTTATTTTTCCAGAGATCGCCCCAAACTACGAGTTTT
ATAAGAACGCAGATGTCAGGCCACCGTTCACTTATGCAACCCTCATAAGACAGGCGATCATGGACTCCAACGACATG
CAGTTAACGCTCAATGAAATCTACAGCTGGTTCACGCGCACGTTCGCCTACTTCAGACGCAACGCGGCCACCTGGA
AGAATGCCGTGCGCCACAACCTCAGCCTGCACAAGTGCTTTGTGCGCGTGGAGAACGTGAAAGGCGCCGTGTGGA
CGGTGGACGAGATGGAGTACCAGAAACGCCGGTCACAGAAGATCACAGGGAGCCCCACTCTTGTGAAAAACCTGC
CGTCCAGTCTGGGCTACGGCGCGGCCCTCAACGCCAGCCTACAGGCCGCCCTGGCCGAGACCTCCATGCCCCTTCT
GGGCACGCCGGGCCTGATGAACAGCAGCTCCACGGGGCTCATGGGCGGCTCGCCCCACTGCCTGATGGGAGGGTC

CCCGCACGGCCTGATGGGTGGCAGCCCGCCCGGCCTGATGGGGTGCAGCCCCCCGGGCATGATGATGGGCGGTAGC
CCCCCCGGGATGATGCTGGGCGGCGGCGGCGTCGGCGGCATGGGGAGCAACAGCCCCCTGGGACGGATGAGTGGC
AGCCCCCCCAGCCTGCTGCAGTCGAGCCACGAGGACCTGAACGGCGTGCTGGACCACCTGGATGCCAACGGCCAC
GGCAGCCCCGGGTACTCCCCCCAGGGTCACATGCCCCAGATCCACGTCAAGGAGGAGCCAATCAACATGGACGAC
GACGACTGCCCCATGTCACTGGTCACGACGGCCAATCACAGTCCAGAGCTGGACGAGGACCGGGAGCTGGAGGAA
GGGAACCTCTCAGAGGACCTGGAGTGA

S1.4.5 Three-spined stickleback (*Gasterosteus aculeatus*)

Partial *FoxP2a* CDS (The exon6 and exon16 could not be predicted through genome screenings.):

ATGATGCAGGAGTCGGCCACAGAGACAATAAGCAACAGTTCAATGAGTCAAAATGGAATGAGCACCCTAAGCAGC
AGCCAATTAGAGGCTGGCAGTAGAGATGGGAGATCAAGCGCTGGTGACACGAGCAGCGAAGTAAGCACAGTCGAG
CTGCTGCATCTGCAACAACAGCAGGCCCTACAAGCAGCGAGGCAATTACTCTTGCAGCAACCAGGCAGTGGCCTGA
AGTCTCCAAAGAGCCACGACAAGCACCGTCCACTGCAGGTTCCAGTGTCAGTGGCCATGATGAGTCCCCAGGTGAT
CACGCCGCAACAGATGCAGCAGATCCTCCAGCAGCAGGTGCTCTCCCCCCAGCAGCTCCAGGCCCTGCTCCAACAG
CAGCAGGCCGTGATGCTGCAGCAGCAACACCTGCAGGAGTTTTACAAGAAACAACAGGAGCAGCTTCATCTGCAG
CTCCTCCAGCAGCAGCACCCTGGCAAGCAGGCTAAAGAGCCAGCTGGCCTGAGCCCTGCAGAGCTCCAGCAGTTG
TGGAAGGATGTGACCGGAGGCGGGAGCCACACCATGGAGGACAACGGCATCAAACACAGCAACAGTGGCAACGG
TGGCAACGGCGGCAACGGCGGCGGCGGAGGTGGCGGCGGCGGTTTGGACCTCACCACCAACAACTCCTCCTCAAC
TACCTCCTCCTCTAATCCCGCCAAAGCGTCGCCGCCCATCTCCCACCACTCCATTGCAAACGGACAGTCCCCAGCCC
TCAACCACAGGAGAGAGAGGGAACGGGAGCGCGAGCGGGAAAGGGAGCGAGAGAGTTCACTACATGAAGAAAG
TGGAGGCACCCACCCTCTGTACGGCCACGGTGTGTGTAAGTGGCCCGGCTGCGAGAACATCTGCGAGGACTTCGGA
CAGTTTTTGAAGCATTTAAACAGCGAACATGCCCTCGATGATCGGAGCACAGCGCAGTGTAGAGTCCAAATGCAAG
TCGTACAGCAGCTGGAAATACAACTTTCTAAAGAACGTGAGCGTCTTCAGGCGATGATGGCCCACTTGCACATGCG
GCCCTCGGAACCCAAGTCATCTCCCAAACCACTCAACTTGGTGTCCAGCGTCACCATGTCCAAGAACTTGCCCTCG
GCATCGCCCCCAAACTTACCTCAGACACCCACCACGCCCACGGCACCCATCACACCCATGGCGGCCATGCCACAGG
TGCCGTCGGTACTGGGAGGAGCCAACGTCCCCAGCATGGGAGCCATGCGCAGACGCCACTCTGACAAGTACTCCAT
GCCTCTGTCGTCAGGTGGTGACACAGTATTTATTTTTCCAGAGATTGCCCCAAACTACGAGTTTTATAAAAACGCAGA
TGTCAGACCGCCATTTACTTATGCAACCCTCATAAGACAGGCTATCATGGACTCTGCCGACATGCAGCTAACGCTTAA
CGAAATCTACAGCTGGTTCACGCGCACGTTCGCCTACTTCAGACGCAACGCTGCAACTTGGAAGAACGCCGTTCGC
CACAACCTCAGTCTGCACAAGTGCTTTGTGCGTGTGGAGAACGTGAAGGGGGCGGTGTGGACGGTAGACGAGATG
GAGTACCAGAAACGCAGGTCGCAGAAGATCACAGGAAGCCCGTCACTTGTCAAGAACCTGCCCTCCAGTCTTGGCT
ACGGAACTGCACTAAACGCCAGCTTACAGCCTCCCATTCACGTGAAGGAGGAGCCACTTAACATGGATGAAGATGA
CTGTCCAATGTCCCTGGTGACGACAGCCAATCACAGTCCAGAGCTGGACGACGACCGGGAGCTGGAGGAAGGGAA
CTTATCAGAGGACCTGGAGTGA

***FoxP2b* CDS** (It was predicted with GENSCAN method.):

ATGCCCCATGCAGGGACAGAGAAGGTAGACGCACAACGCCATCACATCTGCCAGCCGCACTTCACACTGCGTGACT
CTGCCTGCTGCTCCGAATCCACGCATCAGCTTCACGCCCGTCAAACGCCGGCCAGCAGCCTTCTCAGTCACGCCGA
CGCGGCTGCAGGAGGGAGAGTAGCCGATGGGGAGAACTGGTCGTATCTTCACCATAAGCAGGTTTTTCTGGCTATGA
TGCAGCAGCTGCTGTCCCCCGACCAGCTGCAGGCTGTGATCCAGCAGAAGCAACAAGCTCTTCTCCTGCAGCAGCA
ACATCTGAAAGAGTTTTACAAGAAGCAACAACAACAGATTCATCTGCAGCTGCTTCAACAAAGGTCCTGCAAGAAA
GCCAAAGAGCTCCCTGCGCAGCAGCTCGTCTTCCAGCAGCTCCTCCGGCTGCAGCAGCAACAGCAGCAGCTCCTCC
GGCTGCAGAGACCTCGGCTGCCCCCCCCGGCCCTCGCTCCAGCTGGTTTCGGCTCTGCAGAGACGTGGAAAGAGCT
CACGACTGAAGACAAAGACGCACCGAAGAGCCATCGGGAGCCTTCCGGTCCCGACCTGGGGAGGCCGAGCGGAG
ACCAGGGGTCGCCGTCTCCTCGCCGGGCGACGCGGCACATGAGCGGCGAGCACACCCTGGATGACAGAAGCACGG

CGCAGTGCCGAGTCCAGACGCAGGTGGTCCAGCAGCTCGGGATGCAGCTCGGCAAAGAACGGGAGCGTCTGCGGG
CGATGATGGATCACCTGCACCTGCCACCTCTGGAGTCTCAGTCGATCTCTGCACCTCTGCAGCCGCCACAATCAGAA
CCGGCCAGTCTTCAGCTCGGCTCCGTCCTGCCCCCCCCGCGTCCATCGGACTCGGCCCGGGTGTCCCCGTCCCCGGT
GTGCGGAGGACAGTCCCCCGGTCACAGGGCCGCACAACGACGTCGCCATCACCCTTTGGTCTACTCGCTGTCCTCA
GAGGATGAACACGAGCTTTACAAGACCACCGACATCAGACCACCTTTCACCTACGCGACCCTCATCAGACAGGCAA
TAATGGAAACATCCGACCTGCAACTAACACTCAACGAGATCTACAACTGGTTCACACGGACGTTTGCTTATTTCAGA
CGCAACGCGGCCACTTGGAAGAACGCGGTGCGTCACAACCTGAGCCTGCACAAGTGTTTTGTGCGCGTGGAGAAC
GTGAAAGGCGCCGTGTGGACGGTGGACGAGGTGGAGTACCAGAGGAGGAGATCCCAGAGGGTCACCGGGAGTCC
GACGCTGATGAAGAACGTCTCCTCCAACCTGGACTTGGGGACCATCCGGCCGAGCGGCAGCCGACAGCGACTAGA
GGAGCTGGAGCCCCTCGAGCCACTTTCTTCTTCTTGTTTGCAGACGTCGGTACCGGGAGGACCGGAGAGCGCTGAT
GGAAGTCAAATACAGGAGAGCAACGCGACGAGCAGCCGCAGGAAAGCAAACTTCTCCCCAGCTCTCTGGCCGCAC
TGCCCCGTGCGGCTCCTGGGCCCGATGGAGGCCTCGGGGCCCTCTGCTGCTGGAGGCGCCGGCGGAGGAGCACGA
CTGCCACTCGGTGCTGCTGCCCACCGGGTGTCTGGAGTGCTGCGACGGCAGCTAGAAGGTGTGGACGATGACGTTA
AAGCCAACGTGGAGCTCATTGGCACCGCAGAGACATTTCTTTTGACGAAGCATCTTATTGTGAAAGTCGTGGACAA
CAAAAACAGCTGCTGA

S1.4.6 Cichlid fish (*Neolamprologus brichardi*)

***FoxP2b* CDS** (It was predicted with GENSCAN method.):

ATGCCAGAGTCCCCTCTCAGTCCCACAACAGCCCGTCAAAACCCAGCCAGCAGCCTCCTCAGTCACACAGACAGTA
GTGGAGGGGAAAGAGTTGCTAATGGGAACGCTGGCGCTCTGAGTGGCGACGACTGGCAGACTCTTCAGCATAAAC
AGGTCTTTCTTGCTATGATGGCCCCTCAGCAGATTCAGCAGTTTCTGGCTCCCAGCCAGCTGCAGGCTCTGATCCAC
CAGAAGCAGCAAGCCCTTCTGCTTCAGCAGCATCACCTGAAAGAGTTCTACAAGAAGCAGCAACAACAGATTCAG
CTGCTTCAGCAACAGTCCAGTAAGAAAATCAAAGAGCTCGTCTTCCAGCAGCTCATCCAACTCCAGCAGCAGCAAC
AGCAGCAGCTCCTCCGGGCGCACAGACCAGCGCTGTCCTCTCCCACCCTCTCTCCAGCTTGTCTCAGCCCCGCAGA
GATGCAGCAAGTGTGGAAAGAGCTCACAATTGGAATGACTGAAGATAAAACCACAATAAAAGAGAATCGAGAATCT
TCCGCTGGCAAAATAATGTCAGCAAAAGTCCCAAGGAGACAAGCCGATGACCTGCAGTCTGCCTGTCCTCACAGAG
CTGAAAGCACTGTTAAAGGTGATCACGCTGCCAAACACGCCCTCTTCGGTCACGGCGTGTGTAATTGGCCCGGTTG
TGAGTCCGTCTGTGAAAACTTCAGCCAGTTTATCAAGCACATAAACAGTGAACATACTCTGGATGACAGAAGTACA
GCCCAGTGCAGAGTCCAGATGCAGGTGGTTCAGCAGCTTGAGCTTCAGCTCTGCAAAGAACGGCAGCGTCTGCAG
GCAATGATGGCTCACCTGCATCTGCCATCTTTAGAAGCTCAATCGCTCTCGGCGCCTGCACAGTCGCCACAGTCCGA
CACGGCTGCTGACCCGTGCGGCCCGCAGCTCACTTCAGTCACCAACAGCTTGCCCTCGCTGAGACCATCAGACCCA
GCCCAGGTGTCCCCTCGACCTCTAGATCCTGTTGGCACTCCATCCCAGGGGTGTGAGAAAGAGTTCCCATCTCACAC
GTCAGGCGTTGGGCCTATGAGACGTCGTAATCACCCTCTGGTCTACTCGCTGTCTTCAGAAAATGAATATGAGCTTTA
CAAAAACACTGATCTCAGACCACCTTTCACCTATGCCACACTGATTCGACAGGCTATCATGGAAGCATCAGACATGC
AACTAACCCTCAACGAGATATACAACTGGTTCACAAGGACATTTGCTTATTTCCGACGCAACGCCGCCACTTGGAAG
AATGCAGTTCGACACAACTTGAGCCTGCACAAGTGTTTCGTGCGCGTGGAGAATGTGAAAGGTGCAGTGTGGACTG
TGGATGAGGTGGAATACCAGAGGAGGCGATCCCAGAAGATCACAGGAAATCCATCACTGATGAAAAGTATTTCCTC
CAGTGCAGGTTATGGAACCCTAATGAATTCTGGCTTGCAGACCGCACTGGCAGAGGCGTCACTGCCAGGACTCAAG
AAAGACAACGTAAGAGGAAATTCAAAGAGTCAAATACAAAAGAGCAGCATGGCTGACAGCCACAGAATCCAAAAC
TTCTCTCCTCGTGTCCAGACGCCGCTTTTCCTTGAAGACAAAGAGCTGAAGGTGAATGACCAAGGATGTCTGATAC
AGACAGTGAAACCAGTCAGTTTGCAGCCAGACATGAGTGAAAATGATGGAAGGCATTTGTTTGACCTTGAATGA

S1.4.7 Cichlid fish (*Pundamilia nyererei*)

***FoxP2b* CDS** (It was predicted with GENSCAN method.):

ATGCCAGAGTCCCCTCTCAGTCCCACAACAGCCCGTCAAAACCCAGCCAGCAGCCTCCTCAGTCACACAGACAGTA
GTGGAGGGGAAAGAGTCGCTAATGGGAACGCTGGCGCTCTGAGTGGCGACGACTGGCAGACTCTTCAGCATAAAC
AGGTCTTTCTTGCTATGATGGCCCCTCAGCAGATTCAGCAGTTTCTGGCTCCCAGCCAGCTGCAGGCTCTGATCCAC
CAGAAGCAGCAAGCCCTTCTGCTTCATCAGCATCACCTGAAAGAGTTCTACAAGAAGCAGCAACAACAGATTCAGC
TGCTTCAGCAACAGTCCAGTAAGAAAATCAAAGAGCTCGTCTTCCAGCAGCTCATCCAACTCCAGCAGCAGCAACA
GCAGCAGCTCCTCCGGGCGCACAGACCAGCGCTGTCCTCTCCCGCTCTCTCTCCAGCTTGTCTCAGCCCTGCAAAG
ATGCAGCAAGTATGGAAAGAGCTCACAATTGGAATGACTGAAGATAAAACCACAATAAAAGAGAATCGAGAATCTT
CCGCTGGCAAAATAATGTCAGCAAAAGTCCCAGGGAGACAAGCCGATGACCAGCAGTCTGCCTGTCCTCACAGAG
CTGAGAGGCACATAAGCAGTGAACACACTCTGGATGACAGAAGTACAGCCCAGTGCAGAGTCCAGATGCAGGTGG
TTCAGCAGCTTGACCTTCAGCTCTGCAAAGAACGGCAGCGTCTGCAGGGAATGATGGCTCACCTGCATCTGCCATCT
TTAGAAGCTCAATCGCTCTCGGCGCCTGCACAGTCGCCACGGTCCGACACGGCTGCTGACCCGTGCGGCCCGCAGC
TCACTTCAGTCACCAACAGCTTGCCCTCGCTGAGACCATCAGACCCAGCCCAGGTGTCCCCTCGACCTCTAGATCCT
GTTGGCACTCCATCCCAGGGGTGTGAGAAAGAGTTCCCCTCTCACACGTCAGGCGTTGGGCCAATCAGACGTCGTA
ATCACCCTCTGGTCTACTCGCTGTCTTCAGAAAATGAATATGAGCTTTACAAAAACACCGATCTTAGACCACCTTTCA
CCTATGCCACACTGATTCGACAGGCTATCATGGAAACATCAGACATGCAACTAACCCTCAACGAGATATACAACTGG
TTCACAAGGACATTTGCTTATTTCCGACGCAACGCCGCCACTTGGAAGAATGCGGTTCGACACAACTTGAGCCTGC
ACAAGTGTTTCGTGCGCGTGGAGAATGTGAAAGGTGCGGTGTGGACTGTGGATGAGGTGGAATACCAGAGGAGGC
GATCCCAGAAGATCACAGGGTACAGTAGATCCTCCTTGTTTATCGCAGTGCCGCCATCAGGAAATCCATCACTGATG
AAAAGTATTTCCTCCAGTGCAGGTTATGGAACCCTAATGAATTCTGGCTTGCAGACCGCACTGGCAGAGGCGTCACT
GCCAGGACTCAAGAAAGACAACGTAAGAGGAAATTCAAAGAGTCAAATACAAAGGAGCAGCATGGCTGACAGCC
ACAGAAACCAAAACTTCTCTCCTCGTGTCCAGACGCCGCTTTTCCTTGAAGACAAAGAGCTGAAGGTGAATGACCA
AGGATGTCTGATACAGACAGTGAAACCAGTCAGTCTGCAGCCAGACATGAGTGAAAATGATGGAGGGCATTTGTTT
GACCTTGAATGA

S1.4.8 African cichlid fish (*Haplochromis burtoni*)

***FoxP2b* CDS** (It was predicted with GENSCAN method.):

CACATAAGCAGTGAACACACTCTGGATGACAGAAGTACAGCCCAGTGCAGAGTCCAGATGCAGGTGGTTCAGCAG
CTTGACCTTCAGCTCTGCAAAGAACGGCAGCGTCTGCAGGCAATGATGGCTCACCTGCATCTGCCATCTTTAGAAGC
TCAATCGCTCTCGGCGCCTGCACAGTCGCCACAGTCCGACACGGCTGCTGACCCGTGCGGCCCGCAGCTCACTTCA
GTCACCAACAGCTTGCCCTCGCTGAGACCATCAGACCCAGCCCAGGTGTCCCCTCGACCTCTAGATCCTGTTGGCA
CTCCATCCCAGGGGTGTGAGAAAGAGTTCCCCTCTCACACGTCAGGCGTTGGGCCAATCAGACGTCGTAATCACCC
TCTGGTCTACTCGCTGTCTTCAGAAAATGAATATGAGCTTTACAAAAACACTGATCTCAGACCACCTTTCACCTATGC
CACACTGATTCGACAGGCTATCATGGAAACATCAGACATGCAACTAACCCTCAACGAGATATACAACTGGTTCACAA
GGACATTTGCTTATTTCCGACGCAACGCCGCCACTTGGAAGAATGCGGTTCGACACAACTTGAGCCTGCACAAGTG
TTTCGTGCGCGTGGAGAATGTGAAAGGTGCGGTGTGGACTGTGGATGAGGTGGAATACCAGAGGAGGCGATCCCA
GAAGATCACAGGGTACAGTAGATCCTCCTTGTTTATCGCAGTGCCGCCATCAGGAAATCCATCACTGATGAAAAGTA
TTTCCTCCAGTGCAGGTTATGGAACCCTAATGAATTCTGGCTTGCAGACCGCACTGGCAGAGGCGTCACTGCCAGG
ACTCAAGAAAGACAACGTAAGAGGAAATTCAACGAGTGAAATACAAAGGAGCAGCATGGCTGACAGCCACAGAA
ACCAAAACTTCTCTCCTCGTGTCCAGACGCCGCTTTTCCTTGAAGTCAAAGAGCTGAAGGTGAATGACCAAGGATG
TCTGATACAGACAGTGAAACCAGTCAGTCTGCAGCCAGACATGAGTGAAAATGATGGAGGGCGTTTGTTTGACCTT
GAATGA

S1.4.9 Cichlid fish (*Maylandia zebra*)

***FoxP2b* CDS** (It was predicted with GENSCAN method.):

ATGGCCCCTCAGCAGATTCAGCAGTTTCTGGCTCCCAGCCAGCTGCAGGCTCTGATCCACCAGAAGCAGCAAGCCC
TTCTGCTTCATCAGCATCACCTGAAAGAGTTCTACAAGAAGCAGCAACAACAGATTCAGCTGCTTCAGCAACAGTC
CAGTAAGAAAATCAAAGAGCTCGTCTTCCAGCAGCTCATCCAACTCCAGCAGCAGCAACAGCAGCAGCTCCTCCG
GGCGCACAGACCAGCGCTGTCCTCTCCCGCTCTCTCTCCAGCTTGTCTCAGCCCTGCAAAGATGCAGCAAGTATGG
AAAGAGCTCACAATTGGAATGACTGAAGATAAAACCACAATAAAAGAGAATCGAGAATCTTCCGCTGGCAAAATAA
TGTCTGCAAAAGTCCCAGGGAGACAAGCCGATGACCAGCAGTCTGCCTGTCCTCACAGAGCTGAGAGGCACATAA
GCAGTGAACACACTCTGGATGACAGAAGTACAGCCCAGTGCAGAGTCCAGATGCAGGTGGTTCAGCAGCTTGACC
TTCAGCTCTGCAAAGAACGGCAGCGTCTGCAGGCAATGATGGCTCACCTGCATCTGCCATCTTTAGAAGCTCAATCG
CTCTCGGCGCCTGCACAGTCGCCACAGTCCGACACGGCTGCTGACCCGTGCGGCCCGCAGCTCACTTCAGTCACCA
ACAGCTTGCCCTCGCTGAGACCATCAGACCCAGCCCAGGTGTCCCCTCGACCTCTAGATCCTGTTGGCACTCCATCC
CAGGGGTGTGAGAAAGAGTTCCCCTCTCACACGTCAGGCGTTGGGCCAATCAGACGTCGTAATCACCCTCTGGTCT
ACTCGCTGTCTTCAGAAAATGAATATGAGCTTTACAAAAACACTGATCTCAGACCACCTTTCACCTATGCCACACTG
ATTCGACAGGCTATCATGGAAACATCAGACATGCAACTAACCCTCAACGAGATATACAACTGGTTCACAAGGACATT
TGCTTATTTCCGACGCAACGCCGCCACTTGGAAGAATGCGGTTCGACACAACTTGAGCCTGCACAAGTGTTTCGTG
CGCGTGGAGAATGTGAAAGGTGCGGTGTGGACTGTGGATGAGGTGGAATACCAGAGGAGGCGATCCCAGAAGATC
ACAGGAAATCCATCACTGATGAAAAGTATTTCCTCCAGTGCAGGTTATGGAACCCTAATGAATTCTGGCTTGCAGAC
CGCACTGGCAGAGGCGTCACTGCCAGGACTCAAGAAAGACAACGTAAGAGGAAATTCAAAGAGTGAAATACAAA
GGAGCAGCATGGCTGACAGCCACAGAAACCAAAACTTCTCTCCTCGTGTCCAGACGCCGCTTTTCCTTGAAGACAA
AGAGCTGAAGGTGAATGACCAAGGATGTCTGATACAGACAGTGAAACCAGTCAGCCTGCAGCCAGACATGAGTGA
AAATGATGGAGGGCATTTGTTTGACCTTGAATGA
