# Supplementary material for: Rapid Diversification of FoxP2 in Teleosts through Gene Duplication in the Teleost-Specific Whole Genome Duplication Event
Source: PLoS One. 2013 Dec 9;8(12):e83858. doi: 10.1371/journal.pone.0083858 (PMC3857310; doi:10.1371/journal.pone.0083858)
Supplement: Information S2 — Protein-coding genes around FoxP2, FoxP2a or FoxP2b in eight species. Twenty upstream (with negative order number) and 20 downstream (with positive order number) protein-coding genes. (PDF) [file pone.0083858.s002.pdf]

Protein-coding genes around *FoxP2*, *FoxP2a* or *FoxP2b* in eight species. 20 upstream (with negative order number) and 20 downstream (with positive order number) protein-coding genes are listed as follows.

Chr.7 of *Homo sapiens* (*H.s.*):
DLD[-20], LAMB1[-19], LAMB4[-18], NRCAM[-17], PNPLA8[-16], THAP5[-15], DNAJB9[-14], C7orf66[-13], IMMP2L[-12], LRRN3[-11], DOCK4[-10], ZNF277[-9], IFRD1[-8], C7orf53[-7], TMEM168[-6], C7orf60[-5], GPR85[-4], AC073346.2[-3], AC073348.1[-2], PPP1R3A[-1], FOXP2[0], MDFIC[1], TFEC[2], TES[3], CAV2[4], CAV1[5], MET[6], CAPZA2[7], ST7[8], WNT2[9], ASZ1[10], CFTR[11], CTTNBP2[12], NAA38[13], ANKRD7[14], KCND2[15], TSPAN12[16], ING3[17], CPED1[18], WNT16[19], FAM3C[20]

Chr.1A of *Taeniopygia guttata* (*T.g.*):
ING3[-20], TSPAN12[-19], ENSTGUG00000004681[-18], ENSTGUG00000004691[-17], ENSTGUG00000004698[-16], ENSTGUG00000004730[-15], CTTNBP2[-14], CFTR[-13], ASZ1[-12], WNT2[-11], ST7[-10], ENSTGUG00000005077[-9], MET[-8], CAV1[-7], CAV2[-6], ENSTGUG00000005269[-5], TES[-4], TFEC[-3], ENSTGUG00000005310[-2], MDFIC[-1], FoxP2[0], PPP1R3A[1], C7orf60[2], TMEM168[3], C7orf53[4], IFRD1[5], ZNF277[6], DOCK4[7], IMMP2L[8], LRRN3[9], DNAJB9[10], THAP5[11], AVPR2[12], PNPLA8[13], NRCAM[14], ENSTGUG00000005721[15], PDZRN4[16], GXYLT1[17], YAF2[18], ZCRB1[19], PPHLN1[20]

Chr.5 of *Anolis carolinensis* (*A.c.*):
ADAMTS20[-20], PRICKLE1[-19], PPHLN1[-18], ZCRB1[-17], YAF2[-16], GXYLT1[-15], PDZRN4[-14], CNTN1[-13], NARCAM[-12], PNPLA8[-11], ENSACAG00000025984[-10], IMMP2L[-9], LRRN3[-8], DOCK4[-7], ZNF277[-6], IFRD1[-5], TMEM168[-4], C7orf60[-3], GPR85[-2], PPP1R3A[-1], FoxP2[0], MDFIC[1], TFEC[2], PSMB6[3], TES[4], ENSACAG00000022838[5], CAV2[6], CAV1[7], MET[8], CAPZA2[9], ST7[10], ASZ1[11], CFTR[12], CTTNBP2[13], ENSACAG00000007208[14], KCDN2[15], ENSACAG00000024705[16], TSPAN12[17], FKBP6[18], ING3[19], CPED1[20]

Scaffold GL172672.1 of *Xenopus tropicalis* (*X.t.*):
prickle1[-20], pphln1[-19], zcrb1[-18], yaf2[-17], gxylt1[-16], pdzrn4[-15], cntn1[-14], nrcam[-13], pnpla8[-12], avpr2.2[-11], thap5[-10], dnajb9[-9], LRRN3[-8], dock4[-7], znf277[-6], ifrd1[-5], tmem168[-4], c7orf60[-3], gpr85[-2], PPP1R3A[-1], FoxP2[0], mdfic[1]

Chr.4 of *Danio rerio* (*D.r.*):
mapk11[-20], mapk12b[-19], ENSDARG00000045835[-18], ENSDARG00000045834[-17], vegfab[-16], mrps18a[-15], rsph9[-14], TMEM63A[-13], brn1.2[-12], ENSDARG00000075956[-11], ccnc[-10], ENSDARG00000054321[-9], pgm3[-8], CU571169.1[-7], foxm1[-6], RHNO1[-5], pex26[-4], CU571169.4[-3], CU571169.3[-2], ENSDARG00000074223[-1], FoxP2a[0], PPP1R3A (2 of 2)[1], gpr85[2], tmem168a[3], ifrd1[4], dock4b[5], cald1l[6], tnnt2e[7], lta4h[8], elk3[9], BX537336.1[10], PHYH (2 of 2)[11], phyh[12], ucmaa[13], mcm10[14], nudt5[15], cdc123[16], camk1da[17], ccdc3[18], optn[19], ENSDARG00000074547[20]

Chr.23 of *Oryzias latipes* (*O.l.*):

BRAF[-20], LARGE[-19], ENSORLG00000009922[-18], STRAP[-17], TMPO (2 of 2)[-16], SLC25A3[-15], LDHB (1 of 2)[-14], ENSORLG00000009997[-13], ENSORLG00000010060[-12], NRCAM (2 of 2)[-11], PNPLA8 (2 of 2)[-10], THAP5[-9], DNAJB9[-8], ENSORLG00000010199[-7], ENSORLG00000010337[-6], ENSORLG00000010353[-5], ENSORLG00000010441[-4], TFEC[-3], ENSORLG00000010465[-2], ENSORLG00000010468[-1], FoxP2a[0], PPP1R3A (2 of 2)[1], A4KUT7_ORYLA[2], TMEM168 (2 of 2)[3], IFRD1[4], DOCK4[5], ENSORLG00000010605[6], LRRN3[7], CALD1 (1 of 2)[8], ENSORLG00000010664[9], DNM1L (1 of 2)[10], DHX57[11], FAM40B[12], AHCYL2 (1 of 2)[13], TMEM209[14], PRDM4[15], ASCL4[16], ENSORLG00000011013[17], SLC37A3[18], DENND2A (2 of 2)[19], CDPF1[20]

Chr.6 of *Oryzias latipes* (*O.l.*):
WDR78[-20], ENSORLG00000001737[-19], CAT[-18], SYT12[-17], ENSORLG00000001774[-16], HMG20A[-15], LINGO1 (1 of 2)[-14], CSPG4[-13], A4KUR1_ORYLA[-12], SNUPN[-11], PTPN9[-10], SIN3A (1 of 2)[-9], CRABP1 (1 of 2)[-8], WDR61[-7], SLC25A44 (2 of 2)[-6], IREB2[-5], SNX1 (1 of 2)[-4], ENSORLG00000001974[-3], AP3B2[-2], ENSORLG00000002037[-1], FoxP2b[0], PPP1R3A (1 of 2)[1], C7orf60[2], TMEM168 (1 of 2)[3], ENSORLG00000002070[4], PTDSS2[5], CDKN1C[6], ENSORLG00000002107[7], ENSORLG00000002113[8], AVPR1A (1 of 2)[9], PPM1H (1 of 2)[10], MON2[11], ENSORLG00000002318[12], ENSORLG00000002374[13], FBLN1 (1 of 2)[14], WNT7B (1 of 2)[15], C4B6D9_ORYLA[16], YARS2[17], ENSORLG00000002430[18], MRPS35[19], TEAD4[20]

Chr.LG17 of *Oreochromis niloticus* (*O.n.*):
ENSONIG00000021152[-20], PPARA (2 of 2)[-19], CDPF1[-18], DENND2A (2 of 2)[-17], SLC37A3[-16], ENSONIG00000016725[-15], PRDM4[-14], TMEM209[-13], AHCYL2 (1 of 2)[-12], FAM40B[-11], MORN2[-10], DHX57[-9], DNM1L (1 of 2) [-8], ENSONIG00000016761[-7], CALD1 (1 of 2)[-6], LRRN3 (1 of 2)[-5], DOCK4[-4], IFRD1[-3], TMEM168 (2 of 2)[-2], GPR85[-1], FoxP2a[0], ENSONIG00000016801[1], TFEC[2], ENSONIG00000016805[3], ENSONIG00000016816[4], DNAJB9 (2 of 2)[5], THAP5[6], PNPLA8 (2 of 2)[7], NRCAM (2 of 2)[8], ENSONIG00000016833[9], PDZRN4 (2 of 2)[10], GXYLT1 (2 of 2)[11], YAF2 (2 of 2)[12], ZCRB1[13], PPHLN1 (1 of 2)[14], PRICKLE1 (2 of 2)[15], PUS7L[16], ENSONIG00000016850[17], RAB19 (2 of 2)[18], LRRC17[19], CCDC146[20]

Chr.LG13 of *Oreochromis niloticus* (*O.n.*):
WDR78 (2 of 2)[-20], SRPK2[-19], ADCK2[-18], DENND2A (1 of 2)[-17], TEAD4[-16], MRPS35[-15], ENSONIG00000008828[-14], CDKN1B[-13], YARS2[-12], PPARA (1 of 2)[-11], TRIM35 (49 of 54)[-10], WNT7B (1 of 2)[-9], FBLN1[-8], ENSONIG00000008845[-7], ENSONIG00000008850[-6], ENSONIG00000008854[-5], MON2[-4], PPM1H (1 of 2)[-3], AVPR1A (2 of 2)[-2], ANKRD30B[-1], FoxP2b[0], C7orf60[1], TMEM168 (1 of 2)[2], PTDSS2[3], CDKN1C[4], ENSONIG00000008890[5], IFITM5[6], ENSONIG00000008894[7], ENSONIG00000008896[8], ENSONIG00000008898[9], ENSONIG00000008899[10], ENSONIG00000008900[11]

Chr.19 of *Tetraodon nigroviridis* (*T.n.*):
RAB19 (2 of 2)[-20], ENSTNIG00000018499[-19], PUS7L[-18], PRICKLE1 (2 of 2)[-17], PPHLN1 (2 of 2)[-16], ENSTNIG00000018503[-15], ZCRB1[-14], YAF2 (2 of 2)[-13], GXYLT1 (2 of 2)[-12], PDZRN4 (2 of 2)[-11], ENSTNIG00000018508[-10], NRCAM (2 of 2)[-9], PNPLA8 (2 of 2)[-8], HMGXB4[-7], ANKRD54[-6], DNAJB9[-5], ENSTNIG00000018515[-4], ENSTNIG00000018516[-3], FOXRED2[-2],

TFEC[-1], FoxP2a[0], PPP1R3A (2 of 2)[1], GPR85[2], TMEM168 (2 of 2)[3], IFRD1[4], DOCK4[5], LRRN3[6], CALD1 (2 of 2)[7], DNM1L (1 of 2)[8], DHX57[9], MORN2[10], FAM40B (2 of 2)[11], AHCYL2 (1 of 5)[12], TMEM209[13], PRDM4 (1 of 2)[14], SLC37A3[15], DENND2A (2 of 2)[16], CDPF1[17], PPARA (2 of 2)[18], WNT7B (2 of 2)[19], ATXN10[20]

Chr.13 of *Tetraodon nigroviridis* (*T.n.*):
ENSTNIG00000008448[-20], SNX1 (1 of 3)[-19], IREB2[-18], SLC25A44[-17], WDR61[-16], CRABP1 (1 of 2)[-15], SIN3A (1 of 2)[-14], PTPN9[-13], SNUPN[-12], SNX33[-11], CSPG4[-10], LINGO1 (1 of 2)[-9], HMG20A[-8], ENSTNIG00000008461[-7], ENSTNIG00000008462[-6], CDKN1C[-5], PTDSS2[-4], TMEM168 (1 of 2)[-3], C7orf60[-2], PPP1R3A (1 of 2)[-1], FoxP2b[0], ANKRD30B[1], AVPR1A (1 of 2)[2], PPM1H (1 of 2)[3], MON2[4], ENSTNIG00000014265[5], AKR1D1[6], ENSTNIG00000014266[7], FBLN1 (1 of 2)[8], WNT7B (1 of 2)[9], PPARA (1 of 2)[10], YARS2[11], CDKN1B[12], ENSTNIG00000003412[13], MRPS35[14], TEAD4[15], DENND2A (1 of 2)[16], ADCK2[17], SRPK2[18], PUS7[19], ENSTNIG00000015968[20]
