# Supplementary material for: Rapid Diversification of FoxP2 in Teleosts through Gene Duplication in the Teleost-Specific Whole Genome Duplication Event
Source: PLoS One. 2013 Dec 9;8(12):e83858. doi: 10.1371/journal.pone.0083858 (PMC3857310; doi:10.1371/journal.pone.0083858)
Supplement: Information S3 — Amino acid or fragment inserts in FoxP2 and FoxP2a of ray-finned fishes. (PDF) [file pone.0083858.s003.pdf]

Amino acid or fragment inserts in FoxP2 and FoxP2a of ray-finned fishes

Sites			25-26	37-38	245-246	258-259	275-276	286-287	305-306	329-330	460-461	489-490	636-637
Exon			2	2	6	6	7	7	7	7	11	12	16
	Lep.	*Lepisosteus oculatus*	N	N	N	N	N	N	N	N	N	Y	N
Otocephala	Cyp.	*Danio rerio*	N	N	Y	N	N	N	N	N	Y	N	Y
		Ctenopharyngodon idella	N	N	Y	N	N	N	N	N	Y	N	Y
	Cha.	*Pygocentrus nattereri*	N	N	Y	N	N	N	N	N	Y	N	Y
Protacanthopterygii	Sal.	*Salmo salar*	Y	N	Y	N	Y	Y	N	Y	Y	Y	n/a
		Oncorhynchus mykiss	n/a	n/a	n/a	n/a	n/a	n/a	n/a	n/a	n/a	Y	n/a
Neoteleostei	Bel.	*Oryzias latipes*	Y	Y	Y	Y	Y	Y	Y	Y	Y	Y	Y
	Gas.	*Gasterosteus aculeatus*	Y	Y	n/a	n/a	Y	Y	Y	Y	Y	Y	n/a
	Gad.	*Gadus morhua*	Y	Y	n/a	n/a	Y	Y	Y	Y	Y	Y	Y
	Per.	*Oreochromis niloticus*	Y	Y	Y	Y	Y	Y	Y	Y	Y	Y	Y
		Colisa lalia	Y	Y	n/a	n/a	n/a	n/a	Y	Y	Y	Y	Y
		Neolamprologus brichardi	Y	Y	Y	Y	Y	Y	Y	Y	Y	Y	Y
		Pundamilia nyererei	Y	Y	Y	Y	Y	Y	Y	Y	Y	Y	Y
		Haplochromis burtoni	Y	Y	Y	Y	Y	Y	Y	Y	Y	Y	Y
		Maylandia zebra	Y	Y	Y	Y	Y	Y	Y	Y	Y	Y	Y
	Tet.	*Takifugu rubripes*	Y	Y	Y	Y	Y	Y	Y	Y	Y	Y	Y
		Tetraodon nigroviridis	Y	Y	Y	Y	Y	Y	Y	Y	Y	Y	Y

Lep. – Lepisosteiformes; Cyp. – Cypriniformes; Cha. – Characiformes; Sal. – Salmoniformes; Bel. – Beloniformes; Gas. – Gasterosteiformes; Gad. – Gadiforms; Per. – Perciformes; Tet. – Tetraodontiformes; Y – With insert; N – Without insert; n/a – Unavailability.
