# Supplementary material for: Rapid Diversification of FoxP2 in Teleosts through Gene Duplication in the Teleost-Specific Whole Genome Duplication Event
Source: PLoS One. 2013 Dec 9;8(12):e83858. doi: 10.1371/journal.pone.0083858 (PMC3857310; doi:10.1371/journal.pone.0083858)
Supplement: information S4 — Phylogenetic reconstructions based on Data sets 1 and 2. (PDF) [file pone.0083858.s004.pdf]

S4.1 Phylogenetic reconstructions based on nucleotide sequences in Data set 1. Bootstrap values lower than 50 on branches of ML tree and posterior probability lower than 90 on branches of Bayesian tree are not shown.

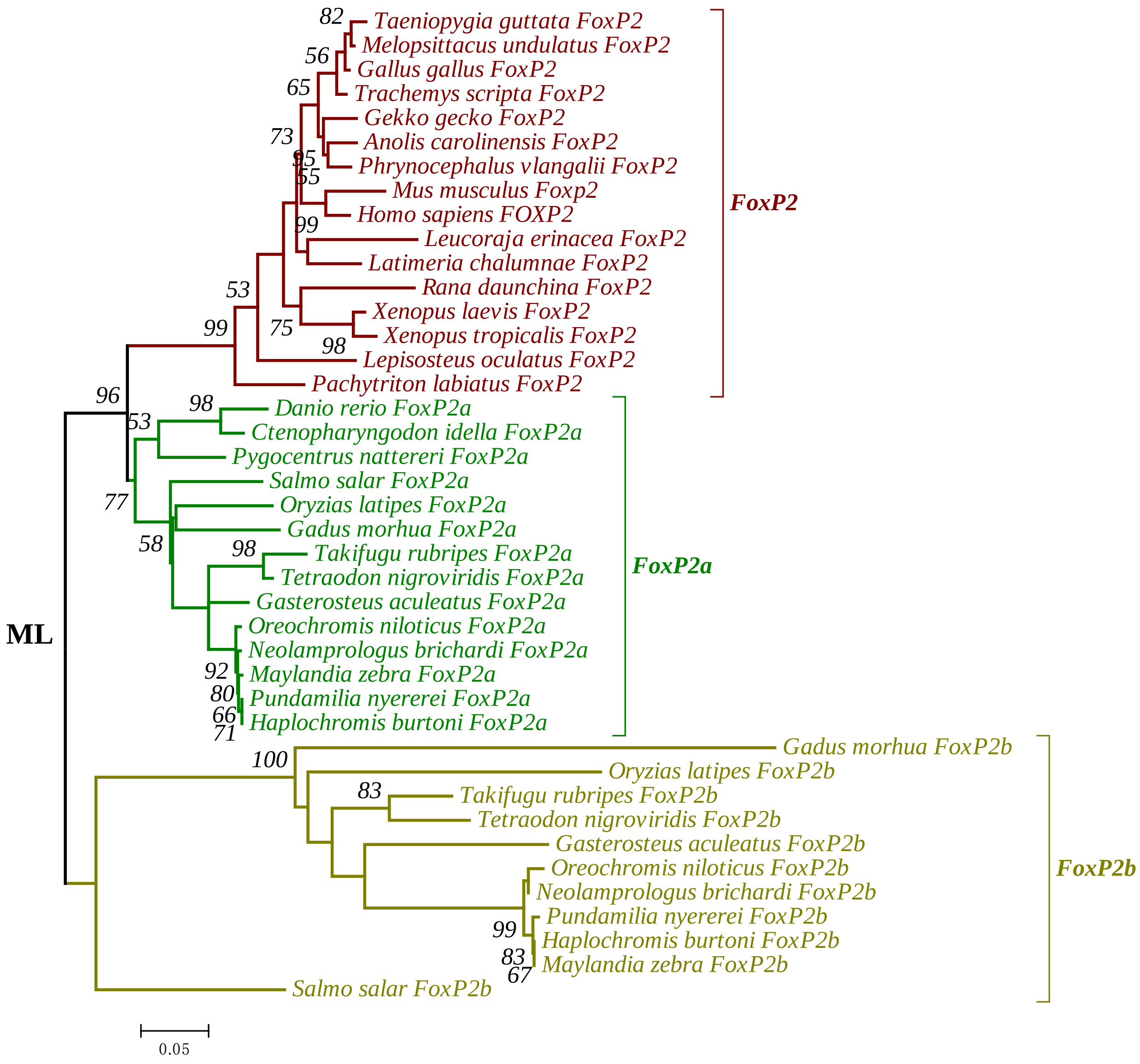

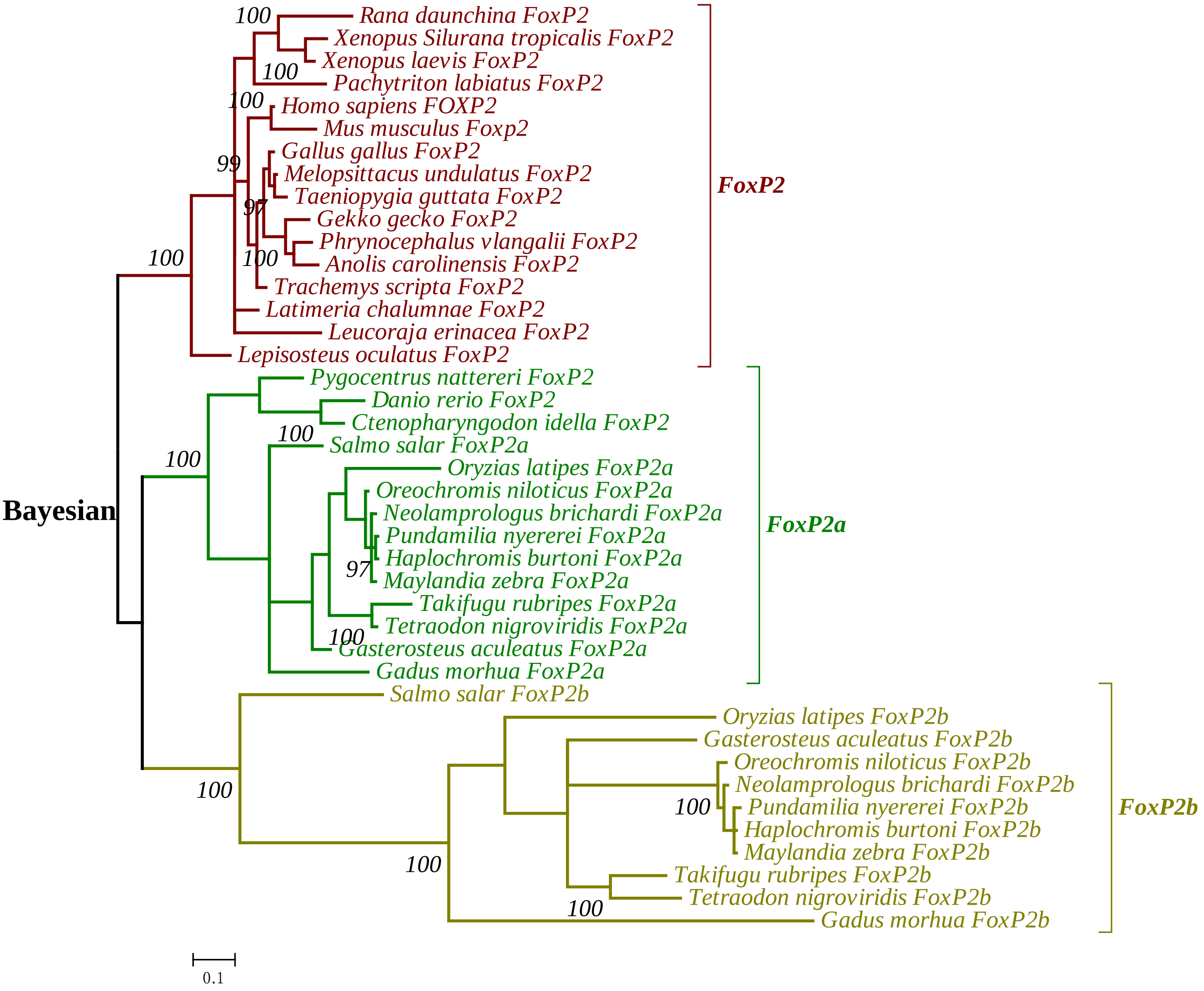

Bayesian
Rana daunchina FoxP2
Xenopus Silurana tropicalis FoxP2
Xenopus laevis FoxP2
Pachytriton labiatus FoxP2
Homo sapiens FOXP2
Mus musculus Foxp2
Gallus gallus FoxP2
Melopsittacus undulatus FoxP2
Taeniopygia guttata FoxP2
Gekko gecko FoxP2
Phrynocephalus vlangalii FoxP2
Anolis carolinensis FoxP2
Trachemys scripta FoxP2
Latimeria chalumnae FoxP2
Leucoraja erinacea FoxP2
Lepisosteus oculatus FoxP2
FoxP2
Pygocentrus nattereri FoxP2
Danio rerio FoxP2
Ctenopharyngodon idella FoxP2
Salmo salar FoxP2a
Oryzias latipes FoxP2a
Oreochromis niloticus FoxP2a
Neolamprologus brichardi FoxP2a
Pundamilia nyererei FoxP2a
Haplochromis burtoni FoxP2a
Maylandia zebra FoxP2a
Takifugu rubripes FoxP2a
Tetraodon nigroviridis FoxP2a
Gasterosteus aculeatus FoxP2a
Gadus morhua FoxP2a
FoxP2a
Salmo salar FoxP2b
Oryzias latipes FoxP2b
Gasterosteus aculeatus FoxP2b
Oreochromis niloticus FoxP2b
Neolamprologus brichardi FoxP2b
Pundamilia nyererei FoxP2b
Haplochromis burtoni FoxP2b
Maylandia zebra FoxP2b
Takifugu rubripes FoxP2b
Tetraodon nigroviridis FoxP2b
Gadus morhua FoxP2b
FoxP2b
100
99
97
0.1

S4.2 Phylogenetic reconstructions based on amino acid sequences in Data set 1. Bootstrap values lower than 50 on branches of NJ and ML trees are not shown.

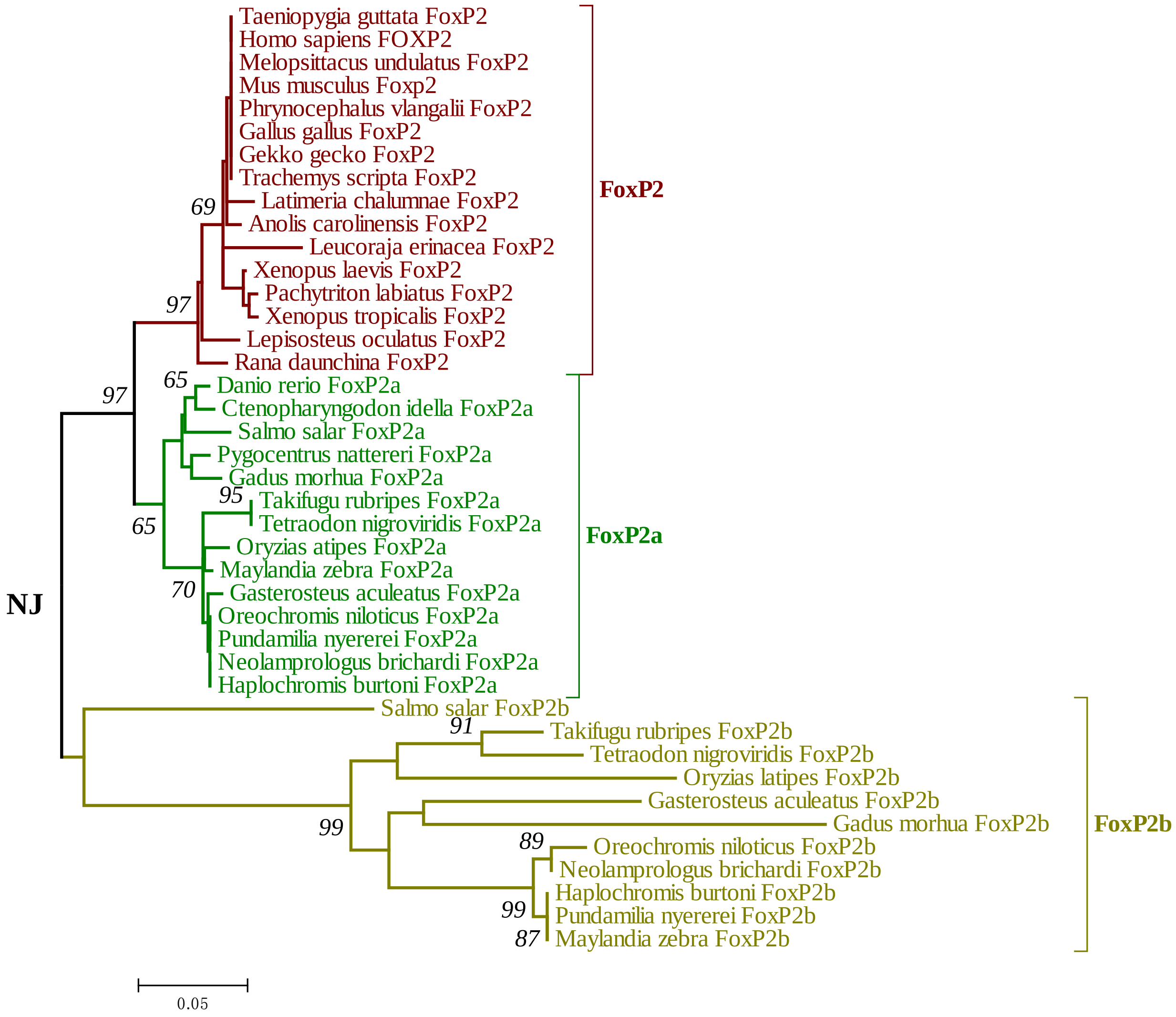

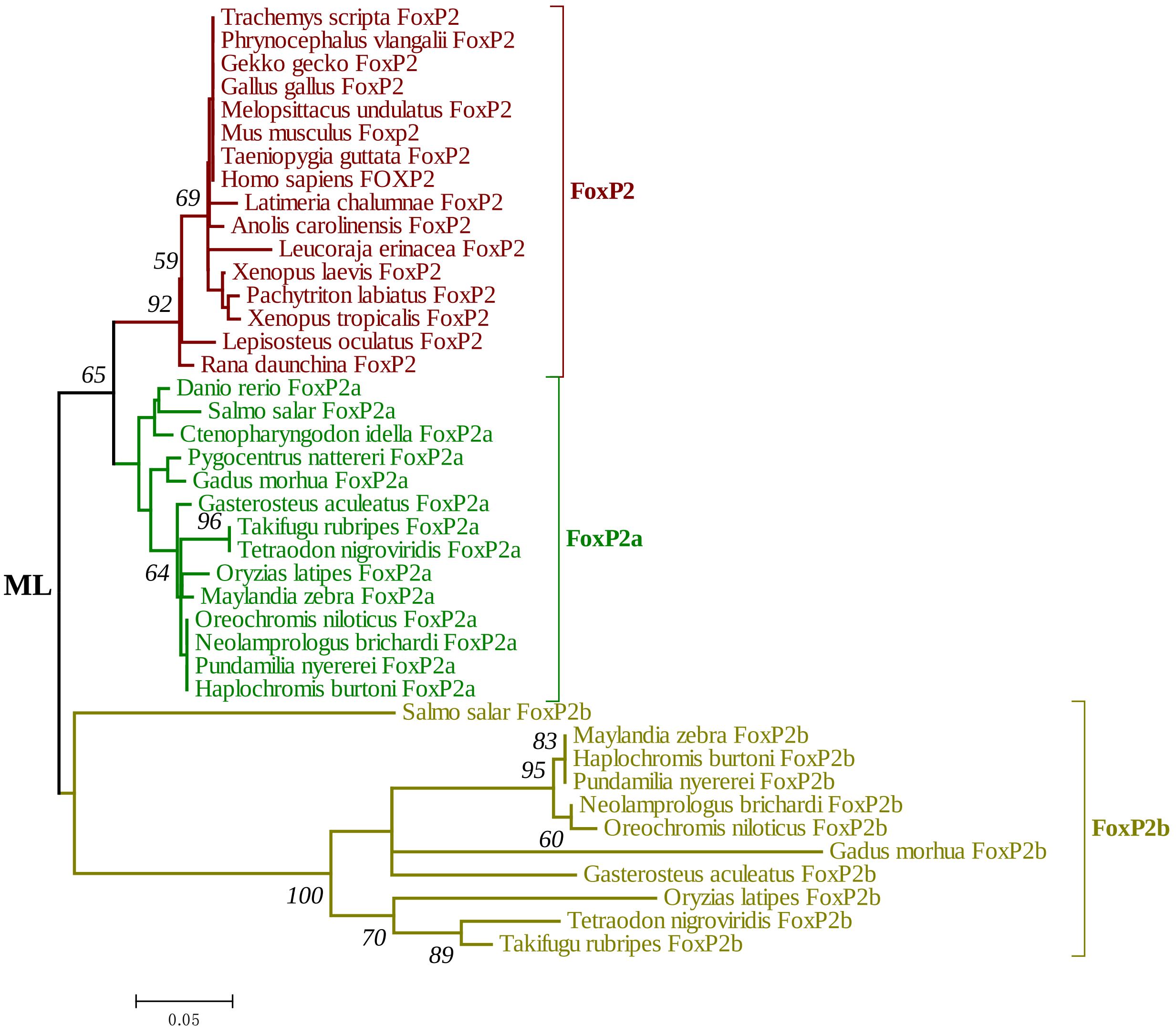
Trachemys scripta FoxP2
Phrynocephalus vlangalii FoxP2
Gekko gecko FoxP2
Gallus gallus FoxP2
Melopsittacus undulatus FoxP2
Mus musculus Foxp2
Taeniopygia guttata FoxP2
Homo sapiens FOXP2
Latimeria chalumnae FoxP2
Anolis carolinensis FoxP2
Leucoraja erinacea FoxP2
Xenopus laevis FoxP2
Pachytriton labiatus FoxP2
Xenopus tropicalis FoxP2
Lepisosteus oculatus FoxP2
Rana daunchina FoxP2
Danio rerio FoxP2a
Salmo salar FoxP2a
Ctenopharyngodon idella FoxP2a
Pygocentrus nattereri FoxP2a
Gadus morhua FoxP2a
Gasterosteus aculeatus FoxP2a
Takifugu rubripes FoxP2a
Tetraodon nigroviridis FoxP2a
Oryzias latipes FoxP2a
Maylandia zebra FoxP2a
Oreochromis niloticus FoxP2a
Neolamprologus brichardi FoxP2a
Pundamilia nyererei FoxP2a
Haplochromis burtoni FoxP2a
Salmo salar FoxP2b
Maylandia zebra FoxP2b
Haplochromis burtoni FoxP2b
Pundamilia nyererei FoxP2b
Neolamprologus brichardi FoxP2b
Oreochromis niloticus FoxP2b
Gadus morhua FoxP2b
Gasterosteus aculeatus FoxP2b
Oryzias latipes FoxP2b
Tetraodon nigroviridis FoxP2b
Takifugu rubripes FoxP2b
FoxP2
FoxP2a
FoxP2b
ML
69
59
92
65
96
64
83
95
60
100
70
89
0.05

S4.3 Phylogenetic reconstructions based on nucleotide sequences in Data set 2. Bootstrap values lower than 50 on branches of NJ and ML trees and posterior probability lower than 90 on branches of Bayesian tree are not shown.

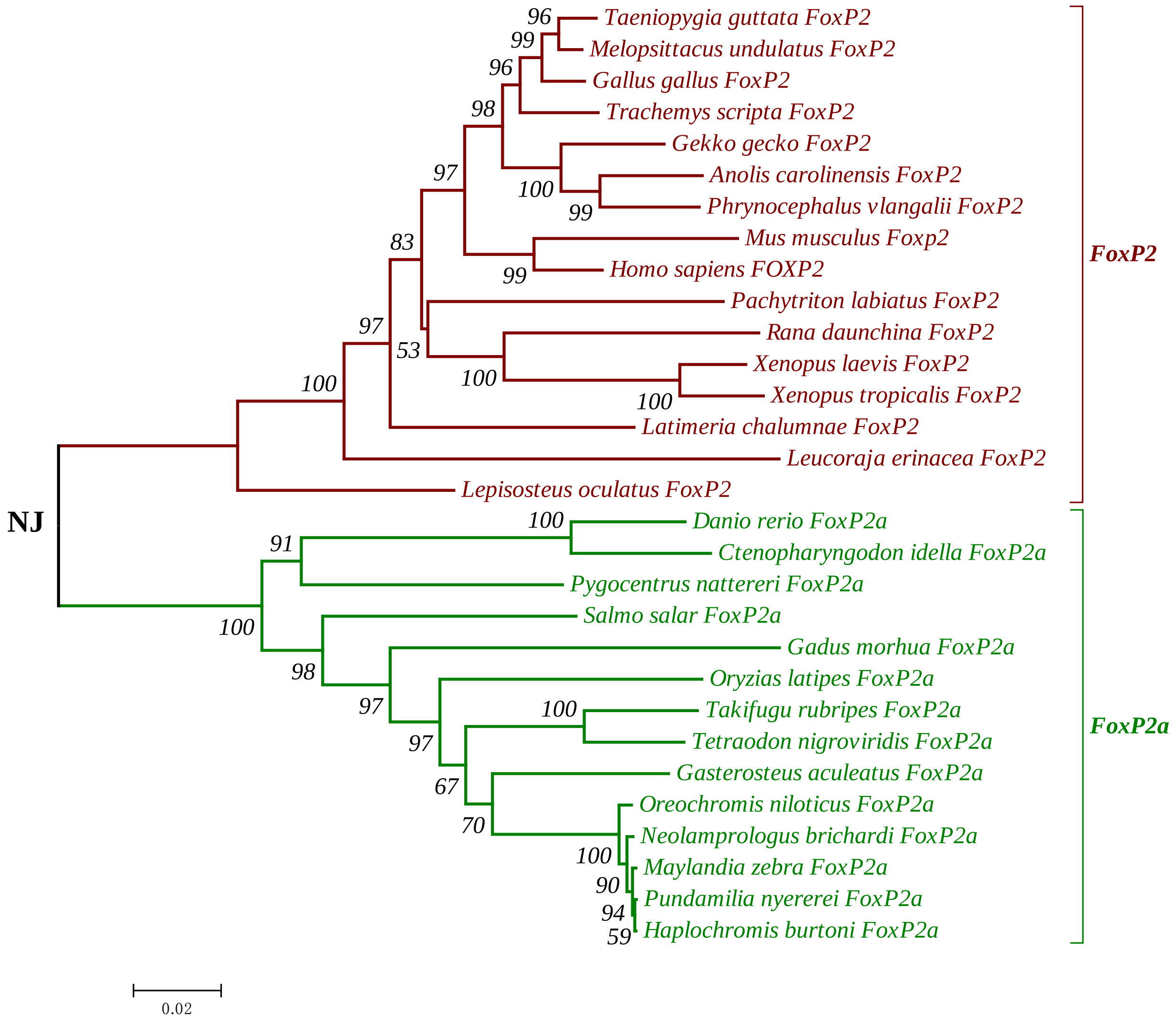

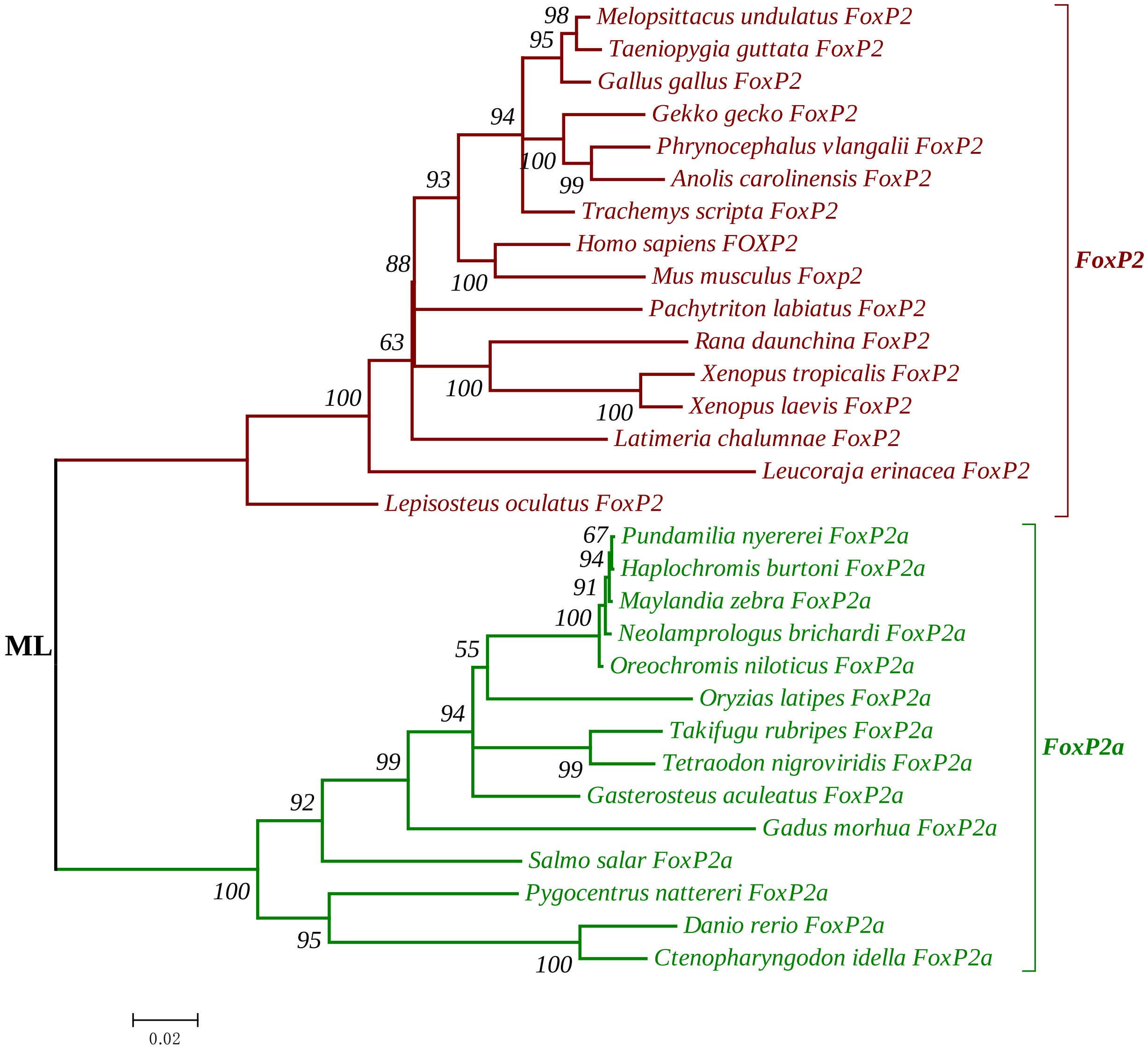

ML
98
95
Melopsittacus undulatus FoxP2
Taeniopygia guttata FoxP2
Gallus gallus FoxP2
94
Gekko gecko FoxP2
Phrynocephalus vlangalii FoxP2
100
99
Anolis carolinensis FoxP2
93
Trachemys scripta FoxP2
Homo sapiens FOXP2
88
100
Mus musculus Foxp2
Pachytriton labiatus FoxP2
63
Rana daunchina FoxP2
100
Xenopus tropicalis FoxP2
100
Xenopus laevis FoxP2
100
Latimeria chalumnae FoxP2
Leucoraja erinacea FoxP2
Lepisosteus oculatus FoxP2
FoxP2
67
Pundamilia nyererei FoxP2a
94
Haplochromis burtoni FoxP2a
91
Maylandia zebra FoxP2a
100
Neolamprologus brichardi FoxP2a
55
Oreochromis niloticus FoxP2a
Oryzias latipes FoxP2a
94
Takifugu rubripes FoxP2a
99
Tetraodon nigroviridis FoxP2a
99
Gasterosteus aculeatus FoxP2a
92
Gadus morhua FoxP2a
Salmo salar FoxP2a
100
Pygocentrus nattereri FoxP2a
95
Danio rerio FoxP2a
100
Ctenopharyngodon idella FoxP2a
FoxP2a
0.02

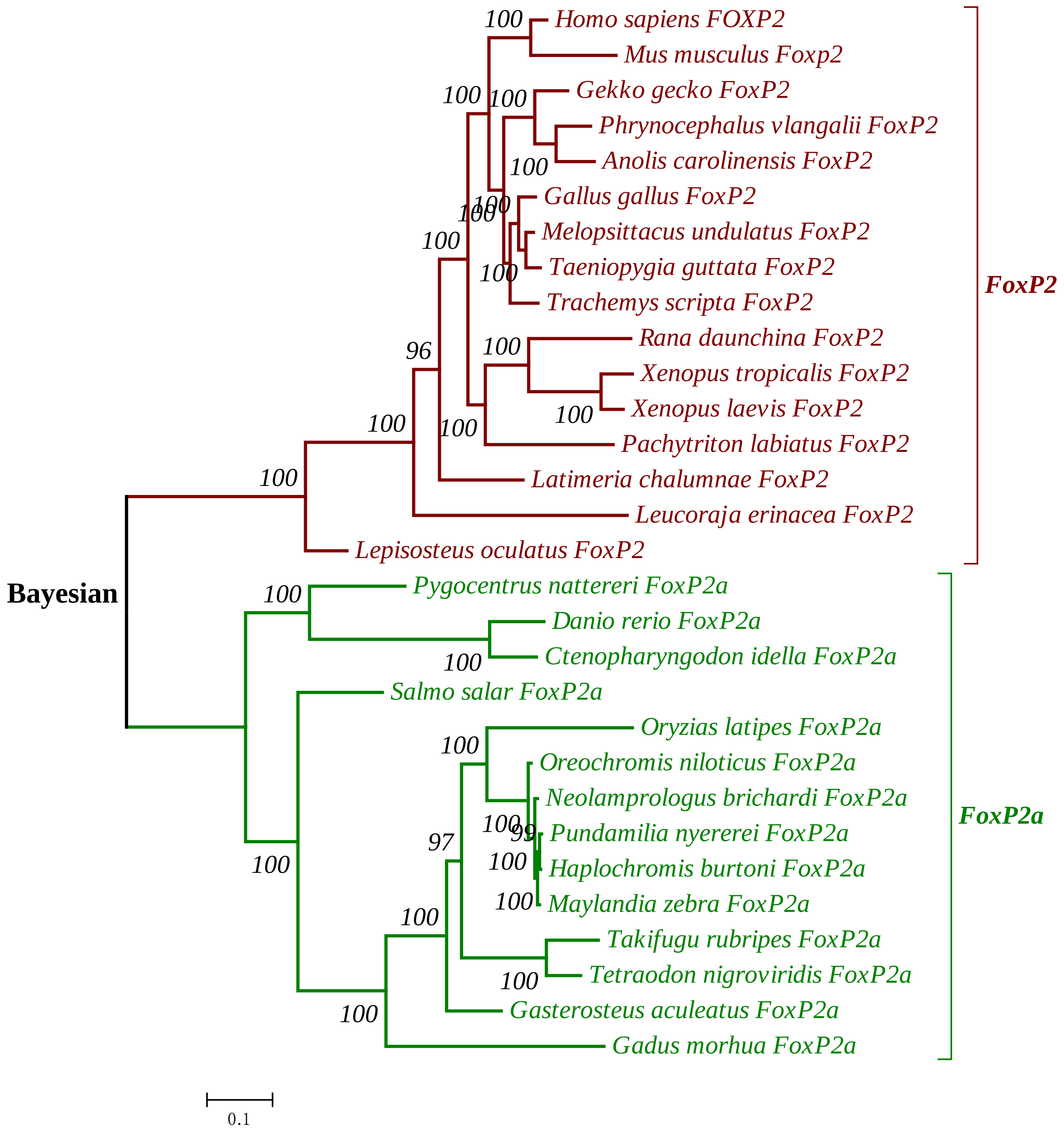
Bayesian
Homo sapiens FOXP2
Mus musculus Foxp2
Gekko gecko FoxP2
Phrynocephalus vlangalii FoxP2
Anolis carolinensis FoxP2
Gallus gallus FoxP2
Melopsittacus undulatus FoxP2
Taeniopygia guttata FoxP2
Trachemys scripta FoxP2
Rana daunchina FoxP2
Xenopus tropicalis FoxP2
Xenopus laevis FoxP2
Pachytriton labiatus FoxP2
Latimeria chalumnae FoxP2
Leucoraja erinacea FoxP2
Lepisosteus oculatus FoxP2
Pygocentrus nattereri FoxP2a
Danio rerio FoxP2a
Ctenopharyngodon idella FoxP2a
Salmo salar FoxP2a
Oryzias latipes FoxP2a
Oreochromis niloticus FoxP2a
Neolamprologus brichardi FoxP2a
Pundamilia nyererei FoxP2a
Haplochromis burtoni FoxP2a
Maylandia zebra FoxP2a
Takifugu rubripes FoxP2a
Tetraodon nigroviridis FoxP2a
Gasterosteus aculeatus FoxP2a
Gadus morhua FoxP2a
FoxP2
FoxP2a
96
97
99
100
0.1

S4.4 Phylogenetic reconstructions based on amino acid sequences in Data set 2. Bootstrap values lower than 50 on branches of NJ and ML trees are not shown.

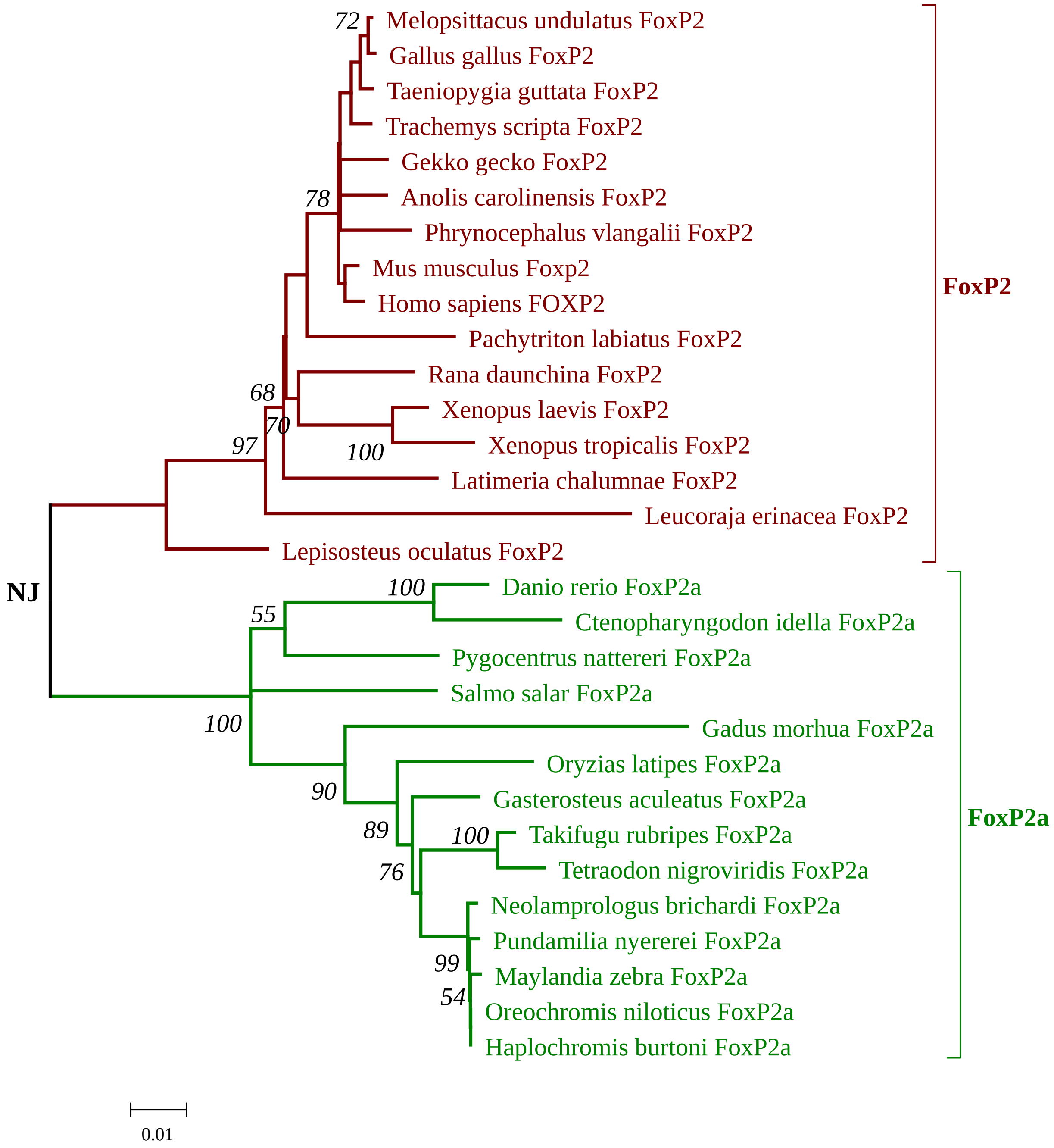

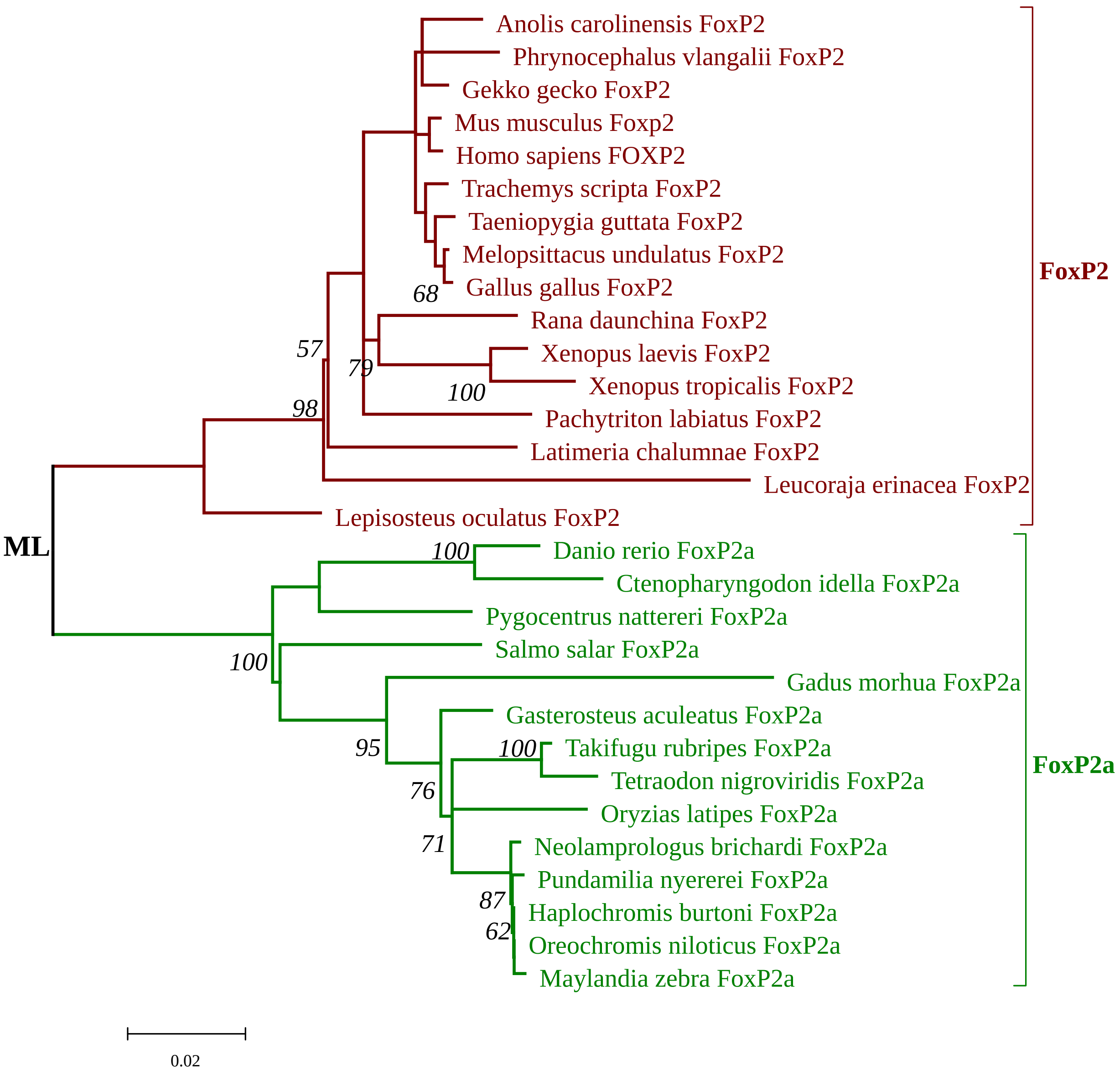
Anolis carolinensis FoxP2
Phrynocephalus vlangalii FoxP2
Gekko gecko FoxP2
Mus musculus Foxp2
Homo sapiens FOXP2
Trachemys scripta FoxP2
Taeniopygia guttata FoxP2
Melopsittacus undulatus FoxP2
Gallus gallus FoxP2
68
Rana daunchina FoxP2
57
79
Xenopus laevis FoxP2
Xenopus tropicalis FoxP2
100
98
Pachytriton labiatus FoxP2
Latimeria chalumnae FoxP2
Leucoraja erinacea FoxP2
Lepisosteus oculatus FoxP2
FoxP2
ML
100
Danio rerio FoxP2a
Ctenopharyngodon idella FoxP2a
Pygocentrus nattereri FoxP2a
100
Salmo salar FoxP2a
Gadus morhua FoxP2a
Gasterosteus aculeatus FoxP2a
95
100
Takifugu rubripes FoxP2a
Tetraodon nigroviridis FoxP2a
76
Oryzias latipes FoxP2a
71
Neolamprologus brichardi FoxP2a
Pundamilia nyererei FoxP2a
87
Haplochromis burtoni FoxP2a
62
Oreochromis niloticus FoxP2a
Maylandia zebra FoxP2a
FoxP2a
0.02
