# Supplementary material for: Rapid Diversification of FoxP2 in Teleosts through Gene Duplication in the Teleost-Specific Whole Genome Duplication Event
Source: PLoS One. 2013 Dec 9;8(12):e83858. doi: 10.1371/journal.pone.0083858 (PMC3857310; doi:10.1371/journal.pone.0083858)

S5.1

Posterior probability profiles of site-specific rate difference among clusters in Data set 1. The amino acid sites are original in the sequence alignment.

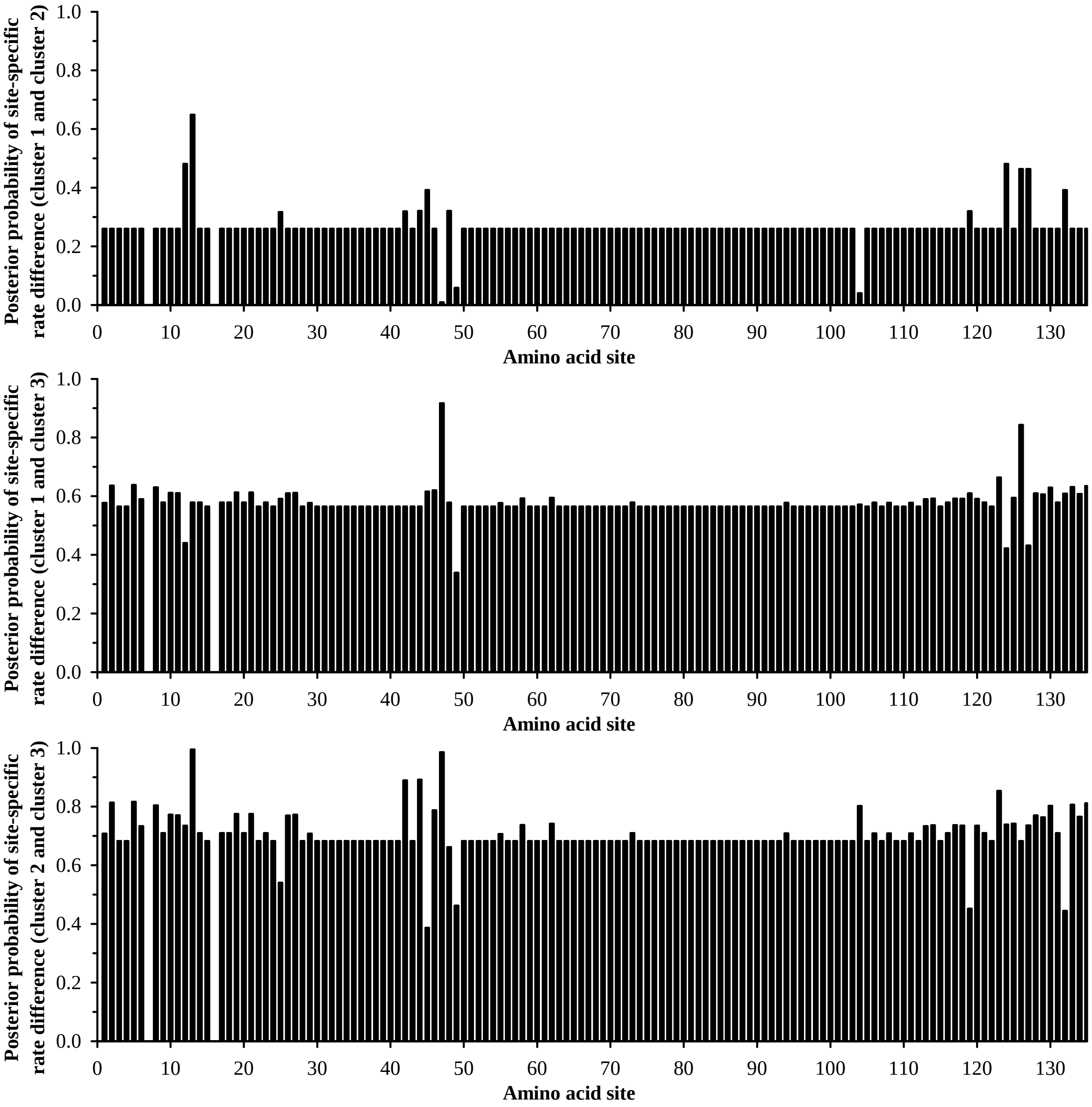

S5.2

Posterior probability profiles of site-specific rate difference among clusters in Data set 2. The amino acid sites are original in the sequence alignment.

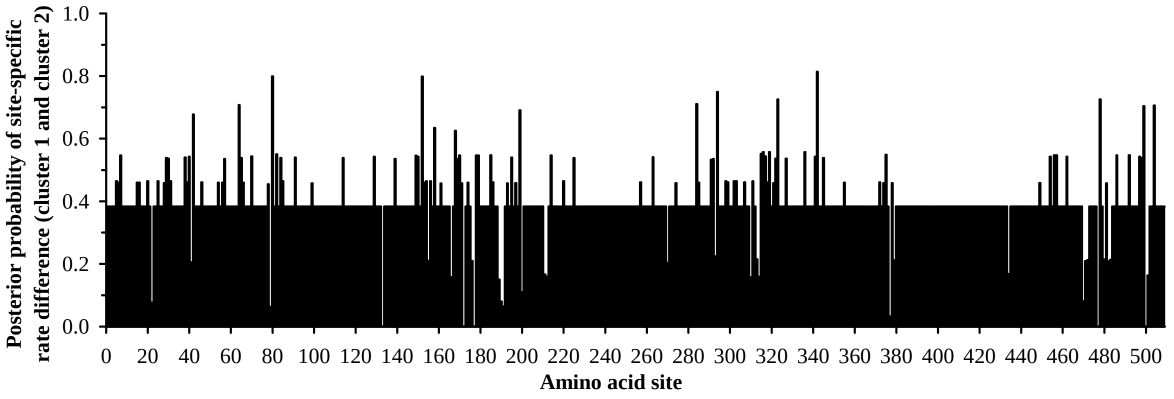

Supplement: Information S5 — Posterior probability profiles of site-specific rate difference among clusters in Data sets 1 and 2. (PDF) [file pone.0083858.s005.pdf]
