# Supplementary material for: Rapid Diversification of FoxP2 in Teleosts through Gene Duplication in the Teleost-Specific Whole Genome Duplication Event
Source: PLoS One. 2013 Dec 9;8(12):e83858. doi: 10.1371/journal.pone.0083858 (PMC3857310; doi:10.1371/journal.pone.0083858)
Supplement: Information S6 — Hierarchical dendrograms of genes in Data sets 1 and 2. (PDF) [file pone.0083858.s006.pdf]

S6.1 Four hierarchical dendrograms of genes from Data set 1. FoxP2, FoxP2a and FoxP2b are enclosed with blue, red and green dotted-rectangles, respectively.

Symbols	Genes
1	Leucoraja erinacea FoxP2
2	Lepisosteus oculatus FoxP2
3	Pygocentrus nattereri FoxP2a
4	Danio rerio FoxP2a
5	Ctenopharyngodon idella FoxP2a
6	Salmo salar FoxP2a
7	Salmo salar FoxP2b
8	Oryzias latipes FoxP2a
9	Oryzias latipes FoxP2b
10	Gasterosteus aculeatus FoxP2a
11	Gasterosteus aculeatus FoxP2b
12	Gadus morhua FoxP2a
13	Gadus morhua FoxP2b
14	Oreochromis niloticus FoxP2a
15	Oreochromis niloticus FoxP2b
16	Neolamprologus brichardi FoxP2a
17	Neolamprologus brichardi FoxP2b
18	Pundamilia nyererei FoxP2a
19	Pundamilia nyererei FoxP2b
20	Haplochromis burtoni FoxP2a
21	Haplochromis burtoni FoxP2b
22	Maylandia zebra FoxP2a
23	Maylandia zebra FoxP2b
24	Takifugu rubripes FoxP2a
25	Takifugu rubripes FoxP2b
26	Tetraodon nigroviridis FoxP2a
27	Tetraodon nigroviridis FoxP2b
28	Latimeria chalumnae FoxP2
29	Pachytriton labiatus FoxP2
30	Xenopus laevis FoxP2
31	Xenopus tropicalis FoxP2
32	Rana daunchina FoxP2
33	Trachemys scripta FoxP2
34	Anolis carolinensis FoxP2
35	Phrynocephalus vlangalii FoxP2
36	Gekko gecko FoxP2
37	Taeniopygia guttata FoxP2
38	Melopsittacus undulatus FoxP2
39	Gallus gallus FoxP2
40	Mus musculus Foxp2
41	Homo sapiens FOXP2

Dendrogram using average linkage of cityblock distances between groups

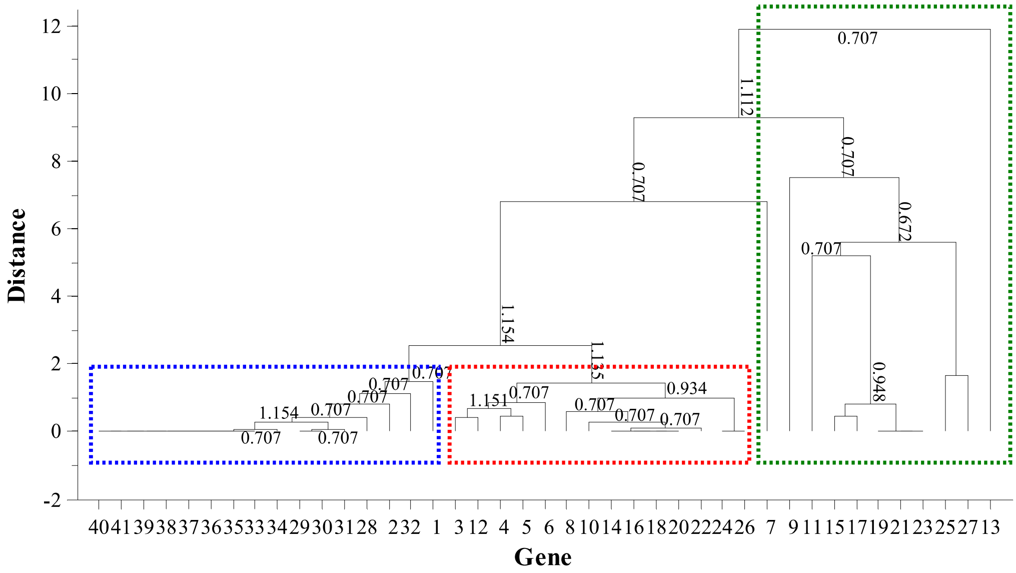

Dendrogram using average linkage of Pearson correlation distances between groups

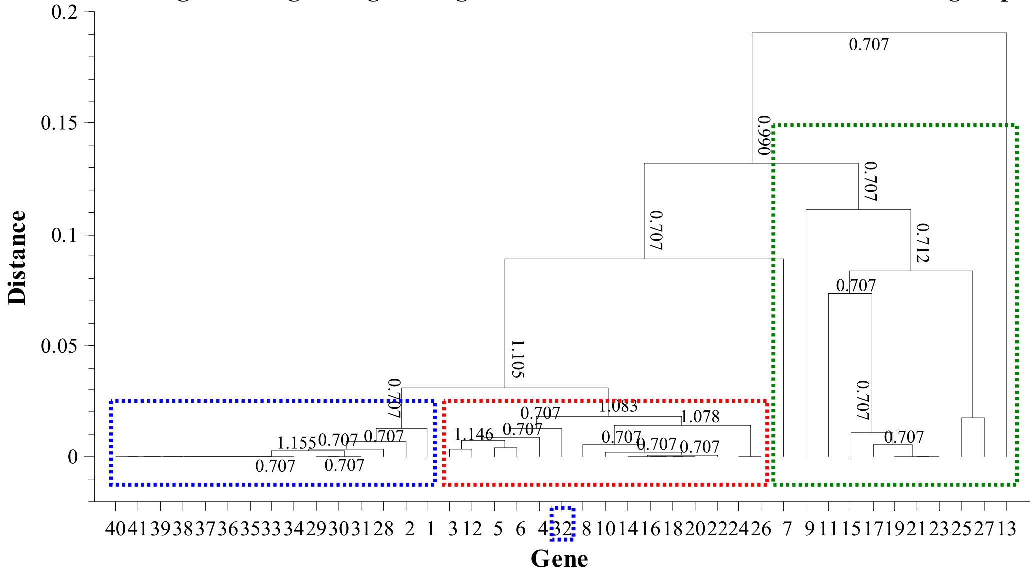

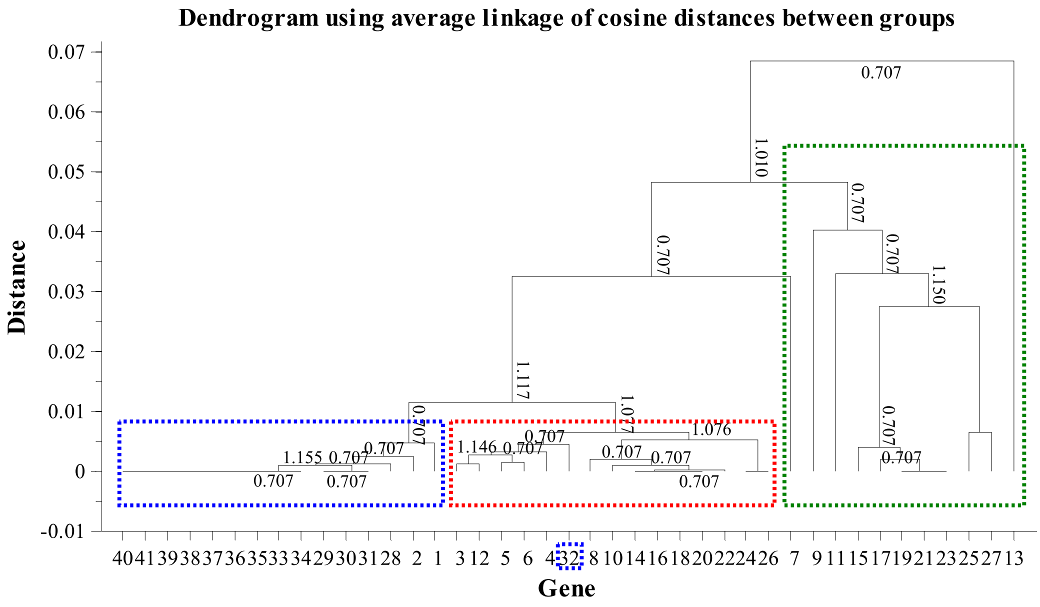
Dendrogram using average linkage of cosine distances between groups
Distance
Gene
0.07
0.06
0.05
0.04
0.03
0.02
0.01
0
-0.01
40 41 39 38 37 36 35 33 34 29 30 31 28 2 1 3 12 5 6 4 32 8 10 14 16 18 20 22 24 26 7 9 11 15 17 19 21 23 25 27 13
0.707
1.010
0.707
0.707
0.707
1.150
1.117
1.027
1.076
0.707
1.155
0.707
0.707
0.707
0.707
1.146
0.707
0.707
0.707
0.707
0.707
0.707
0.707

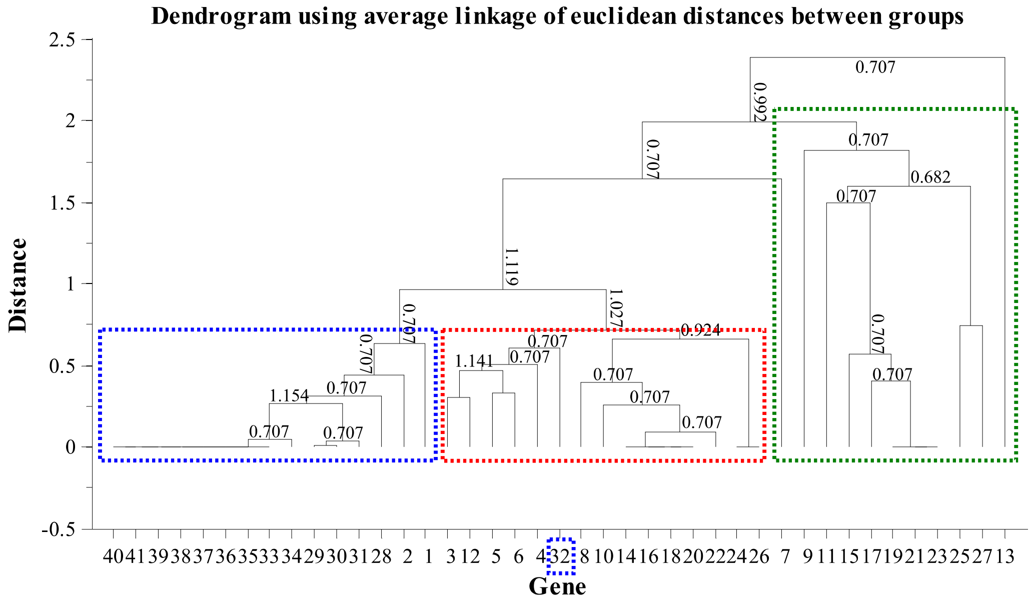
Dendrogram using average linkage of euclidean distances between groups
Distance
Gene
2.5
2
1.5
1
0.5
0
-0.5
40 41 39 38 37 36 35 33 34 29 30 31 28 2 1 3 12 5 6 4 32 8 10 14 16 18 20 22 24 26 7 9 11 15 17 19 21 23 25 27 13
0.707
0.992
0.707
0.707
0.682
0.707
1.119
1.027
0.924
0.707
0.707
1.141
0.707
0.707
1.154
0.707
0.707
0.707
0.707
0.707
0.707
0.707
0.707

S6.2 Four hierarchical dendrograms of genes from Data set 2. FoxP2 and FoxP2a are enclosed with blue and red dotted-rectangles, respectively.

Symbols	Genes
1	Leucoraja erinacea FoxP2
2	Lepisosteus oculatus FoxP2
3	Pygocentrus nattereri FoxP2a
4	Danio rerio FoxP2a
5	Ctenopharyngodon idella FoxP2a
6	Salmo salar FoxP2a
7	Oryzias latipes FoxP2a
8	Gasterosteus aculeatus FoxP2a
9	Gadus morhua FoxP2a
10	Oreochromis niloticus FoxP2a
11	Neolamprologus brichardi FoxP2a
12	Pundamilia nyererei FoxP2a
13	Haplochromis burtoni FoxP2a
14	Maylandia zebra FoxP2a
15	Takifugu rubripes FoxP2a
16	Tetraodon nigroviridis FoxP2a
17	Latimeria chalumnae FoxP2
18	Pachytriton labiatus FoxP2
19	Xenopus laevis FoxP2
20	Xenopus tropicalis FoxP2
21	Rana daunchina FoxP2
22	Trachemys scripta FoxP2
23	Anolis carolinensis FoxP2
24	Phrynocephalus vlangalii FoxP2
25	Gekko gecko FoxP2
26	Taeniopygia guttata FoxP2
27	Melopsittacus undulatus FoxP2
28	Gallus gallus FoxP2
29	Mus musculus Foxp2
30	Homo sapiens FOXP2

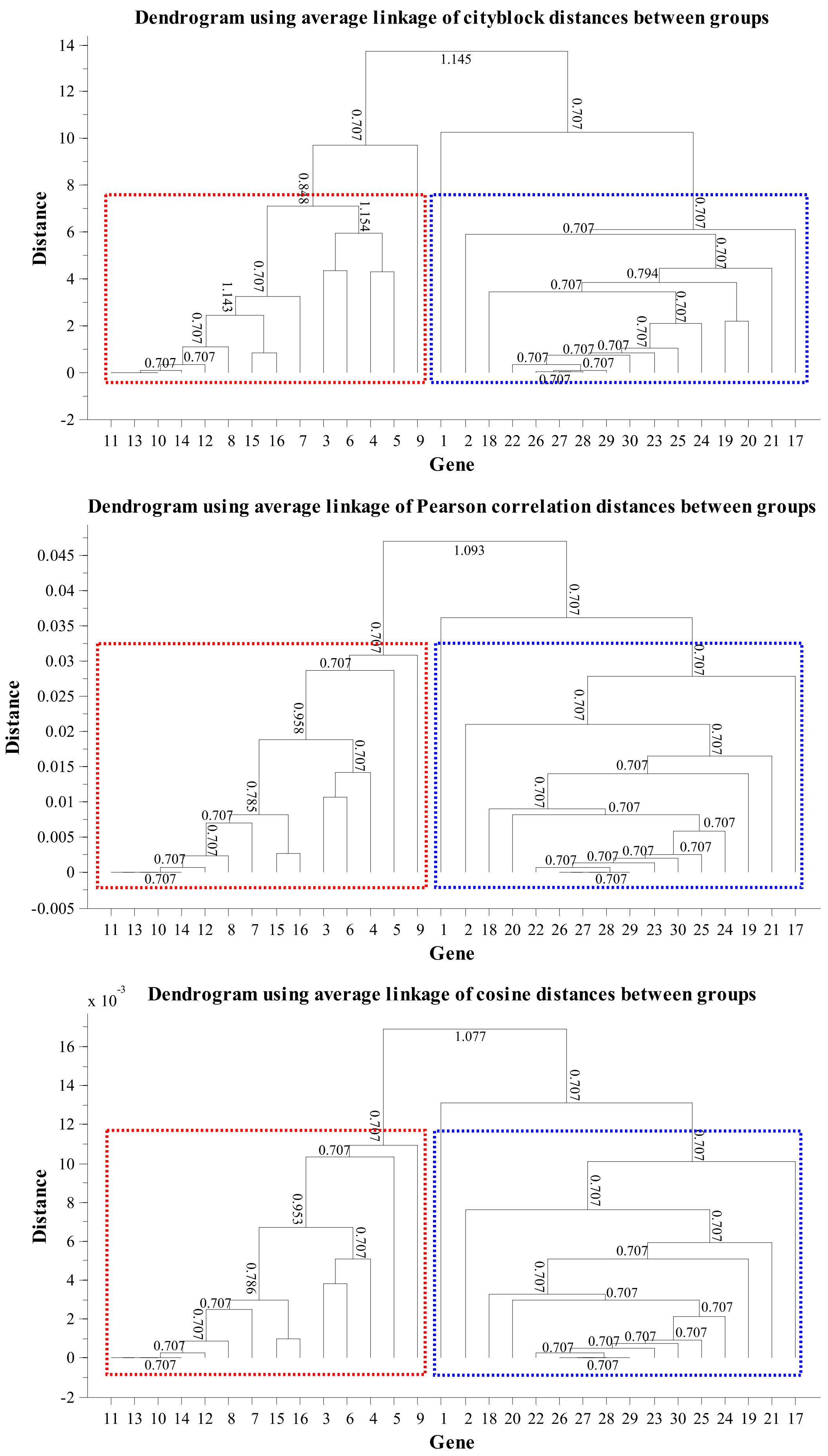
Dendrogram using average linkage of cityblock distances between groups
Distance
Gene
11 13 10 14 12 8 15 16 7 3 6 4 5 9 1 2 18 22 26 27 28 29 30 23 25 24 19 20 21 17
1.145
0.707
0.848
1.154
0.794
1.143
Dendrogram using average linkage of Pearson correlation distances between groups
Distance
Gene
11 13 10 14 12 8 7 15 16 3 6 4 5 9 1 2 18 20 22 26 27 28 29 23 30 25 24 19 21 17
1.093
0.707
0.958
0.785
x 10^-3
Dendrogram using average linkage of cosine distances between groups
Distance
Gene
11 13 10 14 12 8 7 15 16 3 6 4 5 9 1 2 18 20 22 26 27 28 29 23 30 25 24 19 21 17
1.077
0.707
0.953
0.786

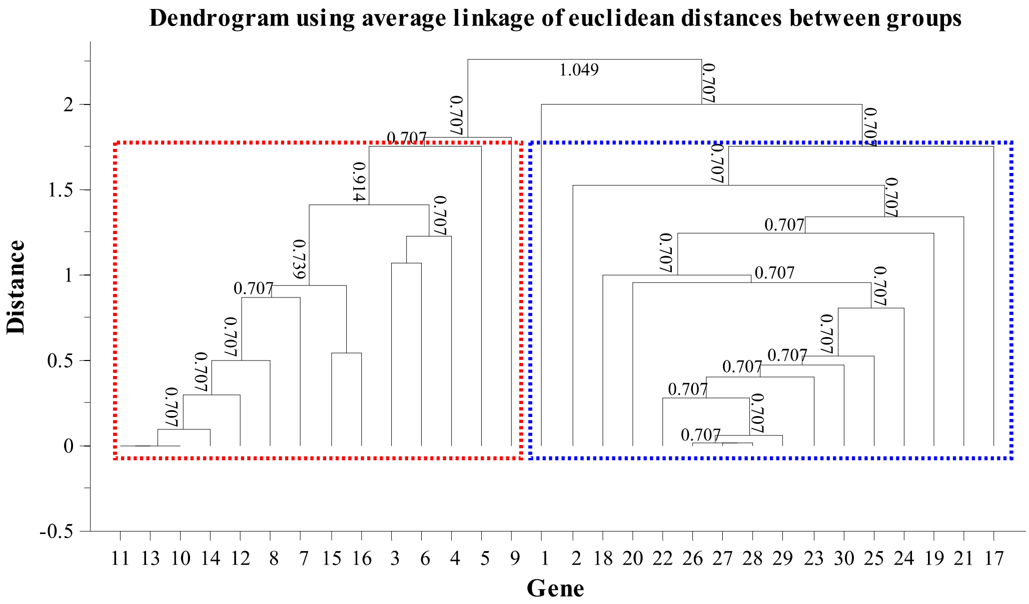
Dendrogram using average linkage of euclidean distances between groups
Distance
Gene
2
1.5
1
0.5
0
-0.5
11 13 10 14 12 8 7 15 16 3 6 4 5 9 1 2 18 20 22 26 27 28 29 23 30 25 24 19 21 17
1.049
0.707
0.914
0.739
