# Supplementary material for: Rapid Diversification of FoxP2 in Teleosts through Gene Duplication in the Teleost-Specific Whole Genome Duplication Event
Source: PLoS One. 2013 Dec 9;8(12):e83858. doi: 10.1371/journal.pone.0083858 (PMC3857310; doi:10.1371/journal.pone.0083858)
Supplement: Information S7 — Test of natural selection pressures on Data sets 1 and 2. (PDF) [file pone.0083858.s007.pdf]

S7.1 Test of natural selection pressures on Data set 1

Table S7.1.1 Log likelihood values and estimates of parameters under site models

Site models	*p*	*l*	Estimates of parameters	Positively selected sites
Model 0: one-ratio	81	-4582.871	l= 10.748, κ= 1.920, N= 306.2, S= 104.8, ω= 0.064	—
Model 3: discrete	85	-4471.755	l= 11.253, κ= 1.926, N= 306.1, S= 104.9, p0= 0.535, p1= 0.457, p2= 0.007, ω0= 0, ω1= 0.130, ω2= 1.444	521S**
Model 1: nearly neutral	82	-4551.304	l= 10.913, κ= 1.928, N= 306.1, S= 104.9, p0= 0.988, p1= 0.012, ω0= 0.058, ω1= 1	—
Model 2: positive selection	84	-4551.304	l= 10.913, κ= 1.928, N= 306.1, S= 104.9, p0= 0.988, p1= 0.012, p2= 0, ω0= 0.046, ω1= 1	—
Model 7: beta	82	-4485.914	l= 11.404, κ= 1.927, N= 306.1, S= 104.9, p= 0.288, q= 3.394	—
Model 8: beta and ω>1	84	-4477.387	l= 11.274, κ= 1.938, N= 306.0, S= 105.0, p= 0.331, q= 5.218, p0= 0.993, p1= 0.007, ω= 1.459	521S

Note— *p* is the number of parameters in the model. *l* and l are log likelihood value and branch length respectively. Positively selected sites were changed to be corresponding sites of FOXP2 of human being. ** Posterior probability (ω > 1) > 0.99.

Table S7.1.2 Log likelihood values and estimates of parameters under branch specific models

Branch specific models	*p*	*l*	Estimates of parameters
ω1(Cluster 1) = ω1(Cluster 2)	82	-4549.326	l= 9.967, κ= 1.876, N= 306.6, S= 104.4, ω0= 0.118, ω1= 0.026
ω1(Cluster 1), ω2(Cluster 2)	83	-4548.996	l= 9.958, κ= 1.876, N= 306.6, S= 104.4, ω0= 0.118, ω1= 0.022, ω2= 0.029
ω1(Cluster 1) = ω1(Cluster 3)	82	-4571.290	l= 10.312, κ= 1.899, N= 306.4, S= 104.6, ω0= 0.036, ω1= 0.087
ω1(Cluster 1), ω2(Cluster 3)	83	-4545.178	l= 9.832, κ= 1.870, N= 306.6, S= 104.4, ω0= 0.035, ω1= 0.022, ω2= 0.139
ω1(Cluster 2) = ω1(Cluster 3)	82	-4571.726	l= 10.303, κ= 1.899, N= 306.4, S= 104.6, ω0= 0.031, ω1= 0.082
ω1(Cluster 2), ω2(Cluster 3)	83	-4546.247	l= 9.849, κ= 1.871, N= 306.6, S= 104.4, ω0= 0.032, ω1= 0.030, ω2= 0.139

Note— *p* is the number of parameters in the model. *l* and l are log likelihood value and branch length respectively.

Table S7.1.3 Likelihood ratio test of site and branch specific models

Alternative model	Nested model	*df*	2Δ*l*
free models	Model 0	78	158.362**
Model 3	Model 0	4	222.232**
Model 2	Model 1	2	0
Model 8	Model 7	2	17.054**
ω1(Cluster 1), ω2(Cluster 2)	ω1(Cluster 1) = ω1(Cluster 2)	1	0.66
ω1(Cluster 1), ω2(Cluster 3)	ω1(Cluster 1) = ω1(Cluster 3)	1	52.224**
ω1(Cluster 2), ω2(Cluster 3)	ω1(Cluster 2) = ω1(Cluster 3)	1	50.958**

** P < 0.01

Table S7.1.4 Analysis of branch-site models A

Foreground lineages (node number)	*l* for model A	*l* for m1a (nearly neural model)	*l* for null model A	*p* value for Test 1	*p* value for Test 2	Positively selected sites for foreground lineages by Bayes Empirical Bayes (BEB) analysis
FoxP2 in ancestor of Sauropsida (5)	-4551.304	-4551.304	—	1	—	—
FoxP2 in ancestor of Mammalia (11)	-4551.304	-4551.304	—	1	—	—
FoxP2 in ancestor of Amniota (4)	-4551.304	-4551.304	—	1	—	—
FoxP2 in ancestor of Amphibia (12)	-4551.304	-4551.304	—	1	—	—
FoxP2 in ancestor of Tetrapoda (3)	-4551.304	-4551.304	—	1	—	—
FoxP2 in ancestor of Sarcopterygii (2)	-4551.304	-4551.304	—	1	—	—
FoxP2 in ancestor of Actinopterygii (15)	-4551.265	-4551.304	—	0.962	—	—
FoxP2 in ancestor of Teleostei (16)	-4551.304	-4551.304	—	1	—	—
FoxP2a in ancestor of Teleostei (17)	-4551.304	-4551.304	—	1	—	—
FoxP2a in ancestor of Otocephala (28)	-4551.304	-4551.304	—	1	—	—
FoxP2a in ancestor of Cypriniformes (29)	-4551.304	-4551.304	—	1	—	—
FoxP2a in ancestor of Euteleostei (18)	-4551.304	-4551.304	—	1	—	—
FoxP2a in ancestor of Neoteleostei (19)	-4551.304	-4551.304	—	1	—	—
FoxP2a in ancestor of Acanthopterygii (20)	-4550.931	-4551.304	—	0.689	—	—
FoxP2a in ancestor of Percomorpha (21)	-4551.304	-4551.304	—	1	—	—
FoxP2a in ancestor of Unnamed (22)	-4551.304	-4551.304	—	1	—	—
FoxP2a in ancestor of Tetraodontiformes (27)	-4543.441	-4551.304	-4547.334	< 0.001	0.005	523R**
FoxP2a in ancestor of Perciformes (23)	-4551.304	-4551.304	—	1	—	—
FoxP2b in ancestor of Euteleostei (30)	-4548.482	-4551.304	—	0.059	—	—
FoxP2b in ancestor of Neoteleostei (31)	-4539.471	-4551.304	-4542.104	< 0.001	0.022	481D,482K**,483Y*,484N,491I,492A*,493P**, 494N**,501A,532S,595V,597N,598I,604Y,607A,609N

FoxP2b in ancestor of Acanthopterygii (32)	-4550.003	-4551.304	—	0.272	—	—
FoxP2b in ancestor of Percomorpha (33)	-4549.552	-4551.304	—	0.173	—	—
FoxP2b in ancestor of Unnamed (34)	-4548.864	-4551.304	—	0.087	—	—
FoxP2b in ancestor of Tetraodontiformes (39)	-4546.333	-4551.304	-4546.621	0.007	0.448	478R*,481D*,501A*,523R*,592P
FoxP2b in ancestor of Perciformes (35)	-4548.741	-4551.304	—	0.077	—	—

Note— p is one tail probability for χ^2 distribution. If p value in Test 1 was not up to significant level ($P < 0.05$), we neglected Test 2 and positively selected sites. The sites were taken place by corresponding sites of human FOXP2. * Posterior probability ($\omega > 1$) > 0.95; ** Posterior probability ($\omega > 1$) > 0.99.

S7.2 Test of natural selection pressures on Data set 2

Table S7.2.1 Log likelihood values and estimates of parameters under site models

Site models	*p*	*l*	Estimates of parameters	Positively selected sites
Model 0: one-ratio	59	-11868.170	l = 5.710, κ= 2.064, N= 1123.3, S= 418.7, ω= 0.059	—
Model 3: discrete	63	-11663.570	l= 5.779, κ= 2.029, N= 1124.5, S= 417.5, p0= 0.733, p1= 0.233, p2= 0.034, ω0= 0.009, ω1= 0.156, ω2= 0.632	—
Model 1: nearly neutral	60	-11739.893	l= 5.727, κ= 2.133, N= 1121.1, S= 420.9, p0= 0.948, p1= 0.052, ω0= 0.039, ω1= 1	—
Model 2: positive selection	62	-11739.893	l= 5.727, κ= 2.133, N= 1121.1, S= 420.9, p0= 0.948, p1=0.052, p2= 0, ω0= 0.039, ω1= 1	—
Model 7: beta	60	-11670.413	l= 5.852, κ= 2.026, N= 1124.6, S= 417.4, p= 0.210, q= 2.614	—
Model 8: beta and ω>1	62	-11659.330	l= 5.828, κ= 2.028, N= 1124.5, S= 417.5, p= 0.225, q= 3.085, p0= 0.998, p1= 0.002, ω= 2.312	521S**

Note— *p* is the number of parameters in the model. *l* and l are log likelihood value and branch length respectively. Positively selected sites were changed into corresponding sites of FOXP2 of human being. * Posterior probability (ω > 1) > 0.95; ** Posterior probability (ω > 1) > 0.99.

Table S7.2.2 Log likelihood values and estimates of parameters under branch specific models

Branch specific models	*p*	*l*	Estimates of parameters
ω1(Cluster 1) = ω1(Cluster 2)	60	-11865.032	l= 5.702, κ= 2.064, N= 1123.3, S= 418.7, ω0= 0.087, ω1= 0.056
ω1(Cluster 1), ω2(Cluster 2)	61	-11862.056	l= 5.682, κ= 2.061, N= 1123.4, S= 418.6, ω0= 0.088, ω1= 0.046, ω2= 0.063

Table S7.2.3 Likelihood ratio test of site and branch specific models

Alternative model	Nested model	*df*	2Δ*l*
free models	Model 0	50	123.63**
Model 3	Model 0	4	422.756**
Model 2	Model 1	2	0
Model 8	Model 7	2	14.408**
ω1(Cluster 1), ω2(Cluster 2)	ω1(Cluster 1) = ω1(Cluster 2)	1	5.952*

* P < 0.05; ** P < 0.01

Table S7.2.4 Analysis of branch-site models A

Foreground lineages (node number)	*l* for model A	*l* for m1a (nearly neural model)	*l* for null model A	*p* value for Test 1	*p* value for Test 2	Positively selected sites for foreground lineages by Bayes Empirical Bayes (BEB) analysis
FoxP2 in ancestor of Sauropsida (23)	-11739.893	-11739.893	—	1	—	—
FoxP2 in ancestor of Mammalia (22)	-11739.893	-11739.893	—	1	—	—
FoxP2 in ancestor of Amniota (21)	-11739.893	-11739.893	—	1	—	—
FoxP2 in ancestor of Amphibia (18)	-11739.893	-11739.893	—	1	—	—
FoxP2 in ancestor of Tetrapoda (17)	-11739.893	-11739.893	—	1	—	—
FoxP2 in ancestor of Sarcopterygii (16)	-11739.893	-11739.893	—	1	—	—
FoxP2 in ancestor of Actinopterygii (2)	-11735.063	-11739.893	-11738.336	0.008	0.010	436L, 457I, 598I, 709P*
FoxP2a in ancestor of Teleostei (3)	-11736.760	-11739.893	-11736.760	0.044	1	437E, 460G, 484N
FoxP2a in ancestor of Otocephala (14)	-11739.893	-11739.893	—	1	—	—
FoxP2a in ancestor of Euteleostei (4)	-11739.893	-11739.893	—	1	—	—
FoxP2a in ancestor of Cypriniformes (15)	-11734.552	-11739.893	-11738.433	0.005	0.005	275G, 296S, 462S*
FoxP2a in ancestor of Neoteleostei (5)	-11736.651	-11739.893	-11738.803	0.039	0.038	304T, 305S, 418P, 459Q, 465T, 466P
FoxP2a in ancestor of Acanthopterygii (6)	-11739.448	-11739.893	—	0.641	—	—
FoxP2a in ancestor of Percomorpha (7)	-11739.893	-11739.893	—	1	—	—
FoxP2a in ancestor of Tetraodontiformes (13)	-11731.258	-11739.893	-11735.452	< 0.001	0.004	323V*, 523R**
FoxP2a in ancestor of Unnamed (8)	-11739.893	-11739.893	—	1	—	—
FoxP2a in ancestor of Perciformes (9)	-11739.893	-11739.893	—	1	—	—

Note— *p* is one tail probability for χ^2 distribution. If *p* value in Test 1 was not up to significant level (P<0.05), we neglected Test 2 and positively selected sites. The sites were taken place by corresponding sites of human FOXP2. * Posterior probability ($\omega > 1$) > 0.95; ** Posterior probability ($\omega > 1$) > 0.99.
